# Genome-Wide DNA Methylation Analysis of Human Pancreatic Islets from Type 2 Diabetic and Non-Diabetic Donors Identifies Candidate Genes That Influence Insulin Secretion

**DOI:** 10.1371/journal.pgen.1004160

**Published:** 2014-03-06

**Authors:** Tasnim Dayeh, Petr Volkov, Sofia Salö, Elin Hall, Emma Nilsson, Anders H. Olsson, Clare L. Kirkpatrick, Claes B. Wollheim, Lena Eliasson, Tina Rönn, Karl Bacos, Charlotte Ling

**Affiliations:** 1 Epigenetics and Diabetes, Department of Clinical Sciences, Lund University Diabetes Centre, Lund University, CRC, Malmö, Sweden; 2 Department of Cell Physiology and Metabolism, University Medical Center, Geneva, Switzerland; 3 Islet Cell Exocytosis, Department of Clinical Sciences, Lund University Diabetes Centre, Lund University, CRC, Malmö, Sweden; Albert Einstein College of Medicine, United States of America

## Abstract

Impaired insulin secretion is a hallmark of type 2 diabetes (T2D). Epigenetics may affect disease susceptibility. To describe the human methylome in pancreatic islets and determine the epigenetic basis of T2D, we analyzed DNA methylation of 479,927 CpG sites and the transcriptome in pancreatic islets from T2D and non-diabetic donors. We provide a detailed map of the global DNA methylation pattern in human islets, β- and α-cells. Genomic regions close to the transcription start site showed low degrees of methylation and regions further away from the transcription start site such as the gene body, 3′UTR and intergenic regions showed a higher degree of methylation. While CpG islands were hypomethylated, the surrounding 2 kb shores showed an intermediate degree of methylation, whereas regions further away (shelves and open sea) were hypermethylated in human islets, β- and α-cells. We identified 1,649 CpG sites and 853 genes, including *TCF7L2*, *FTO* and *KCNQ1*, with differential DNA methylation in T2D islets after correction for multiple testing. The majority of the differentially methylated CpG sites had an intermediate degree of methylation and were underrepresented in CpG islands (∼7%) and overrepresented in the open sea (∼60%). 102 of the differentially methylated genes, including *CDKN1A*, *PDE7B*, *SEPT9* and *EXOC3L2*, were differentially expressed in T2D islets. Methylation of *CDKN1A* and *PDE7B* promoters *in vitro* suppressed their transcriptional activity. Functional analyses demonstrated that identified candidate genes affect pancreatic β- and α-cells as Exoc3l silencing reduced exocytosis and overexpression of Cdkn1a, Pde7b and Sept9 perturbed insulin and glucagon secretion in clonal β- and α-cells, respectively. Together, our data can serve as a reference methylome in human islets. We provide new target genes with altered DNA methylation and expression in human T2D islets that contribute to perturbed insulin and glucagon secretion. These results highlight the importance of epigenetics in the pathogenesis of T2D.

## Author Summary

Epigenetic modifications such as DNA methylation are implicated in the development of human disease. However, genome-wide epigenetic analyses in patients with type 2 diabetes (T2D) remain scarce. In this study we aimed to unravel the epigenetic basis of T2D by analyzing DNA methylation of 479,927 CpG sites in human pancreatic islets from T2D and non-diabetic donors. We identified 1,649 CpG sites and 853 genes with differential DNA methylation (fold change 6–59%) in T2D islets. These include reported diabetes loci, such as *TCF7L2*, *FTO* and *KCNQ1*. Furthermore, we found 102 genes that showed both differential DNA methylation and gene expression in T2D islets, including *CDKN1A*, *PDE7B*, *SEPT9* and *EXOC3L2*. Finally, we provide functional proof that identified candidate genes directly affect insulin secretion and exocytosis in pancreatic β-cells as well as glucagon secretion in α-cells. Overall, this study provides a detailed map of the methylome in human pancreatic islets and demonstrates that altered DNA methylation in human islets contributes to perturbed hormone secretion and the pathogenesis of T2D.

## Introduction

Type 2 diabetes (T2D) is a complex multifactorial disorder characterized by chronic hyperglycemia due to impaired insulin secretion from pancreatic β-cells, elevated glucagon secretion from pancreatic α-cells and insulin resistance in target tissues. As a result of aging populations and an increasing prevalence of obesity and physical inactivity, the number of patients with T2D has dramatically increased worldwide [1]. Family studies together with genome-wide association studies (GWAS) have shown that the genetic background also influences the risk of T2D [2], [3]. The majority of T2D single nucleotide polymorphisms (SNPs) identified by GWAS are associated with impaired insulin secretion rather than insulin action, pointing to pancreatic islet defects as key mechanisms in the pathogenesis of T2D [3]–[5]. However, the identified SNPs only explain a small proportion of the estimated heritability of T2D, suggesting that additional genetic factors remain to be identified [3]. Genetic variants can interact with environmental factors and thereby modulate the risk for T2D through gene-environment interactions [6]. The interaction between genes and environment may also happen through direct chemical modifications of the genome by so called epigenetic modifications, including DNA methylation and histone modifications [7]. These are known to influence the chromatin structure and DNA accessibility and can thereby regulate gene expression [8], [9]. Epigenetic alterations may subsequently influence phenotype transmission and the development of different diseases, including T2D [7], [10]. Our group has recently found increased DNA methylation in parallel with decreased expression of *PPARGC1A*, *PDX-1* and *INS* in human pancreatic islets from patients with T2D by using a candidate gene approach [11]–[13]. Another group has analyzed DNA methylation of ∼0.1% of the CpG sites in the human genome in pancreatic islets from five T2D and 11 non-diabetic donors [14]. Animal studies further support the hypothesis that epigenetic modifications in pancreatic islets may lead to altered gene expression, impaired insulin secretion and subsequently diabetes [15]–[17]. Although these studies point towards a key role for epigenetic modifications in the growing incidence of T2D, comprehensive human epigenetic studies, covering most genes and regions in the genome in pancreatic islets from diabetic and non-diabetic donors, are still lacking. Human studies further need to link T2D associated epigenetic modifications with islet gene expression and eventually impaired insulin and/or glucagon secretion. Moreover, the human methylome has previously not been described in human pancreatic islets. In the present study, we analyzed the genome-wide DNA methylation pattern in pancreatic islets from patients with T2D and non-diabetic donors using the Infinium HumanMethylation450 BeadChip, which covers ∼480,000 CpG sites in 21,231 (99%) RefSeq genes. The degree of DNA methylation was further related to the transcriptome in the same set of islets. A number of genes that exhibited both differential DNA methylation and gene expression in human T2D islets were then selected for functional follow up studies; insulin and glucagon secretion were analyzed in clonal β- and α-cells, respectively where selected candidate genes had been either overexpressed or silenced. Also, reporter gene constructs were used to study the direct effect of DNA methylation on the transcriptional activity. Together, our study provides the first detailed map of the human methylome in pancreatic islets and it provides new target genes with altered DNA methylation and expression in human T2D islets that contribute to perturbed insulin and glucagon secretion.

## Results

### The methylome in human pancreatic islets

To describe the methylome in pancreatic islets and unravel the epigenetic basis of T2D, DNA methylation of a total of 485,577 sites were analyzed in human pancreatic islets from 15 T2D and 34 non-diabetic donors by using the Infinium HumanMethylation450 BeadChip. The characteristics of the islet donors included in the genome-wide analysis of DNA methylation are described in Table 1. T2D donors had higher HbA1c levels, nominally higher BMI and lower glucose-stimulated insulin secretion compared with non-diabetic donors (Table 1). There were no differences in islet purity (*P* = 0.97; Figure S1A) or β-cell content (*P* = 0.43; Figure S1B) between T2D and non-diabetic islets.

**Table 1:** Characteristics of human pancreatic islet donors included in the genome-wide analysis of DNA methylation in pancreatic islets.

	Non-diabetics (n = 34)	T2D (n = 15)	*P*-value
Gender (Males/Females)	(22/12)	(10/5)	
HbA1c (%)	5.4±0.4	6.9±1.0	1.2×10^−11^
Age (years)	56.0±9.0	59.5±10.7	0.2
BMI (kg/m^2^)	25.9±2.3	28.3±4.7	0.06
Glucose-stimulated insulin secretion (ng/islet·h) at 16.7 mM glucose	1.24±1.40	0.74±1.23	0.04

Mann-Whitney U test was used for statistical analysis and data are presented as mean ± SD.

A stringent quality control procedure was then performed and 2,546 (0.5%) sites were excluded for having a mean detection *P*-value>0.01 and as a result 483,031 sites generated reliable DNA methylation data and were used for further analysis. These 483,031 sites included 479,927 CpG sites, 3,039 non-CpG sites and 65 SNPs related to 21,231 RefSeq genes. Additional quality control steps were performed and all samples showed high bisulfite conversion efficiency (materials and methods).

**Figure 1:**
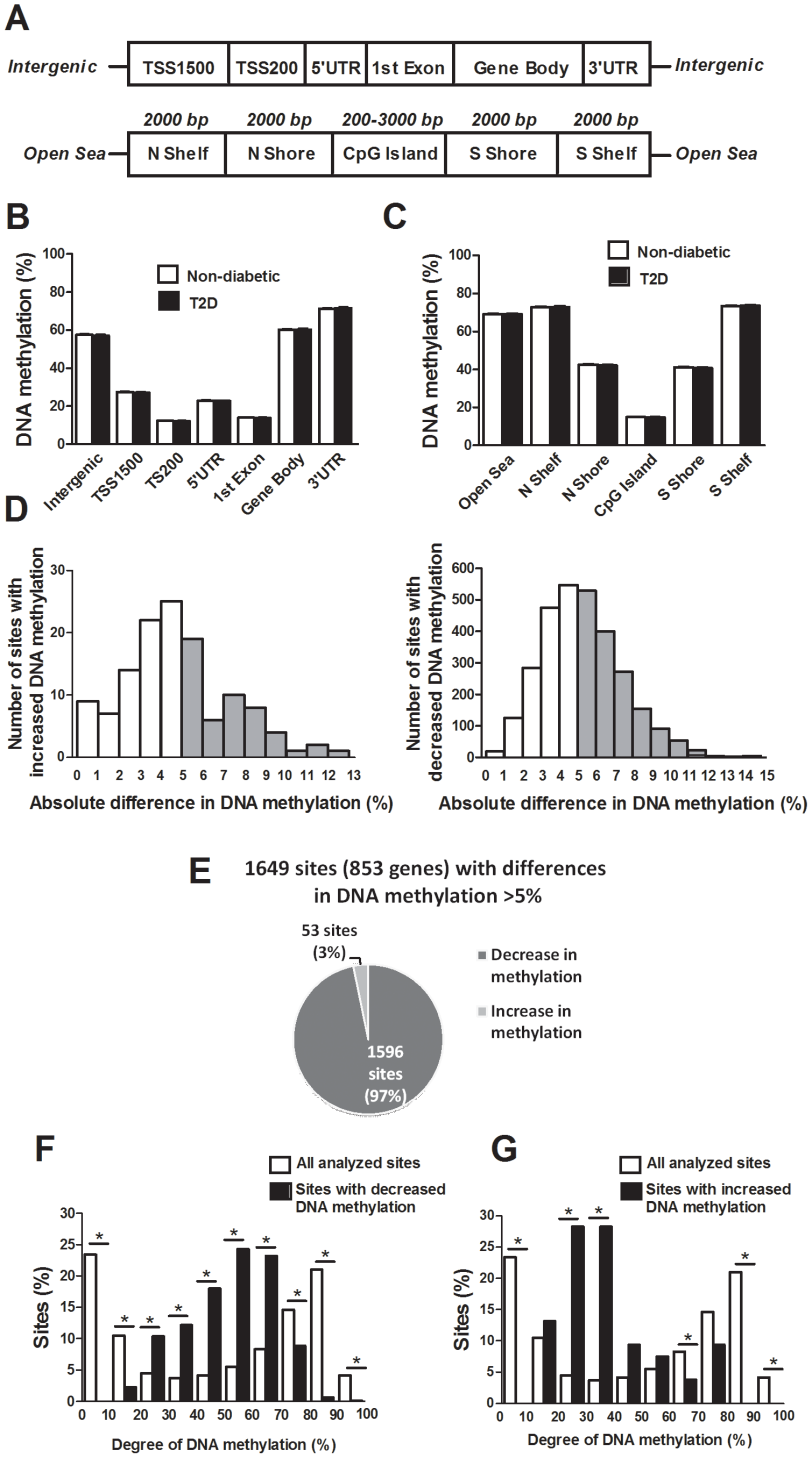
The human methylome in pancreatic islets from 15 T2D and 34 non-diabetic donors. (**A**) All analyzed DNA methylation sites on the Infinium HumanMethylation450 BeadChip are mapped to gene regions based on their functional genome distribution and CpG island regions based on CpG content and neighbourhood context [68]. TSS: proximal promoter, defined as 200 bp or 1500 bp upstream of the transcription start site. UTR: untranslated region. CpG island: 200 bp (or more) stretch of DNA with a C+G content of >50% and an observed CpG/expected CpG in excess of 0.6. Shore: the flanking region of CpG islands, 0–2000 bp. Shelf: regions flanking island shores, i.e., covering 2000–4000 bp distant from the CpG island [68]. Global DNA methylation in human pancreatic islets of T2D and non-diabetic donors is shown for (**B**) each gene region and (**C**) CpG island regions. Global DNA methylation is calculated as average DNA methylation based on all CpG sites in each annotated region on the chip. (**D**) The absolute difference in DNA methylation of 3,116 individual sites, including 2,988 sites with decreased and 128 sites with increased DNA methylation in T2D compared with non-diabetic human islets with *q*<0.05 based on a FDR analysis. 1,649 sites with an absolute difference in DNA methylation ≥5% are represented by grey bars. (**E**) Pie chart describing the number of sites that exhibit increased or decreased DNA methylation in T2D compared with non-diabetic human islets with an absolute difference in methylation ≥5% and *q*<0.05. The degree of DNA methylation is shown for (**F**) the 1,596 CpG sites with decreased DNA methylation and (**G**) the 53 sites with increased DNA methylation in T2D vs. non-diabetic islets in comparison to the degree of methylation of all analyzed CpG sites in the human islets using the Infinium HumanMethylation450 BeadChip.

The probes included on the Infinium HumanMethylation450 BeadChip have been annotated based on their relation to the nearest gene and the probes may belong to any of the following genomic elements: TSS1500, TSS200, 5′UTR, 1^st^ exon, gene body, 3′UTR or intergenic regions (Figure 1A). To describe the overall methylome in human pancreatic islets and to test whether there are global differences in DNA methylation in T2D islets, we calculated the average degree of DNA methylation of different genomic elements in T2D and non-diabetic islets. While genomic regions close to the transcription start site showed relatively low degrees of methylation (27.5±1.5% for TSS1500, 12.3±0.06% for TSS200, 23.0±1.2% for 5′UTR and 14.0±0.08% for 1^st^ exon in non-diabetic islets), there was a higher degree of methylation in regions further away from the transcription start site (60.2±1.9% for gene body, 71.3±1.8% for 3′UTR and 57.6±2.0% for intergenic regions) (Figure 1B). The probes on the Infinium HumanMethylation450 BeadChip have also been annotated based on their genomic location relative to CpG islands as shown in Figure 1A, where CpG island shores cover regions 0–2 kb from CpG islands and shelves cover regions 2–4 kb from CpG islands. North and south are used to determine whether the CpG site is upstream or downstream from a CpG island and open sea are isolated CpG sites in the genome. We found that CpG islands are hypomethylated (14.9±0.07%), shelves and open sea are hypermethylated (72.9±1.8% for N shelf, 73.5±1.8% for S shelf and 69.2±1.9% for open sea), while shores show an intermediate degree of methylation (42.5±2.1% for N shore and 41.1±2.0% for S shore) in human islets (Figure 1C). The average degree of DNA methylation for any of the genomic regions did not differ in T2D versus non-diabetic islets (Figure 1B–C).

### Differential DNA methylation in human islets from T2D versus non-diabetic donors

We next examined if any individual sites exhibit differential DNA methylation in pancreatic islets from T2D compared with non-diabetic donors. After correcting for multiple testing using a false discovery rate (FDR) analysis we identified 3,116 CpG sites that were differentially methylated between T2D and non-diabetic islets with FDR less than 5% (*q*<0.05), which means that 156 false positives are expected by chance [18]. The distribution of the absolute differences in DNA methylation between T2D and non-diabetic islets is shown in Figure 1D. To gain biological relevance, we filtered our DNA methylation results requiring absolute differences in methylation ≥5% between T2D and non-diabetic islets. We found 1,649 CpG sites, of which 1,008 are located in or near 853 unique genes and 561 are intergenic, that had absolute differences in methylation ≥5% in T2D versus non-diabetic islets and these were used for all further analyses (Figure 1D and Table S1). When the differences in DNA methylation between diabetic and non-diabetic islets of these 1,649 CpG sites are expressed as fold-change instead of absolute differences, we observe differences in methylation ranging from 6 to 59%. While 1,596 of these 1,649 CpG sites (97%) showed decreased DNA methylation, only 53 sites (3%) showed increased DNA methylation in T2D compared with non-diabetic islets (Figure 1E). The majority of CpG sites showing decreased DNA methylation in T2D versus non-diabetic islets had an intermediate degree of methylation and they were overrepresented among CpG sites with 20–70% methylation (Figure 1F). On the other hand, CpG sites showing increased DNA methylation in T2D versus non-diabetic islets had a lower degree of methylation and they were overrepresented among CpG sites with 20–40% methylation (Figure 1G). Our data suggest that CpG sites with an intermediate degree of DNA methylation are more dynamic to change in human islets.

### Chromosomal and genomic distribution of differentially methylated CpG sites in T2D islets

**Figure 2:**
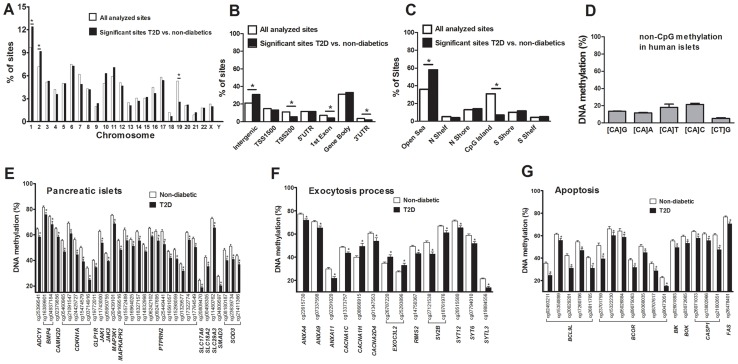
Distribution of individual sites that exhibit differential DNA methylation in human islets from 15 T2D versus 34 non-diabetic donors. (**A**) The chromosomal location of the 1,649 sites that exhibit differential DNA methylation in islets of T2D versus non-diabetic donors in comparison to all analyzed sites on the Infinium HumanMethylation450 BeadChip. (**B**) Distribution of significant CpG sites in relation to nearest gene regions. (**C**) Distribution of significant CpG sites in relation to CpG island regions. *The distribution of the significant sites compared with all analyzed sites on the Infinium HumanMethylation450 BeadChip is significantly different from what is expected by chance based on *Chi2* tests. *P*-values have been corrected for multiple testing using Bonferroni correction. (**D**) The average degree of DNA methylation of analyzed non-CpG sites in human pancreatic islets. Genes with previous known function in (**E**) pancreatic islets, (**F**) the exocytotic process and (**G**) apoptosis that exhibit differential DNA methylation in T2D versus non-diabetic human islets with an absolute difference in methylation ≥5%. * *q*<0.05. Data are mean ± SEM.

There is an accumulation of genetic variation on certain chromosomes associated with disease [19], [20]. However, it remains unknown if there is an over- or underrepresentation of differential DNA methylation on certain chromosomes linked to diabetes. We therefore determined the chromosomal distribution of the 1,649 sites that exhibit differential DNA methylation in human T2D islets (Figure 2A). Using *Chi2* tests, we found that the number of differentially methylated sites were overrepresented on chromosomes 1 and 2, and underrepresented on chromosome 19, in comparison to the chromosomal distribution of all analyzed sites on the Infinium HumanMethylation450 BeadChip. These results are not explained by the distribution of analyzed sites on the array or gene density on the chromosomes.

Previous cancer studies suggest that differential DNA methylation mainly occurs in CpG island shores rather than in CpG islands and promoter regions [21], [22]. However it remains unknown if this is also the case in T2D patients. We therefore evaluated the distribution of differentially methylated sites in T2D versus non-diabetic islets, either based on their relation to the nearest gene and functional genome distribution (Figure 2B) or based on the CpG content and neighbourhood content (Figure 2C). We found that differentially methylated CpG sites were overrepresented in intergenic regions and underrepresented in the TSS200, 1^st^ exon and 3′UTR in comparison to the probe distribution on the Infinium HumanMethylation450 BeadChip (Figure 2B). The distribution of the probes on the Infinium HumanMethylation450 BeadChip as well as the differentially methylated CpG sites in T2D islets in relation to a CpG island is further shown in Figure 2C. We found that ∼60% of the differentially methylated CpG sites in T2D islets are located in the open sea while ∼25% are located in the CpG island shores and only ∼7% are located in CpG islands (Figure 2C). Moreover, differentially methylated CpG sites were overrepresented in the open sea and underrepresented in CpG islands in comparison to the probe distribution on the Infinium HumanMethylation450 BeadChip (Figure 2C).

The Infinium HumanMethylation450 BeadChip covers 16,232 previously known differentially methylated regions (DMRs) that were selected based on the previously described criteria [21], [23]. We found 156 CpG sites with decreased and 4 CpG sites with increased DNA methylation in known DMRs in T2D compared with non-diabetic islets (Table S2), which is more than expected (*P*<0.001).

### Non-CpG methylation in human pancreatic islets

It has previously been established that DNA methylation in differentiated mammalian cells mainly occurs on cytosines in CG dinucleotides [24]. While the Infinium HumanMethylation450 BeadChip mainly analyzes DNA methylation in CpG sites, it also generated methylation data for 3,039 non-CpG sites in the human islets. However, only 1,189 of these non-CpG probes can be mapped with a perfect match to the correct genomic location annotated by Illumina [25] and methylation data of those probes were subsequently used for further analysis. These 1,189 sites were most predominant in the intergenic region, gene body and open sea (Figure. S2A–B). To test if non-CpG sites are methylated in human islets, we calculated the average degree of DNA methylation of the analyzed non-CpG sites. The average degree of methylation of non-CpG sites was 5.9–21.7% in human islets (Figure 2D and Table S3). Out of these non-CpG sites, only two sites were significant after correcting for multiple testing (*q*<0.05) and none of these sites had an absolute difference in methylation >5% in T2D versus non-diabetic islets.

### Biological features of the genes that exhibit differential methylation in T2D islets

We next performed a KEGG pathway analysis to identify biological pathways with enrichment of genes that exhibit differential DNA methylation in T2D versus non-diabetic islets. A total of 853 genes, represented by CpG sites with differential DNA methylation ≥5% in T2D islets (Table S1), were analyzed using WebGestalt. Relevant enriched KEGG pathways in T2D islets include pathways in cancer, axon guidance, MAPK signaling pathway, focal adhesion, ECM-receptor interaction and regulation of actin cytoskeleton (Table 2). We further performed a separate KEGG pathway analysis only including genes that exhibit increased DNA methylation in T2D islets and we then found an enrichment of genes in the complement and coagulation cascades; *C4A* and *C4B* (observed number of genes = 2, expected number of genes = 0.13 and *P*_adjusted_ = 0.0175). We also tested if any of the genes in Table 2 exhibit differential expression in human β- compared with α-cell fractions using published data by Dorrell *et al* [26]. However, among the genes included in Table 2, there were no significant differences in expression in β- versus α-cells.

**Table 2:** KEGG pathways with enrichment of genes that exhibit differential DNA methylation in pancreatic islets of 34 non-diabetic compared with 15 T2D donors.

Pathway (total number of genes in pathway)	Observed number of genes	Expected number of genes	Ratio observed/expected	Raw*P*-value	Adjusted*P*-value	Observed genes
Pathways in cancer (326)	38	13.14	2.89	4.5×10^−9^	5.1×10^−7^	*EP300, MAX, TRAF2, RXRA, EGF, CDKN1A, TGFB2, PDGFB, ETS1, TGFBR2, ABL1, CREBBP, ITGA3, AXIN2, COL4A1, WNT16, ARNT2, PTCH2, LAMA4, RARA, LAMC1, ZBTB16, RUNX1T1, PIK3R1, NTRK1, FGF12, RASSF5, VEGFA, FAS, SMAD3, LEF1, LAMC2, E2F2, BMP4, RUNX1, CCNA1, FGFR2, JAK1*
Axon guidance (129)	20	5.20	3.85	2.3×10^−7^	2.6×10^−5^	*RHOD, EPHA4, PLXNA2, NTN3, PLXNA1, PLXNB2, SEMA6A, SEMA5A, ABLIM2, SRGAP3, ABL1, ABLIM1, * ***SEMA4A*** *, SEMA4D, PAK2, * ***SEMA5B*** *, UNC5B, SEMA3A, EPHA8, FES*
MAPK signaling pathway (269)	27	10.84	2.49	1.3×10^−5^	0.0015	*FGFR4, MAX, TRAF2, NTRK1, MAP4K4, EGF, FGF12, * ***CACNA1H*** *, PRKX, TGFB2, PDGFB, CACNA2D3, CACNA2D4, TGFBR2, FLNB, FAS, MAPKAPK2, GNA12, CACNA1C, FGFR2, RASGRF2, PAK2, IL1R2, MAP3K5, PLA2G6, PLA2G4E, MAP3K1*
Focal adhesion (199)	21	8.02	2.62	5.7×10^−5^	0.0064	*LAMA4, PXN, LAMC1, PIK3R1, VAV2, EGF, VEGFA, PDGFB, ITGB5, DIAPH1, PPP1CC, FLNB, CAPN2, ZYX, SPP1, ITGA3, PAK2, VWF, COL4A1, * ***ITGB4*** *, LAMC2*
ECM-receptor interaction (84)	12	3.38	3.55	0.0001	0.011	*LAMA4, SPP1, SV2B, LAMC1, GP5, * ***AGRN*** *, ITGA3, VWF, COL4A1, * ***ITGB4*** *, ITGB5, LAMC2*
Regulation of actin cytoskeleton (211)	20	8.50	2.35	0.0004	0.045	*FGFR4, PXN, SSH1, PIK3R1, VAV2, EGF, CHRM1, FGF12, GIT1, PDGFB, ITGB5, DIAPH1, PPP1CC, BAIAP2, GNA12, ITGA3, FGFR2, INSRR, PAK2, * ***ITGB4***

Hypermethylated genes in T2D islets are in bold, *P*-values have been adjusted for multiple testing using the Benjamini-Hochberg method.

Previous GWAS have identified SNPs associated with T2D and/or obesity [3]. These SNPs have been linked to candidate genes, representing genes closest to respective risk SNPs. However, the SNPs identified in GWAS only explain a small proportion of the estimated heritability for T2D, proposing that there are additional genetic factors left to be discovered. These may include genetic factors interacting with epigenetics [27]. We therefore tested if any of 40 T2D candidate genes and 53 obesity genes identified by GWAS were differentially methylated in the human T2D islets [3]. The Infinium HumanMethylation450 BeadChip covers 1,525 CpG sites representing 39 of the T2D candidate genes and 1,473 CpG sites representing all 53 obesity genes. However, one should keep in mind that for a number of these SNPs it still remains unknown if the closest gene is the gene involved in T2D or obesity and if the identified SNP is the functional SNP. Therefore, to cover most regions harboring a genetic variant associated with T2D or obesity, we also investigated the level of DNA methylation for all CpG sites in a region 10 kb up- and downstream of intergenic SNPs associated with T2D (n = 28) and obesity (n = 41) (www.genome.gov/gwastudies. Accessed: March 18, 2013). We identified 44 methylation sites, representing 17 T2D candidate genes and one intergenic SNP that were differentially methylated in T2D versus non-diabetic islets with a FDR less than 5% (*q*<0.05, Table 3). Twenty-one of these sites, representing ten genes, had absolute differences in methylation >5% in T2D versus non-diabetic islets, which correspond to a fold change ranging from 7 to 28%. Only three sites in three different obesity genes were differentially methylated in T2D islets and one of these sites had an absolute difference in methylation >5% (Table 3).

**Table 3:** Candidate genes and intergenic SNPs for T2D and obesity that exhibit differential DNA methylation in human pancreatic islets of 34 non-diabetic compared with 15 T2D donors.

Gene symbol	Probe ID	Non-diabetic DNA methylation (%)	T2D DNA methylation (%)	Delta DNA methylation (%)	*P*-value	*q*-value	Chr.	Position	Gene region	GWAS trait
***ADAMTS9***	**cg25859972**	**41.19±5.74**	**34.53±6.83**	**−6.66**	**1.2×10^−3^**	**0.047**	**3**	**64645555**	**Body**	**T2D**
*ADCY5*	cg27182923	61.63±6.82	56.96±5.94	−4.67	5.3×10^−4^	0.037	3	124612077	Body	T2D
***ADCY5***	**cg01246398**	**58.41±6.73**	**52.33±6.35**	**−6.08**	**5.6×10^−4^**	**0.037**	**3**	**124648562**	**Body**	**T2D**
***ADCY5***	**cg25976932**	**61.85±7.04**	**55.45±7.33**	**−6.40**	**6.6×10^−4^**	**0.037**	**3**	**124621648**	**Body**	**T2D**
***ADCY5***	**cg02953559**	**71.50±6.41**	**64.82±6.40**	**−6.68**	**8.6×10^−4^**	**0.041**	**3**	**124647654**	**Body**	**T2D**
***ADCY5***	**cg15464481**	**62.27±9.16**	**53.27±8.45**	**−9.01**	**3.5×10^−4^**	**0.037**	**3**	**124634652**	**Body**	**T2D**
*BCL11A*	cg03606511	69.40±5.07	64.87±5.81	−4.52	1.4×10^−3^	0.049	2	60589034	Body	T2D
*CDKAL1*	cg12556823	85.75±2.64	85.30±3.64	−0.45	7.3×10^−4^	0.039	6	21131464	Body	T2D
*DUSP8*	cg14488974	71.10±3.93	67.35±3.70	−3.75	4.1×10^−5^	0.016	11	1536002	Body	T2D
***FTO***	**cg26982104**	**29.73±5.52**	**23.13±4.72**	**−6.60**	**3.6×10^−4^**	**0.037**	**16**	**52336836**	**Body**	**T2D and obesity**
***FTO***	**cg01485549**	**64.84±9.62**	**56.14±10.53**	**−8.70**	**5.2×10^−4^**	**0.037**	**16**	**52346986**	**Body**	**T2D and obesity**
*HHEX*	cg20180364	61.64±4.26	58.49±4.91	−3.15	1.4×10^−3^	0.049	10	94438512	TSS1500	T2D
*HHEX*	cg09721427	30.62±4.09	27.26±3.75	−3.36	7.2×10^−4^	0.039	10	94438682	TSS1500	T2D
*HHEX*	cg16508068	55.98±5.16	51.97±4.77	−4.01	8.5×10^−4^	0.041	10	94441716	Body	T2D
***HHEX***	**cg26979504**	**44.33±4.65**	**38.62±5.44**	**−5.71**	**2.6×10^−4^**	**0.033**	**10**	**94441498**	**Body**	**T2D**
*HMGA2*	cg03257822	10.29±1.49	9.09±0.92	−1.20	5.1×10^−4^	0.037	12	64503074	TSS1500;Body	T2D
***HNF1B***	**cg21250756**	**28.57±5.12**	**20.71±3.83**	**−7.86**	**5.2×10^−6^**	**0.004**	**17**	**33151413**	**Body**	**T2D**
*IGF2BP2*	cg21531679	66.37±3.80	64.27±4.44	−2.10	1.4×10^−3^	0.049	3	186937720	Body	T2D
***IRS1***	**cg04751089**	**40.02±6.28**	**33.09±4.40**	**−6.93**	**5.5×10^−5^**	**0.016**	**2**	**227367880**	**3′UTR**	**T2D**
***IRS1***	**cg05263838**	**41.54±8.39**	**34.27±4.43**	**−7.27**	**1.1×10^−3^**	**0.047**	**2**	**227367650**	**3′UTR**	**T2D**
*JAZF1*	cg23597162	80.20±3.06	77.41±4.33	−2.79	6.1×10^−5^	0.016	7	28068866	Body	T2D
*JAZF1*	cg14491535	72.77±4.13	68.59±3.00	−4.18	5.0×10^−4^	0.037	7	28161615	Body	T2D
***JAZF1***	**cg11526778**	**57.89±5.31**	**52.87±5.74**	**−5.02**	**6.0×10^−4^**	**0.037**	**7**	**28082308**	**Body**	**T2D**
*KCNQ1*	cg08895013	72.11±3.47	68.49±3.95	−3.62	1.2×10^−4^	0.022	11	2424908	Body	T2D
*KCNQ1*	cg20533553	51.83±4.37	47.76±4.90	−4.08	6.3×10^−4^	0.037	11	2421398	TSS1500	T2D
*KCNQ1*	cg26896748	87.66±4.31	83.55±6.85	−4.11	5.5×10^−4^	0.037	11	2817640	Body	T2D
*KCNQ1*	cg17305275	81.71±2.92	77.27±4.61	−4.44	1.1×10^−3^	0.047	11	2703251	Body	T2D
*KCNQ1*	cg03371125	53.56±5.40	48.66±5.26	−4.89	6.2×10^−4^	0.037	11	2421421	TSS1500	T2D
***KCNQ1***	**cg03600094**	**69.15±6.30**	**63.30±5.93**	**−5.85**	**1.5×10^−4^**	**0.023**	**11**	**2438051**	**TSS1500;Body**	**T2D**
***KCNQ1***	**cg01693193**	**72.51±5.35**	**66.36±5.69**	**−6.15**	**4.6×10^−5^**	**0.016**	**11**	**2661729**	**Body**	**T2D**
***KCNQ1***	**cg03170016**	**65.77±8.65**	**56.41±8.93**	**−9.36**	**2.6×10^−4^**	**0.033**	**11**	**2438718**	**TSS1500;Body**	**T2D**
*PPARG*	cg04908300	37.30±3.34	34.33±3.41	−2.98	1.3×10^−4^	0.022	3	12305532	5′UTR;1stExon	T2D
*PPARG*	cg10499651	64.69±7.28	59.92±7.95	−4.77	1.4×10^−3^	0.049	3	12440415	Body	T2D
*RBMS1*	cg01757209	4.43±0.61	4.18±0.63	−0.26	1.3×10^−3^	0.047	2	160972619	Body	T2D
*TCF7L2*	cg26380291	64.60±4.36	60.94±4.43	−3.67	9.8×10^−4^	0.045	10	114777833	Body	T2D
***TCF7L2***	**cg07591090**	**78.39±5.70**	**72.44±5.97**	**−5.95**	**1.1×10^−3^**	**0.047**	**10**	**114867606**	**Body**	**T2D**
***TCF7L2***	**cg06403317**	**72.53±6.12**	**65.26±7.39**	**−7.26**	**1.5×10^−3^**	**0.049**	**10**	**114867642**	**Body**	**T2D**
***TCF7L2***	**cg03384318**	**78.28±6.67**	**70.43±7.19**	**−7.85**	**4.4×10^−4^**	**0.037**	**10**	**114867679**	**Body**	**T2D**
***TCF7L2***	**cg03510732**	**56.92±8.96**	**47.90±8.59**	**−9.02**	**1.3×10^−3^**	**0.047**	**10**	**114861921**	**Body**	**T2D**
*THADA*	cg22076676	41.57±3.33	38.39±3.06	−3.18	1.2×10^−3^	0.047	2	43357439	Body	T2D
*THADA*	cg24168549	80.90±4.90	75.99±4.62	−4.91	1.3×10^−4^	0.022	2	43507634	Body	T2D
***THADA***	**cg19211851**	**45.34±6.67**	**39.76±6.96**	**−5.58**	**7.9×10^−4^**	**0.040**	**2**	**43408970**	**Body**	**T2D**
***THADA***	**cg01649611**	**70.96±4.93**	**63.16±5.91**	**−7.81**	**5.4×10^−8^**	**0.003**	**2**	**43374570**	**Body**	**T2D**
*rs5015480*	cg20134984	77.88±3.57	74.62±5.58	−3.26	6.0×10^−4^	0.037	10	94446626	intergenic	T2D
*FAIM2*	cg04920032	19.84±3.59	15.68±3.01	−4.16	1.4×10^−5^	0.010	12	48549253	3′UTR	Obesity
*NPC1*	cg21243631	61.24±4.09	56.37±4.38	−4.87	1.0×10^−6^	0.001	18	19408258	Body	Obesity
***VEGFA***	**cg01298514**	**80.65±3.69**	**74.63±4.99**	**−5.72**	**3.7×10^−5^**	**0.018**	**6**	**43844785**	**TSS1500**	**Obesity**

Probes shown in bold are CpG sites with an absolute difference in DNA methylation >5% between non-diabetic and T2D human islets. *q*-values are based on a FDR analysis.

Finally, based on literature search, genes with known functions in pancreatic islets and/or β-cells [28]–[40] (Figure 2E), the exocytotic process [41]–[44] (Figure 2F) and apoptosis [45] (Figure 2G) were found among the genes that showed differential DNA methylation in T2D islets.

### Differential mRNA expression and differential methylation in T2D islets

**Figure 3:**
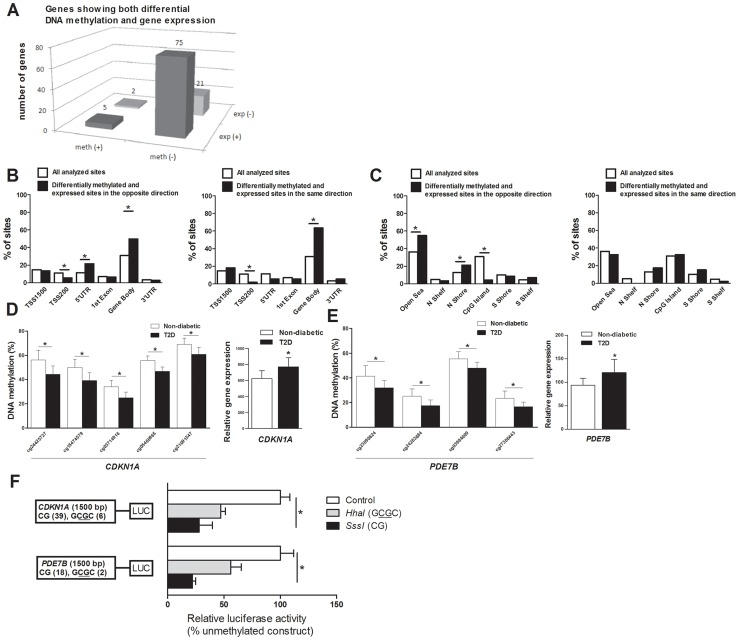
Relation between DNA methylation and gene expression in human pancreatic islets. (**A**) The number of genes that exhibit both differential DNA methylation and gene expression in pancreatic islets from 15 T2D versus 34 non-diabetic donors. One gene (*SLC44A4*) was counted twice in the figure because it showed increased expression in T2D islets and was associated with probes that showed both increased and decreased DNA methylation. Distribution of differentially methylated CpG sites located in/near genes that also exhibit differential expression in T2D islets with an inverse relationship or a positive relationship in relation to their (**B**) functional genome distribution and in relation to (**C**) CpG island regions. Decreased DNA methylation and increased mRNA expression of (**D**) *CDKN1A* and (**E**) *PDE7B* in pancreatic islets of T2D versus non-diabetic donors. (**F**) A diagram of the two luciferase reporter plasmids used to test the effect of DNA methylation on *CDKN1A* and *PDE7B* promoter activity is shown. The two plasmids contain 1500 bp of either the *CDKN1A* or the *PDE7B* promoter regions inserted into a pCpGL-basic vector. Methylated (grey and black bars) or mock-methylated (white bars) promoter constructs were transfected into clonal β-cells for 48 hours prior to luciferase assay. The data were normalized with co-transfected renilla luciferase control vector and are the average of three separate experiments with five replicates each. In each experiment, cells were transfected with an empty pCpGL-basic vector as a background control. * *P*<0.05. Data are mean ± SEM.

DNA methylation of certain genomic regions may silence gene transcription [8], [12]. We therefore used microarray mRNA expression data to examine if any of the 853 genes that exhibit differential DNA methylation in T2D islets also exhibit differential mRNA expression in islets from the same donors. We found that 102 of the 853 differentially methylated genes were also differentially expressed in T2D compared with non-diabetic islets (Table S4). While 77 (∼75%) of the differentially expressed genes had an inverse relationship with DNA methylation, e.g. decreased DNA methylation was associated with increased gene expression in T2D islets, 26 (∼25%) had a positive relationship with DNA methylation, e.g. decreased DNA methylation was associated with decreased expression (Figure 3A and Table S4). Figure 3B–C describes the genomic distribution of the differentially methylated CpG sites that are located in/near genes that also exhibit differential expression in T2D islets. Interestingly, there was an overrepresentation of CpG sites in the 5′UTR only when differential DNA methylation and gene expression show an inverse relationship (Figure 3B). In addition, CpG sites in the open sea and northern shore were overrepresented while sites in the CpG islands were underrepresented when DNA methylation and gene expression show an inverse relationship (Figure 3C). These data suggest that differential DNA methylation in certain genomic regions may contribute to an inverse regulation of gene expression. In addition, we found an overrepresentation of differentially methylated CpG sites in the gene body regardless of whether methylation and gene expression show a positive or inverse relationship (Figure 3B), this is known as the DNA methylation paradox which still remains unexplained [46].

### Functional analysis of differentially methylated genes

We continued to functionally test if DNA methylation affects gene expression. We selected two genes with differential DNA methylation of multiple CpG sites and in which DNA methylation showed an inverse relationship with gene expression for luciferase experiments (Table S4 and Figure 3D–E). Reporter gene constructs were subsequently produced for *CDKN1A* and *PDE7B*. Promoter sequences of the selected genes were inserted into a luciferase expression plasmid that completely lacks CpG dinucleotides. The constructs could thereby be used to study the effect of DNA methylation on luciferase activity in transfection assays. Each construct was mock methylated or methylated with the methyltransferases *SssI* or *HhaI* that methylates all CpG sites or the internal cytosine residue in a GCGC sequence only, respectively. *SssI* methylation thereby results in highly methylated constructs and *HhaI* methylation gives point methylated constructs in which only a fraction of the CpG sites are methylated. The number of CpG sites that may be methylated by these enzymes in each respective construct is shown in Figure 3F. Clonal β-cells were then transfected with the mock-methylated or methylated constructs. The highest reporter gene expression was generated by the mock-methylated control constructs including the promoter regions (Figure 3F). Furthermore, methylation of the human *CDKN1A* and *PDE7B* promoter regions suppressed reporter expression significantly (*P*<0.05). While methylation of the promoter regions by *SssI* suppressed reporter gene expression to 28.0±11.8% for *CDKN1A* and to 22.1±2.9% for *PDE7B*, point methylation by *HhaI* suppressed reporter expression to 47.2±7.1% for *CDKN1A* and to 55.9±9.4 for *PDE7B* (Figure 3F).

### Overexpression of Cdkn1a, Pde7b and Sept9 in clonal β-cells results in impaired insulin secretion

**Figure 4:**
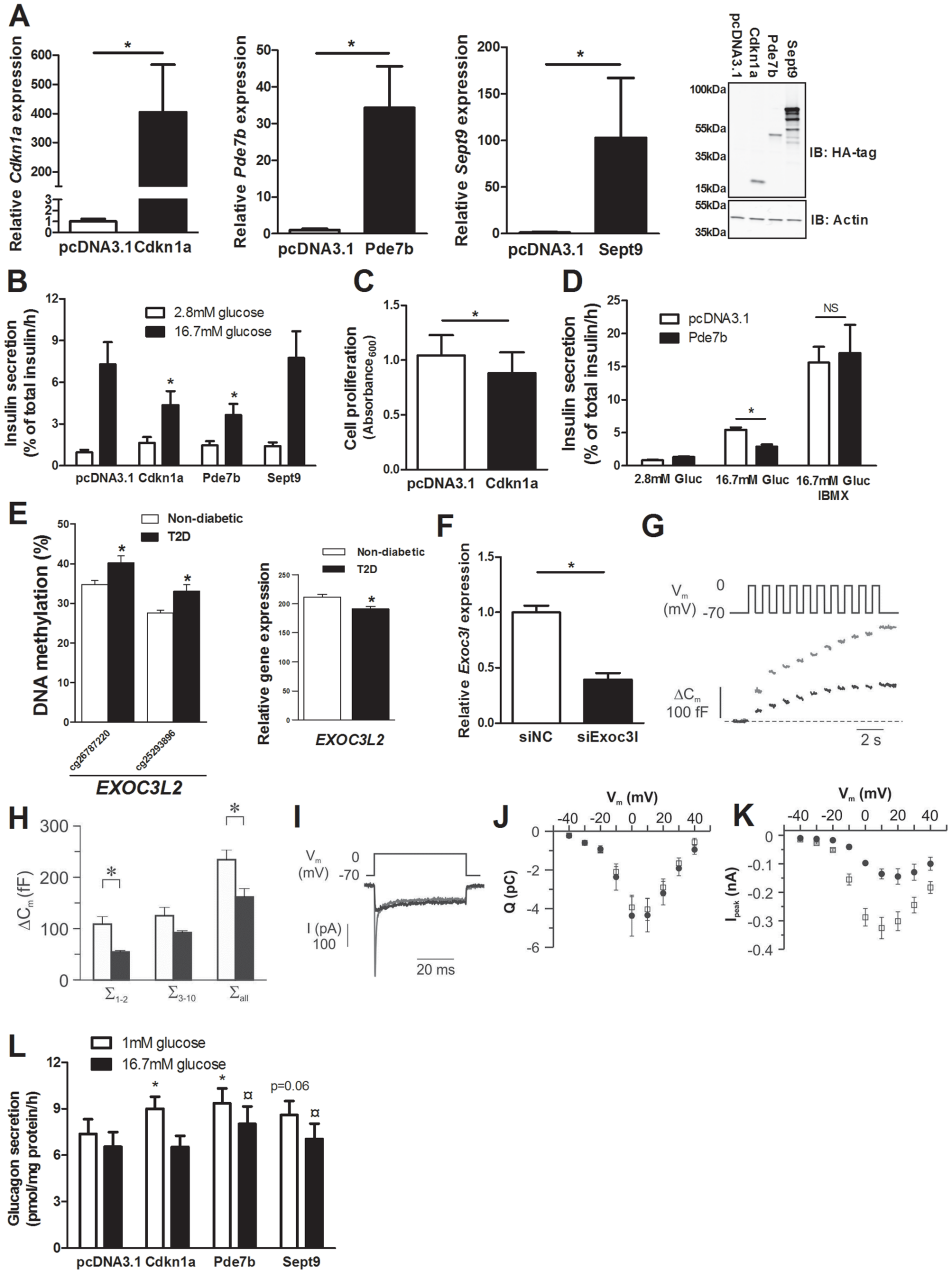
Impact of Cdkn1a, Pde7b, Sept9 and Exoc3l on clonal β- and α-cells. Glucose-sensitive clonal INS-1 832/13 β-cells were used to study the impact of Cdkn1a, Pde7b, Sept9 and Exoc3l on insulin secretion and β-cell function. (**A**) Overexpression of *Cdkn1a*, *Pde7b* and *Sept9* with pcDNA3.1 expression vectors in clonal β-cells resulted in elevated mRNA levels (black bars) compared with β-cells transfected with an empty pcDNA3.1 vector (white bars). * *P*<0.05. Data are mean ± SEM. Overexpression was also evident at the protein level as determined by immunoblot detection of the HA-tag situated on the c-terminal of the cloned cDNAs (rightmost panel) (**B**) Insulin secretion in response to 2.8 mM (white bars) and 16.7 mM (black bars) glucose in clonal β-cells overexpressing either Cdkn1a, Pde7b or Sept9 compared with control cells transfected with an empty pcDNA3.1 vector (n = 5). * *P*<0.05. Data are mean ± SEM (**C**) Decreased cell proliferation in clonal β-cells overexpressing Cdkn1a (black bar) compared with cells transfected with an empty pcDNA3.1 vector (white bar) (n = 4). * *P*<0.05. Data are mean ± SEM (**D**) Insulin secretion in response to 2.8 mM and 16.7 mM glucose (Gluc) or 16.7 mM glucose (Gluc) in combination with 100 µM IBMX in clonal β-cells overexpressing Pde7b (black bars) compared with cells transfected with an empty pcDNA3.1 vector (white bars) (n = 3). * *P*<0.05. NS = not significant. (**E**) Increased DNA methylation and decreased mRNA expression of *EXOC3L2* in pancreatic islets of 15 T2D versus 34 non-diabetic donors. (**F**) Transfection of clonal β-cells with siRNA targeting *Exoc3l* resulted in decreased *Exoc3l* mRNA expression (siExo3l, black bar) when compared with clonal β-cells transfected with negative control siRNA (siNC, white bar) (n = 3). * *P*<0.05. Silencing of Exoc3l resulted in decreased Ca^2+^-dependent exocytosis; (**G**) Depolarizationevoked a decrease in membrane capacitance (ΔC_m_) in single INS1-832/13 β-cells where Exoc3l was silenced (black trace) compared with control β-cells (grey trace) treated with negative control siRNA. (**H**) Histogram of the summed increase in membrane capacitance evoked by the two first depolarizations (Σ_1–2_), the latter eight depolarizations (Σ_3–10_) or all depolarizations in the train (Σ_all_). Data are mean ± SEM of n = 11 β-cells treated with control siRNA (white bars) and n = 4 β-cells treated with siRNA against *Exoc3l* (black bars). * *P*<0.05. (**I**) Depolarization-evoked increase in current (I) in single INS1-832/13 β-cells treated with control siRNA (grey trace) or siRNA targeting *Exoc3l* (black trace). Notice that the rapid Na^+^-current is markedly diminished in siExoc3l treated clonal β-cells. (**J**) Reduced expression of Exoc3l has no effect on the Ca^2+^ influx. Charge (Q)-voltage (V_m_) relationship in β-cells treated with control (white squares) siRNA and siRNA against *Exoc3l* (black circles). The charge is representative of the Ca^2+^-influx into the cell through the voltage-dependent Ca^2+^ channels. (**K**) As in **J**, but the peak-current (Ipeak)-voltage (Vm)-relationship was estimated as a representation of the voltage-dependent Na^+^ current. The Na^+^ current is significantly reduced in siExoc3l cells (*P*<0.05) versus control siRNA cells except for the depolarization to −40 mV. Data in **J** and **K** are mean ± SEM of n = 11 β-cells treated with control siRNA and n = 7 siExoc3l treated β-cells. (**L**) αTC1-6 cells were used to determine the impact of Cdkn1a, Pde7b and Sept9 on glucagon secretion. Overexpression of Cdkn1a and Pde7b resulted in significantly increased glucagon secretion at 1 mM glucose (white bars) (n = 4, * *P*<0.05), while overexpression of Pde7b and Sept9 resulted in increased glucagon secretion at 16.7 mM glucose (black bars) (n = 4, ¤ *P*<0.05) compared with control cells transfected with an empty pcDNA3.1 vector.

We identified 75 genes that exhibit decreased DNA methylation and increased gene expression in pancreatic islets of T2D compared with non-diabetic donors when performing the genome-wide DNA methylation analysis (Table S4). To model the situation in humans and elucidate the mechanisms whereby these genes may contribute to impaired β-cell function and the development of T2D, we overexpressed three of these genes; *Cdkn1a*, *Pde7b* and *Sept9*, in clonal β-cells (Figure 4A). These genes were selected based on their potential role in diabetes and islet function and because they showed both differential DNA methylation of multiple CpG sites and differential gene expression in T2D islets. Overexpression of Cdkn1a and Pde7b led to a significant decrease in glucose-stimulated insulin secretion in clonal β-cells, while Sept9 had no significant effect (Figure 4B and Figure S3A). Moreover, while the direct response to the membrane-depolarizing agent KCl was unaffected, the fold-change of insulin secretion at KCl-stimulation divided by insulin secretion at low glucose was decreased in clonal β-cells overexpressing Cdkn1a, Pde7b or Sept9 (Figure S3B–C). *Cdkn1a* (also known as p21) encodes a potent cyclin-dependent kinase inhibitor that regulates cell cycle progression [31] and we therefore tested if overexpression of this gene would affect cell proliferation in clonal β-cells. Indeed increased Cdkn1a levels resulted in decreased β-cell proliferation (Figure 4C). *Pde7b* encodes a cAMP-specific phosphodiesterase [47] and cAMP potentiates insulin secretion [48]. We next stimulated Pde7b overexpressing cells with glucose in combination with IBMX, a general phosphodiesterase inhibitor. Addition of IBMX normalized glucose-stimulated insulin secretion in Pde7b overexpressing β-cells (Figure 4D) suggesting that the cAMP hydrolyzing activity of Pde7b underlies the secretory defect.

### Silencing of *Exoc3l* in clonal β-cells results in decreased exocytosis

*EXOC3L2* is one of the genes that exhibit increased DNA methylation and decreased gene expression in pancreatic islets of T2D compared with non-diabetic donors (Figure 4E and Table S4). The protein encoded by *EXOC3L2* is part of the exocyst complex [42] and may consequently affect exocytosis of insulin from pancreatic β-cells. To model the situation in human T2D islets, and to examine if decreased levels of EXOC3L2 affect β-cell exocytosis, *Exoc3l* was silenced in clonal β-cells using siRNA. This resulted in a 60.7% reduction of *Exoc3l* levels (Figure 4F). Next, exocytosis was measured as changes in membrane capacitance using the patch-clamp technique. Silencing of *Exoc3l* resulted in decreased β-cell exocytosis (Figure 4G–H). In particular, the two first depolarizations, representing the rapid first-phase insulin secretion, were decreased in Exoc3l deficient β-cells (Figure 4H). Moreover, the Ca^2+^ current was unaffected in Exoc3l deficient β-cells (Figure 4I–J), demonstrating a direct effect of Exoc3l on the exocytosis machinery rather than on the Ca^2+^ current. We also observed decreased voltage dependent Na^+^ current in Exoc3l deficient β-cells (Figure 4I, K).

### Overexpression of Cdkn1a, Pde7b and Sept9 in clonal α-cells results in increased glucagon secretion

As elevated glucagon levels in the fasted state contribute to hyperglycemia in patients with T2D [49], we next tested if selected candidate genes that exhibit decreased DNA methylation and increased expression in pancreatic islets of T2D compared with non-diabetic donors contribute to increased glucagon secretion in pancreatic α-cells (αTC1-6 cells). While overexpression of Cdkn1a and Pde7b resulted in increased glucagon release at 1 mM glucose compared to control transfected α-cells, Sept9 overexpression led to a borderline significant increase in glucagon secretion (Figure S3D and Figure 4L). Additionally, when stimulated with 16.7 mM glucose, Pde7b and Sept9 overexpressing α-cells secreted more glucagon than control transfected cells (Figure 4L).

### Technical validation and replication of DNA methylation data in T2D islets

As a technical replication of the Infinium HumanMethylation450 BeadChip data, one islet sample was bisulfite converted and analyzed on the Infinium chips at two different occasions. The correlation for DNA methylation of 100 000 randomly chosen CpG sites on the two chips was then calculated and the methylation data showed a strong positive correlation (R^2^ = 0.99, *P* = 2.2×10^−16^; Figure S4A).

In addition, seven of the CpG sites that exhibit differential DNA methylation in T2D islets were selected for technical validation of the Infinium HumanMethylation450 BeadChip data with pyrosequencing. The CpG sites selected for technical validation include cg21091547 (*CDKN1A*), cg27306443 (*PDE7B*), cg19654743 (*SEPT9*), cg04751089 (*IRS1*), cg20995304 (*HDAC7*), cg01649611 (*THADA*) and cg15572489 (*PTPRN2*) (Table 4). In agreement with the Infinium HumanMethylation450 BeadChip data, all seven CpG sites showed differential DNA methylation in T2D versus non-diabetic islets when analyzed with pyrosequencing with differences in methylation of a similar magnitude between the two groups (Table 4). Furthermore, the DNA methylation data generated with Infinium HumanMethylation450 BeadChip and pyrosequencing for these seven CpG sites correlated strongly (rho = 0.84–0.94, *P*≤1.2×10^−13^; Table 4 and Figure S4B).

**Table 4:** Technical validation of Infinium HumanMethylation450 BeadChip data using pyrosequencing.

Infinium HumanMethylation450 BeadChip data							Pyrosequencing data				Correlation between the two methods	
Gene symbol	Probe ID	Non-diabetic DNA meth (%)	T2D DNA meth (%)	Delta DNA meth (%)	*P*-value	*q*-value	Non-diabetic DNA meth (%)	T2D DNA meth (%)	Delta DNA meth (%)	*P*-value	*rho*	*P*-value
*CDKN1A*	cg21091547	69.2±5.1	61.0±5.7	−8.2	3.7×10^−6^	0.0091	75.8±6.2	66.2±8.2	−9.5	1.3×10^−3^	0.84	1.2×10^−13^
*PDE7B*	cg27306443	23.3±6.0	16.5±3.8	−6.8	2.1×10^−6^	0.0076	12.5±4.2	8.5±2.8	−4.0	5.1×10^−3^	0.89	6.3×10^−17^
*SEPT9*	cg19654743	65.9±7.1	58.0±7.1	−7.8	2.5×10^−5^	0.018	84.2±6.1	76.2±9.5	−8.0	6.0×10^−3^	0.93	1.8×10^−21^
*IRS1*	cg04751089	40.0±6.3	33.1±4.4	−6.9	5.5×10^−5^	0.025	39.9±8.3	31.3±5.3	−8.6	1.3×10^−5^	0.90	2.8×10^−18^
*HDAC7*	cg20995304	77.5±5.5	71.1±7.0	−6.4	2.8×10^−4^	0.048	80.3±6.4	71.4±9.3	−8.9	5.5×10^−3^	0.94	2.1×10^−22^
*THADA*	cg01649611	71.0±4.9	63.2±5.9	−7.8	5.4×10^−8^	0.0031	77.2±8.6	69.1±9.6	−8.1	8.6×10^−3^	0.85	4.1×10^−14^
*PTPRN2*	cg15572489	64.0±8.1	55.5±7.5	−8.4	2.5×10^−5^	0.018	63.7±7.8	53.9±9.7	−9.7	4.1×10^−3^	0.90	4.2×10^−17^

*q*-values are based on a FDR analysis. Correlations between Infinium HumanMethylation450 BeadChip data and pyrosequencing data were analyzed using Spearman's test. *Rho* represents the correlation coefficient.

### Confirmation of DNA methylation data in human T2D islets

To further validate our results, we tested if any of the CpG sites that exhibit differential DNA methylation in T2D islets of a recent study by Volkmar *et al* [14], also exhibit differential methylation in T2D islets in our study. While our study analyzed DNA methylation of 479,927 CpG sites distributed across the entire genome and 99% of RefSeq genes, the study by Volkmar et al. only analyzed 27,578 CpG sites distributed mainly in CpG islands in a subset of RefSeq genes and it was therefore only possible to compare some of our studied CpG sites. Nevertheless, our array covers 264 of the 276 CpG sites that exhibit differential DNA methylation in the study by Volkmar *et al* and 71 of these sites (∼27%) were differentially methylated in our study with *P*<0.05, which is more than expected by chance (*Chi2* = 66.7 and *P*<0.0001; Table S5). The data by Volkmar *et al* have not been corrected for multiple testing and it may therefore include false positive results, which may explain the difficulty to replicate some of their results. Yet, the DNA methylation data of the 264 CpG sites analyzed in both studies correlated significantly for both non-diabetic (rho = 0.66, *P*<0.0001; Figure S5A) and diabetic (rho = 0.68, *P*<0.0001; Figure S5B) islets.

As an additional control, we compared the results from the present study with our previous studies where we found differential DNA methylation of *PPARGC1A*, *INS* and *PDX1* in human pancreatic islets of T2D versus non-diabetic donors by using a candidate gene approach [11]–[13]. For *PPARGC1A* we were unable to compare the methylation data between the two studies as our previous study was based on the average degree of methylation of multiple CpG sites in an analyzed genomic region [11]. We have previously found increased DNA methylation in four CpG sites of the *INS* gene in human pancreatic islets from T2D compared with non-diabetic donors [13]. Two of these four CpG sites (+63 and −180) were covered by probes on the Infinium HumanMethylation450 BeadChip and in agreement with the results in our previous study these two CpG sites show increased DNA methylation in T2D versus non-diabetic islets also in the present analysis (cg00613255, *P* = 0.008 and cg25336198, *P* = 0.003, Figure S5C). In the present study there were 13 additional CpG sites annotated to the *INS* gene with increased DNA methylation in T2D islets and *P*<0.05 (Figure S5C). On the other hand, none of the individual CpG sites showing differential methylation in *PDX1* in T2D islets in our previous study [12] were covered by probes on the Infinium HumanMethylation450 BeadChip. However, using EpiTYPER, we could validate our previous *PDX1* data [12], in the islet samples included in the present study (Figure S5D).

As a final control, we examined the degree of methylation in four known imprinted genes; *SNRPN*, *MEST*, *KCNQ1* and *IGF2*. As expected these genes showed approximately 50% methylation in islets from non-diabetic donors (Figure S5E).

### Impact of HbA1c, age and BMI on DNA methylation in human pancreatic islets

We then tested if known risk factors for T2D, including hyperglycemia, aging and obesity affect DNA methylation of the 1,649 CpG sites differentially methylated in T2D islets, already in non-diabetic subjects. The impact of HbA1c levels, age and BMI on DNA methylation of these 1,649 CpG sites was examined in pancreatic islets of 87 non-diabetic donors with HbA1c levels, age and BMI spanning between 4.3–6.4%, 26–74 years and 17.6–40.1 kg/m^2^ respectively (Table S6). HbA1c levels were associated with differential DNA methylation of 142 CpG sites (Table S7). Moreover, age and BMI were associated with differential DNA methylation of 28 and 16 CpG sites, respectively (Tables S8, S9). Interestingly, increased age was associated with decreased methylation of *CDKN1A* and increased methylation of *EXOC3L2* (Tables S8), which is in agreement with the results seen in T2D islets (Figure 3d and Figure 4e). Moreover, ∼92% of the CpG sites that exhibit differential DNA methylation due to increased HbA1c, age or BMI in non-diabetic islets changed in the same direction as methylation in T2D islets. These data suggest that increased HbA1c levels, aging and/or obesity may affect DNA methylation of CpG sites which are differentially methylated in T2D islets already in islets of non-diabetic subjects.

### Association between DNA methylation and expression in islets of non-diabetic donors

We also tested if there are significant associations between DNA methylation and gene expression of the 102 genes that exhibit both differential DNA methylation and gene expression in T2D versus non-diabetic islets, already in non-diabetic subjects. A linear regression model was used including batch, age, gender, BMI, HbA1c, islet purity and days of culture as covariates. In islets of the 87 non-diabetic donors, we found significant associations between DNA methylation and gene expression for 55 CpG sites in/near 43 genes (Table S10) out of the 149 CpG sites in/near 102 genes that exhibit both differential DNA methylation and gene expression in T2D versus non-diabetic islets (Table S4). The association between DNA methylation and gene expression for these 43 genes was in the same direction in both the 87 non-diabetic donors as in the T2D versus non-diabetic donors (Table S10 and Table S4). We further tested for associations between DNA methylation and gene expression in non-diabetic islets including all analyzed CpG sites on the Infinium HumanMethylation450 BeadChip in/near the 102 genes that show both differential DNA methylation and gene expression in T2D versus non-diabetic islets. We then found significant associations between DNA methylation and gene expression for 663 CpG sites in/near 57 genes out of the 102 studied genes.

### Expression of enzymes that regulate DNA methylation and demethylation

To identify factors that may contribute to differential DNA methylation in human pancreatic islets, we further tested if risk factors for T2D affect islet expression of a number of enzymes which are known to regulate DNA methylation and demethylation in mammals [50]. We found that the islet expression of *DNMT3b*, which is involved in *de novo* DNA methylation, correlated negatively with age (rho = −0.25, *P* = 0.02). In addition, exposure to lipids (1 mM palmitate) for 48 hours *in vitro* decreased the expression of two methyltransferases, *DNMT3a* (control islets 111.3±2.3 vs. lipid treated islets 107.7±2.0, *P* = 0.039, n = 13) and *DNMT1* (control islets 182.4±5.0 vs. lipid treated islets 154.6±6.3, *P* = 0.00005, n = 13), in human pancreatic islets. Lipid exposure also increased islet expression of *GADD45A* (control islets 489.4±23.7 vs. lipid treated islets 612.8±56.3, *P* = 0.010, n = 13), which encodes a protein involved in demethylation [50].

### DNA methylation in FACS sorted human β- and α-cells

**Figure 5:**
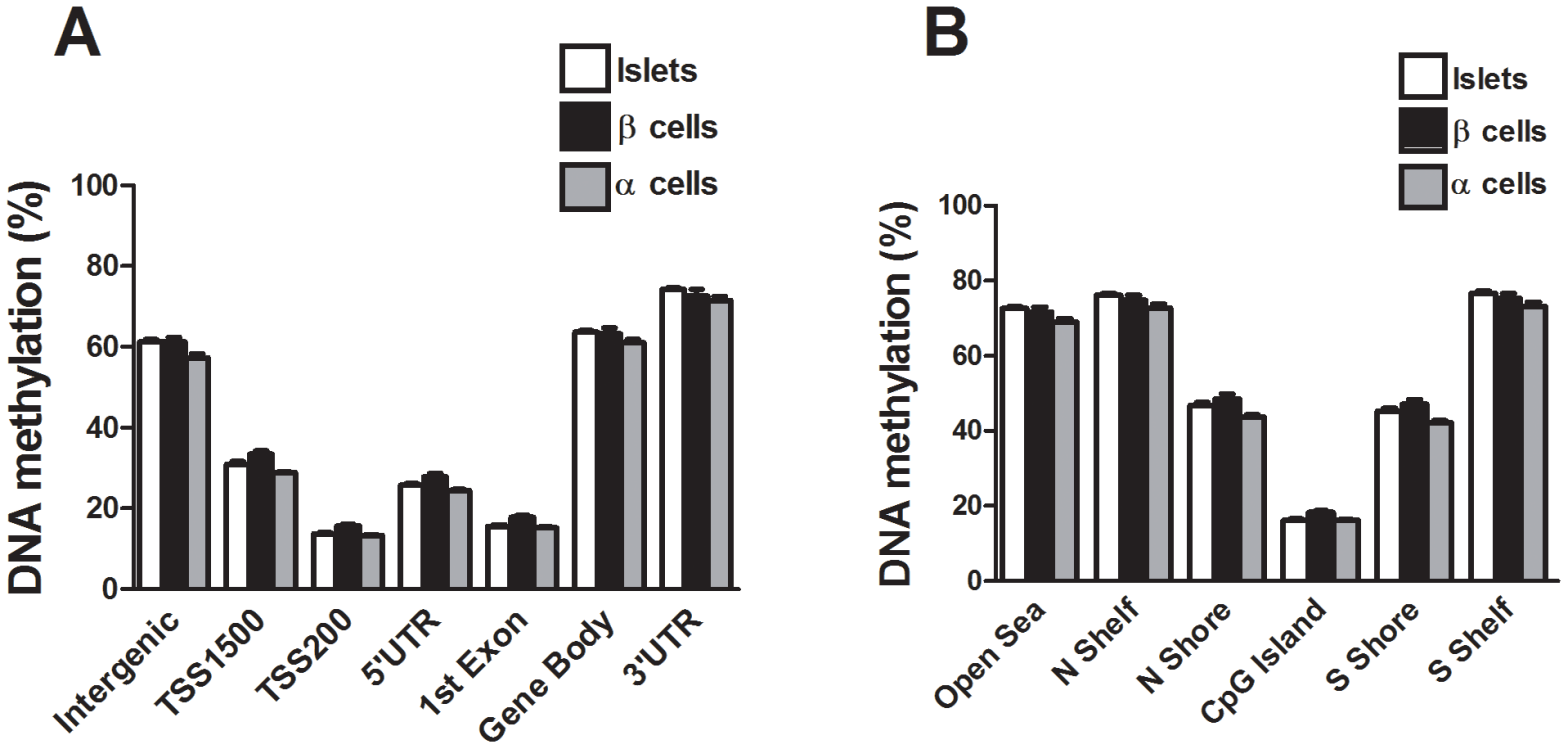
The human methylome in FACS sorted human β- and α-cell fractions. All analyzed DNA methylation sites on the Infinium HumanMethylation450 BeadChip are mapped to gene regions based on their functional genome distribution and CpG island regions based on CpG content and neighbourhood context [68]. Global DNA methylation in whole human islets (n = 4) and purified human β- (n = 3) and α-cell (n = 2) fractions is shown for (**A**) each gene region and (**B**) CpG island regions. Global DNA methylation is calculated as average DNA methylation based on all CpG sites in each annotated region on the chip. Data are mean ± SEM.

We next compared the DNA methylation pattern in whole human islets (n = 4) with DNA methylation in FACS sorted β- (n = 3) and α-cell (n = 2) fractions of non-diabetic donors. The donors of whole human islets are different from the donors of FACS sorted β- and α-cells. However, the whole islet donors and the donors of FACS sorted β- and α-cells were matched for age and BMI. To describe the overall methylome, we first calculated the average degree of DNA methylation in different genomic elements (Figure 1A) in human islets as well as in FACS sorted β- and α-cells. The global methylation pattern was similar in human islets and in FACS sorted β- and α-cells (Figure 5A–B). In both whole human islets and FACS sorted islet cells, the genomic regions close to the transcription start site showed relatively low degrees of methylation (TSS1500, TSS200, 5′UTR and 1^st^ exon) and there was a higher degree of methylation in regions further away from the transcription start site (gene body, 3′UTR and intergenic regions) (Figure 5A). Also in relation to CpG islands, the global methylation pattern was similar in human islets and in FACS sorted β- and α-cells (Figure 5B). We found that CpG islands were hypomethylated, shelves and open sea were hypermethylated, while shores showed an intermediate degree of methylation in whole human islets as well as in FACS sorted islet cells (Figure 5B). We then tested if any of the 1,649 CpG sites that were differentially methylated in T2D versus non-diabetic islets (Table S1) exhibit a different degree of methylation in whole human islets compared with purified human β-cells. Without correction for multiple testing, 132 of 1,649 CpG sites showed differential DNA methylation in whole islets compared with purified human β-cells at *P*<0.05, (Table S11). Moreover, among the candidate genes for T2D and obesity shown in Table 3, there was elevated DNA methylation in β-cells compared with human islets for three CpG sites at *P*<0.05 (cg26979504 in *HHEX*, cg03257822 in *HMGA2* and cg04920032 in *FAIM2*). However, no differences remained significant after correction for multiple testing.

## Discussion

Our study provides a detailed map of the human methylome in pancreatic islets from T2D and non-diabetic donors. We identified 1,649 individual CpG sites and 853 unique genes that exhibit differential DNA methylation, with absolute differences in methylation larger than 5%, representing a fold-change between 6–59%, in diabetic compared with non-diabetic islets. These include genes with previous known functions in pancreatic islets, the exocytosis process and apoptosis [28]–[45]. Recent GWAS have identified SNPs near/in genes that affect the risk for T2D [3]. We found differential DNA methylation of 17 T2D candidate genes, including *TCF7L2*, *THADA*, *KCNQ1*, *FTO* and *IRS1* in T2D islets. Several of these genes affect islet function and insulin secretion [4], [5]. It is possible that epigenetic modifications of T2D candidate genes in combination with genetic variation influence disease susceptibility [27]. Indeed, we have previously shown that ∼50% of SNPs associated with T2D are CpG-SNPs that introduce or delete possible DNA methylations sites [27]. Additionally, these T2D associated CpG-SNPs were associated with altered DNA methylation, gene expression, alternative splicing events and hormone secretion in human islets from non-diabetic donors [27]. Here, we demonstrate for the first time altered DNA methylation patterns of T2D candidate genes in human islets from patients with T2D. Our data propose that genetic and epigenetic mechanisms may interact to affect diabetes susceptibility and they show the importance of not just considering either genetics or epigenetics when dissecting factors that contribute to T2D. Our study also identified enrichments of differentially methylated genes in pathways in cancer, axon guidance, MAPK signaling pathway, focal adhesion and actin cytoskeleton in T2D islets. The significance of our data is supported by recent epidemiological studies that point to a close link between insulin resistance, T2D and cancer [51] as well as studies demonstrating central functions of MAPK signaling in pancreatic islets and diabetes [52]. Moreover, while focal adhesions play an important role in many signaling pathways and affect the ability of cells to interact with the extracellular matrix and respond efficiently to the dynamic microenvironment in age-related disease [53], it has been shown that genes involved in axon guidance, regulation of actin cytoskeleton and complement and coagulation cascades are differentially expressed in T2D patients [54]. Additionally, we found altered DNA methylation of members of the Plexin and Semaphorin families, which play a role in axon guidance and are suggested to control glucose homeostasis via regulating communication between pancreatic β-cells [55]. Some genes with differential DNA methylation in T2D islets e.g. *EGF* and *VEGFA* are part of several of the significant KEGG pathways. While EGF is a growth factor important for postnatal expansion of β-cell mass and for the survival of β-cells following stress induced apoptosis [56], VEGFA is responsible for dense islet vascularization and is expressed more in the endocrine than the exocrine part of the pancreas [57].

Hypermethylation of certain genomic regions may lead to suppressed transcription [8]. By combining genome-wide DNA methylation data with transcriptome profiles, we identified 102 genes that exhibit both differential DNA methylation and gene expression in diabetic islets. The majority of these genes showed decreased DNA methylation and increased gene expression in T2D islets. Our functional data further showed that increased DNA methylation of the human *CDKN1A* and *PDE7B* promoters decreased the transcriptional activity in clonal β-cells *in vitro*. However, based on these *in vitro* experiments it is still difficult to conclude whether altered DNA methylation *in vivo* has direct effects on gene expression. Moreover, DNA methylation may also regulate alternative splicing events [58] and/or transcription elongation efficiency via alternative promoters [59] when methylation takes place within gene bodies [27]. We found an overrepresentation of differentially methylated CpG sites in the gene body regardless of whether methylation and gene expression show a positive or inverse relationship. This is known as the DNA methylation paradox, which remains unexplained [46]. Interestingly, a recent study suggests a model by which the relationship between gene body DNA methylation and expression is bell shaped and varies depending on the transcriptional activity of the gene, meaning that high levels of gene body methylation are observed in genes with moderate expression levels while low levels of gene body methylation are observed in genes with low and high expression [60]. We also found an overrepresentation of CpG sites located within the open sea and the northern shore showing an inverse relationship between differential DNA methylation and gene expression in human T2D islets. Our data are supported by cancer studies where CpG island shore methylation was strongly related to gene expression [22].

The majority (97%) of the differentially methylated CpG sites showed decreased DNA methylation in diabetic islets. This may be explained by an increased expression and/or activity of proteins controlling demethylation. It could also be explained by a decreased activity/expression of the methyltransferase DNMT1 during cell replication or a decreased activity of DNMT3a and 3b, which are responsible for *de novo* methylation [50]. Interestingly, we found decreased expression of two methyltransferases and increased expression of *GADD45A*, which affects demethylation, in human pancreatic islets exposed to lipotoxicity, a risk factor for T2D. Another explanation for hypomethylation may be decreased levels of methyl donors [61].

T2D is a multifactorial polygenic disease and previous GWAS have identified more than 40 SNPs strongly associated with the disease. However, the effect size of these variants is modest and each SNP only explains a small proportion of the heritability for T2D [3]. Identified differences in gene expression of diabetic cases and controls have also been modest, but although alterations in each gene may only contribute with a small biological effect, together multiple changes in specific metabolic pathways are likely to increase the risk for disease [62], [63]. Moreover, in contrast to the big differences in DNA methylation that are found when comparing cancer and normal cells and which is probably due to the presence of an abnormal clone of cells, most of the absolute differences in DNA methylation that have been reported in non-cancer studies are modest in magnitude ranging from 0.13–11% [64]–[66]. In accordance with these studies, we also found that the absolute differences in methylation between the diabetic and non-diabetic islets ranged from 5–15%, which corresponds to a fold change of 6–59%. However, recent studies have shown that modest differences in DNA methylation of individual CpG sites can have big effects on gene expression and that in late onset diseases such as T2D small changes in gene expression may have a big effect on disease over long periods of time [64]. This is further supported by our *in vitro* experiments, where methylating only 2 or 6 CpG sites, respectively, in 1500 bp regions resulted in a profound decrease in gene activity.

To find support for the role of the identified genes in the pathogenesis of T2D, we manipulated the expression of selected genes in clonal β- and α-cells. Over-expression of Cdkn1a and Pde7b resulted in decreased glucose-stimulated insulin secretion in clonal β-cells. These experimental results are in agreement with our human data, where T2D islets show impaired glucose-stimulated insulin secretion in parallel with increased expression and decreased DNA methylation of *CDKN1A* and *PDE7B*. Together, our experimental and human data support a model where diabetes associated epigenetic modifications may lead to altered gene expression and subsequently impaired insulin secretion. CDKN1A, also known as p21, is a well characterized tumor suppressor and a negative regulator of the cell cycle [67]. Dependent on its cellular location, CDKN1A may also affect other cellular processes [31], [67]. However, there is limited information on its role and regulation in pancreatic islets. PDE7B is a cAMP-specific phosphodiesterase that regulates cellular cAMP levels [47]. Although this is the first study showing that PDE7B affects insulin secretion, other members of this family are known to control cAMP and insulin secretion in β-cells [48]. A deficient exocytosis machinery may result in perturbed insulin secretion [41]. Our study demonstrates for the first time that T2D islets exhibit decreased expression and increased DNA methylation of *EXOC3L2*, a member of the exocyst complex. Our *in vitro* experiments further show how Exoc3l deficiency results in decreased β-cell exocytosis. Even though the expression changes seen in clonal β-cells *in vitro* are bigger than the ones identified in human islets *in vivo*, it is likely that modest expression changes of multiple genes contribute to the disease phenotype in humans. Since both decreased insulin and increased glucagon secretion from pancreatic islets are known to contribute to hyperglycemia in patients with T2D [49], we investigated whether the identified candidate genes also affect glucagon secretion in clonal α-cells. Interestingly, overexpression of Cdkn1a and Pde7b resulted in elevated glucagon secretion at low glucose levels. Together, our functional data propose a model where identified candidate genes may contribute to hyperglycemia in T2D patients by both lowering insulin and increasing glucagon secretion from pancreatic islets.

A previous study analyzed DNA methylation of ∼27,000 CpG sites representing ∼0.1% of the CpG sites in the human genome in pancreatic islets from five T2D and 11 non-diabetic donors [14]. Volkmar *et al* used an array that mainly covers CpG islands in promoter regions and they found 276 CpG sites with differential DNA methylation with *P*<0.01 in T2D islets. However, since their data was not corrected for multiple testing, it may include false positives. Yet, we could replicate 71 of the differentially methylated sites identified by Volkmar *et al* and methylation data from the two islet studies correlated significantly [14]. In the present study, we could also validate data from our previous study where T2D was associated with differential DNA methylation of *INS* in human islets [13]. Our ability to confirm previous data support that T2D is associated with differential DNA methylation at specific sites. However, it should be noted that we used different bioinformatic and statistical analysis compared to Volkmar *et al*, e.g. we used quantile normalization and M-values and we analyzed differences in DNA methylation between T2D and non-diabetic islets using a linear regression model including batch, gender, BMI, age, islet purity and days of culture as covariates. Moreover, the array used in our study analyzes DNA methylation genome-wide of ∼480,000 CpG sites, covering most gene regions including promoters, 5′UTR, gene body and 3′UTR in ∼99% of RefSeq genes, as well as intergenic regions [68]. The array also covers CpG islands, shores and shelves as well as the open sea. Interestingly, we found that differentially methylated CpG sites were underrepresented in CpG islands and overrepresented in the open sea, which are isolated CpG sites throughout the genome. It may thereby be difficult to identify differentially methylated CpG sites in T2D islets using an array that mainly covers CpG islands such as the Infinium 27k array.

While some researchers have found a decreased β-cell number and increased α-cell number in human T2D compared with non-diabetic islets, others have not found any differences [69]–[71]. We found no differences in β-cell content in T2D versus non-diabetic islets, nor did Volkmar *et al* [14], suggesting that the differences seen in DNA methylation are not due to altered β-cell composition in islets from T2D patients. Although it would be of interest to analyze DNA methylation in isolated insulin-producing human β-cells, the sorting of these cells is technically difficult, which results in loss of many cells and in addition, may affect their function [72]. Indeed, there are only a few studies to date that have used isolated human β-cells with a maximum number of 16 donors [12], [13], [73]–[75]. While we and others have found epigenetic differences between human β- and α-cells from a modest number of non-diabetic donors [12], [13], [74], [75], to our knowledge there is no available data showing differential DNA methylation in human β-cells from patients with T2D compared with non-diabetic donors, nor have any studies analyzed gene expression genome-wide in human β- and α-cells from patients with T2D. Here, we analyzed for the first time DNA methylation genome-wide in purified β-and α-cell fractions from non-diabetic human islets donors. The global DNA methylation pattern was similar in purified human β-cells and whole islets. Moreover, for the 1,649 CpG sites showing differential DNA methylation in T2D versus non-diabetic human islets, we could not detect any significant differences in methylation between purified human β-cells and whole islets. Importantly, most of the other islet cell types such as glucagon-producing α-cells, somatostatin-producing δ-cells and pancreatic polypeptide producing PP-cells also have key effects on whole body glucose homeostasis [76]. Differential DNA methylation and gene expression in the majority of islet cells may thereby affect the pathogenesis of T2D and studying epigenetic modifications in whole human pancreatic islets is therefore physiologically warranted. Indeed, our functional studies show effects of candidate genes identified in whole islets in both clonal β- and α-cells. However, future studies should also address if there are epigenetic differences in sorted β- and α-cells from diabetic versus non-diabetic donors.

Interestingly, risk factors for T2D such as hyperglycemia (HbA1c), aging and BMI were associated with differential DNA methylation already in islets of non-diabetic human donors. It is hence possible that differential DNA methylation in islets predisposes to disease. Nevertheless, we cannot rule out that some of the identified epigenetic differences are secondary to disease or epiphenomenon [77]. However, excluding epiphenomenon would require a longitudinal study taking pancreatic biopsies at different time points which is not ethically possible in humans. Nevertheless, previous animal studies support that epigenetic modifications, taking place in pancreatic islets at an early age due to an unfavorable fetal environment, can predispose to diabetes in adult life [15]–[17], [78]. Importantly, Thompson *et al* found differential DNA methylation of ∼1,400 CpG sites together with altered gene expression in islets of seven week old rats exposed to an intrauterine growth restriction, a model that causes diabetes in elderly rats [78]. Our recent studies further support that altered DNA methylation may play a role in the pathogenesis of T2D as we found positive correlations between HbA1c levels and DNA methylation of *INS* and *PDX-1* in human islets and elevated glucose levels had direct effects on DNA methylation of *INS* and *PDX-1* in clonal β-cells [12], [13]. Additionally, DNA methylation of *INS* and *PDX-1* was increased in islets from T2D patients compared with controls. Also, as epigenetic modifications are tissue specific, using a surrogate tissue like blood is unlikely to give the same result, which has been shown in previous studies [13], [14]. Moreover, our functional data support an important role of identified candidate genes on islet function and in the pathogenesis of T2D.

Recent studies demonstrate that some probes on Illumina's DNA methylation chip can cross-react to multiple locations in the genome [25]. However, none of the 1,649 probes used to detect differential methylation in our study have a perfect match elsewhere in the genome and only 14 probes have a near-perfect match.

Overall, our study identified novel epigenetic modifications in T2D patients that contribute to differential gene expression and perturbed insulin secretion, a key characteristic of T2D. Our genome-wide DNA methylation data can furthermore serve as a reference methylome for human pancreatic islets.

## Materials and Methods

### Ethics statement

The pancreatic islet donor or her/his relatives had given their consent to donate organs for medical research upon admission to intensive care unit. All procedures were approved by ethics committees at Uppsala and Lund Universities.

### Human pancreatic islet donors

Human pancreatic islets from 15 donors with T2D and 34 donors not diagnosed with diabetes were included in the genome-wide analysis of DNA methylation and mRNA expression. Donors were considered to have T2D if they had been diagnosed with the disease prior to their death. Selection criteria for non-diabetic donors were to have an HbA1c below 6.0%. Clinical characteristics of these donors are given in Table 1. Moreover, the impact of HbA1c levels, age and BMI on DNA methylation was studied in pancreatic islets from 87 non-diabetic donors. Their characteristics are given in Table S6. Human pancreatic islets were provided by the Nordic Network for Islet Transplantation, Uppsala University, Sweden.

### Sample processing of human islets

Human islets were prepared by collagenase digestion and density gradient purification. Prior to nucleic acid purification, islets were cultured for 2.7±0.15 days as previously described [63]. DNA and RNA were extracted with the All Prep DNA/RNA kit (Qiagen, Hilden, Germany) and purity and concentration were determined by using a nanodrop (NanoDrop Technologies, Wilmington, USA). The purity of the islet preparations was determined by expression of endocrine (somatostatin and glucagon) and exocrine (pancreatic lipase, amylase α2A, chymotrypsin 2) markers and dithizone staining [63]. β-cell content in human islets of donors with available embedded islets (6 T2D and 13 non-diabetic donors) was analyzed using transmission electron microscopy. Hand-picked islets where fixed in 2.5% glutaraldehyde in freshly prepared Millonig and post-fixed in 1% osmium tetroxide before being dehydrated and embedded in AGAR 100 (Oxford Instruments Nordiska, Lidingö, Sweden) and cut into ultrathin sections as described [5]. The sections were put on Cu-grids and contrasted using uranyl acetate and lead citrate. The islet containing sections were examined in a JEM 1230 electron microscope (JEOL-USA. Inc., MA). Micrographs were analyzed for β-cell content with ImageJ and in-house software programmed in Matlab using methods previously described [14], [79]. Islet cell types were distinguished by means of granular appearance: β-cell granules have a dense core surrounded by a white halo and α-cells have small dense granules. The ratio of β-cells in each islet was calculated by division of the total number of β-cells by the sum of the β-cell and α-cell numbers. Furthermore, glucose-stimulated insulin secretion from human pancreatic islets was measured as described [80].

### Purification of human β- and α-cell fractions

β- and α-cells were purified from pancreatic islets of three human donors (aged 54, 55 and 74 years old, with BMI 21.5–23.1 kg/m^2^), different from the donors described in Table 1 and Table S1, by using a method previously described by Parnaud *et al* [73]. Dissociation of islet cells was achieved by incubation with constant agitation for 3 minutes at 37°C in 0.05% trypsin-EDTA (Invitrogen) supplemented with 3 mg/ml DNAse I (Roche, Mannheim, Germany) followed by vigorous pipetting. Labelling and FACS sorting of the β- and α-cell fractions was performed as previously described [73]. Sorted β- and α-cells were applied to microscope slides and co-immunostained for insulin and glucagon in order to detect the amount of α-cells in the β-cell fraction, and vice versa. Using this method, a cell purity of 89±9% (mean ± SD) was achieved [81]. After DNA isolation using Qiagen DNEasy blood and tissue kit (Qiagen) and ethanol precipitation, DNA for genome-wide methylation profiling was available for β- and α-cell fractions from 3 and 2 donors, respectively.

### Genome-wide DNA methylation profiling

Genome-wide DNA methylation profiling of human pancreatic islets and purified human β- and α-cell fractions was performed at the SCIBLU genomics center at Lund University with the Infinium HumanMethylation450 BeadChip (Illumina, Inc., San Diego, CA, USA) which interrogates 482,421 CpG sites, 3091 non-CpG sites and 65 random SNPs and covers 21,231 RefSeq genes [68]. 500 ng DNA from human pancreatic islets was bisulfite converted using the EZ DNA Methylation Kit D5001 (Zymo Research, Orange, CA, USA) according to the manufacturer's instructions. Bisulfite converted DNA was amplified, fragmented and hybridized to the BeadChips following the standard Infinium protocol. T2D islet samples were randomized across the chips and all samples were analyzed on the same machine by the same technician to reduce batch effects. After single base extension and staining, the BeadChips were imaged with the Illumina iScan. Raw fluorescence intensities of the scanned images were extracted with the GenomeStudio (V2011.1) Methylation module (1.9.0) (Illumina). The fluorescence intensity ratio was used to calculate a β-value which corresponds to the methylation score for each analyzed site according to the following equation: β-value = intensity of the Methylated allele (M)/(intensity of the Unmethylated allele (U)+intensity of the Methylated allele (M)+100). DNA methylation β-values range from 0 (completely unmethylated) to 1 (completely methylated). All samples had high bisulfite conversion efficiency (intensity signal >4000) and they were included for further analysis based on GenomeStudio quality control steps where control probes for staining, hybridization, extension and specificity were examined. The intensity of both sample dependent and sample independent built in controls was checked for the red and green channels using GenomeStudio.

We next exported the DNA methylation data from GenomeStudio and used Bioconductor [82] and the lumi package [83] for further analyses. Individual probes were filtered based on their mean detection *P*-value and those with a *P*-value>0.01 were excluded from further analysis. As a result, DNA methylation data for 483,031 (99.5%) probes, including 479,927 CpG sites and 3,039 non-CpG sites were used for further analysis. Because M-values are more statistically valid [84], β-values were converted to M-values using the following equation: M = log_2_ β-value/(1−β-value). M-values were then used for further statistical analysis [84]. In order to correct for background fluorescence the median M-value of the built in negative controls was subtracted from M-values. Next a quantile normalization was performed as described [85]. The Universal Methylated Human DNA standard D5011 (Zymo Research), which is human DNA that has been enzymatically methylated in the CpG sites by M.SssI methyltransferase, was used as a positive control in every batch and it showed high levels of methylation (87.7±0.6%). The low intensity of Y chromosome loci in female samples was used as an additional control. Moreover, one human pancreatic islet sample was run in two different batches on two different days and used as a technical replicate. As the β-value is easier to interpret biologically, M-values were reconverted to β-values when describing the results and creating the figures.

### Pathway analysis

The enrichment of KEGG pathways among genes that exhibit differential DNA methylation in T2D compared with non-diabetic islets was tested using WebGestalt (http://bioinfo.vanderbilt.edu/webgestalt, March 2012).

### Microarray mRNA expression analysis

mRNA expression of the human pancreatic islets was analyzed using the GeneChip Human Gene 1.0 ST array from Affymetrix (Santa Clara, CA, USA) as previously described [63].

### Luciferase assays

1500 bp of the human *CDKN1A* or *PDE7B* promoters (sequences are given in Table S12) were inserted into a CpG-free firefly luciferase reporter vector (pCpGL-basic) kindly provided by Dr Klug and Dr Rehli [86]. Amplification of *CDKN1A* and *PDE7B* DNA sequences and insertion into the pCpGL-basic vector was done by GenScript (Piscataway, NJ, USA). The constructs were either mock-methylated or methylated using two different DNA methyltransferases; *Sss*I and *Hha*I (New England Biolabs, Frankfurt am Main, Germany). While *Sss*I methylates all cytosine residues within the double stranded dinucleotide recognition sequence CG, *Hha*I only methylates the internal cytosine residue in GCGC sequence. INS-1 832/13 β-cells were co-transfected with 100 ng pCpGL-vector either with or without respective insert together with 2 ng of pRL renilla luciferase control reporter vector (pRL-CMV vector, Promega, Madison, USA) as a control for transfection efficiency and luciferase activity was measured as previously described [12].

### Overexpression of Cdkn1a, Pde7b and Sept9 in clonal β- and α-cells

INS-1 832/13 β-cells were cultured as previously described [87] and αTC1-6 cells were cultured according to ATCC's instructions (ATCC, Manassas, VA). pcDNA3.1 expression vectors with rat cDNA for either *Cdkn1a*, *Pde7b* or *Sept9*, or the empty vector, were transfected into β- or α-cells with Lipofectamine LTX and Plus Reagent (Life Technologies, Paisley, UK), according to the manufacturer's instructions (sequences for *Cdkn1a*, *Pde7b* or *Sept9* are given in Table S13). Overexpression was verified with real-time PCR using an ABI 7900 system (Applied Biosystems, Foster City, CA, USA) and a SYBR Green assay for *Cdkn1a* (fwd-primer: ATGTCCGACCTGTTCCACAC, rev-primer: CAGACGTAGTTGCCCTCCAG) or TaqMan assays (Life Technologies) for *Pde7b* (Rn00590117_m1) and *Sept9* (Rn00582942_m1). Cyclophilin B (Rn03302274_m1 and Mm00478295_m1) was used as an endogenous control. Expression levels were calculated with the ΔΔCt method. Overexpression was also verified by Western Blot analysis and cells transfected with HA-tagged cDNAs for Cdkn1a, Pde7b and Sept9 were lysed in RIPA buffer (50 mM Tris pH 7.6, 150 mM NaCl, 0.1% SDS, 0.5% sodium deoxycholate, 1% Triton×100 and 1× protease inhibitor cocktail (P8340, Sigma-Aldrich, USA) and boiled with 6× sample buffer (60 mM Tris pH 6.8, 10% glycerol, 2% SDS, 10% β-mercaptoethanol and bromophenol blue). Samples were separated on gradient Mini-PROTEAN® TGX gels (Bio-Rad, Hercules, CA, USA) and transferred onto Hybond-LFP PVDF membranes (GE Healthcare, Piscataway, NJ, USA). Protein expression was detected with primary antibodies against HA tag (ab9110, Abcam Cambridge, UK) and β-actin (A5441, Sigma-Aldrich) and secondary DyLight 680/800 conjugated anti-mouse and anti-rabbit antibodies (35518 and 35571, Thermo Scientific, Rockford, USA) and blots were scanned in an ODYSSEY (Licor, Lincoln, NE, USA).

### Insulin secretion and content

48 hours post transfection of INS-1 832/13 β-cells, insulin secretion with indicated secretagogues was determined during 1 hour static incubations as previously described [87]. Insulin content of cells was determined after acid ethanol extraction of the hormone. Insulin secretion was normalized to total insulin content.

### Glucagon secretion

αTC1-6 cells were transfected as described above. 48 hours post transfection clonal α-cells were pre-incubated in HEPES balanced salt solution (HBSS, 114 mM NaCl; 4.7 mM KCl; 1.2 mM KH_2_PO_4_; 1.16 mM MgSO_4_; 20 mM HEPES; 2.5 mM CaCl_2_; 25.5 mM NaHCO_3_; 0.2% BSA, pH 7.2) supplemented with 5.5 mM glucose. Secretion was then stimulated in 1 hour static incubation with HBSS supplemented with 1 or 16.7 mM glucose. Secreted glucagon was measured with a glucagon ELISA (Mercodia, Uppsala, Sweden) and normalized to total protein as determined by a BCA assay (Thermo Scientific).

### Proliferation assay

INS-1 832/13 β-cells were transfected as described above. 72 hours post transfection the β-cells were washed with PBS and stained with 0.1% crystal violet in 0.15 M NaCl. Cells were then washed with water and allowed to dry. Methanol was added to wells and absorbance measured at 600 nm in an Infinite M200 plate reader (Tecan, Männerdorf, Switzerland).

### RNA interference

INS-1 832/13 β-cells were transfected with Lipofectamine RNAiMAX (LifeTechnologies) according to the manufacturer's instructions with siRNA targeting *Exoc3l* (LifeTechnologies, ID: s146127) or negative control siRNA (5′-GAGACCCUAUCCGUGAUUAUU-3′). Following 24 hours incubation, cells were transferred onto Petri dishes and cultured another 24 hours. *Exoc3l* knock-down was verified with real-time PCR using an ABI 7900 system and assays for *Exoc3l* (Rn01432027_m1) and endogenous controls (Cyclophilin B, Rn03302274_m1 and *Hprt*, Rn01527840_m1) (Life Technologies).

### Electrophysiological measurements of exocytosis using the patch-clamp technique

Electrophysiological measurements of exocytosis were performed on INS-1 832/13 β-cells as described [88].

### Analysis of DNA methylation of selected genomic regions

Pyrosequencing was used to technically validate the Infinium HumanMethylation450 BeadChip DNA methylation data. EpiTect Bisulfite Kit (Qiagen) was used for bisulfite conversion of human islet DNA. Primers were designed using the PyroMark Assay design Software 2.0 (Qiagen). Sequences are included in Table S14. Bisulfite converted DNA was amplified with the PyroMark PCR kit. Pyrosequencing was performed with PyroMark ID 96 and PyroMark Gold Q96 reagents (Qiagen) according to the manufacturer's instructions. Data were analyzed with the PyroMark Q96 2.5.7 software program.

Sequenom's MassARRAY EpiTYPER protocol (Sequenom, San Diego, CA, USA) was used to measure DNA methylation of *PDX1* in its distal promoter and enhancer regions according to our previous study [12].

### Statistical analysis

A principle component analysis was performed to examine batch effects and other possible sources of variation on the DNA methylation data. To identify differences in DNA methylation and mRNA expression between T2D and non-diabetic islets a linear regression model was used including batch, gender, BMI, age, islet purity and days of culture as covariates and DNA methylation or mRNA expression as quantitative variables. A false discovery rate (FDR) analysis was used to correct for multiple testing [18], [89], [90]. *Chi2* tests were used to compare the expected number of probes on the Infinium HumanMethylation450 BeadChip with observed number of differentially methylated probes in T2D islets.

## Supporting Information

Figure S1 Islet purity and β-cell content in human pancreatic islets. There were no significant differences in (A) purity or (B) β-cell content in pancreatic islets of non-diabetic compared with T2D human donors. The β-cell content was analyzed in 4.11±0.46 islets/donor. Mann-Whitney U test was used for statistical analysis and data are presented as mean ± SEM.
(TIF)

Figure S2 Distribution of non-CpG sites analyzed with the Infinium HumanMethylation450 BeadChip based on their (A) functional genome distribution and (B) relation to CpG island regions.
(TIF)

Figure S3 Impact of Cdkn1a, Pde7b and Sept9 on clonal β- and α-cells. Clonal INS-1 832/13 β-cells and αTC1-6 cells were used to study the impact of Cdkn1a, Pde7b and Sept9 on insulin and glucagon secretion, respectively. (A) Glucose-stimulated insulin secretion represented as the ratio of secretion at 16.7 over that at 2.8 mM glucose (fold change) in clonal β-cells overexpressing either Cdkn1a, Pde7b or Sept9 (black bars) compared with cells transfected with an empty pcDNA3.1 vector (white bar) (n = 5). * *P*≤0.05. (B) Insulin secretion in response to 2.8 mM glucose (white bars) or 2.8 mM glucose+35 mM KCl (black bars) in clonal β-cells overexpressing either Cdkn1a, Pde7b or Sept9 compared with cells transfected with an empty pcDNA3.1 vector (n = 4). (C) Fold-change of insulin secretion at 2.8 mM glucose+35 mM KCl over that at 2.8 mM glucose in clonal β-cells overexpressing either Cdkn1a, Pde7b or Sept9 (black bars) compared with control cells transfected with an empty pcDNA3.1 vector (white bar) (n = 4). * *P*≤0.05. (D) Overexpression of *Cdkn1a*, *Pde7b* and *Sept9* with pcDNA3.1 expression vectors in clonal α-cells (αTC1-6) resulted in elevated mRNA levels (black bars) compared with cells transfected with an empty pcDNA3.1 vector (white bars) (n = 4), * *P*≤0.05. Overexpression at the protein level was determined by western blot with an anti HA-tag antibody.
(TIF)

Figure S4 Technical validation of Infinium HumanMethylation450 BeadChip data. A human islet sample was bisulfite converted and analyzed on the Infinium array at two different occasions. The correlation for DNA methylation of 100 000 CpG sites from the two arrays was then calculated and presented in panel A. Seven CpG sites including cg21091547 (*CDKN1A*), cg27306443 (*PDE7B*), cg19654743 (*SEPT9*), cg04751089 (*IRS1*), cg20995304 (*HDAC7*), cg01649611 (*THADA*) and cg15572489 (*PTPRN2*) were selected for technical validation of the Infinium HumanMethylation450 BeadChip data using pyrosequencing. Correlations between DNA methylation data analyzed with the two different methods were all significant and are shown in panel B. Correlations were calculated using Spearman's test.
(TIF)

Figure S5 Confirmation of Infinium HumanMethylation450 BeadChip data. DNA methylation data of 264 CpG sites analyzed in human pancreatic islets from both our study and in the study by Volkmar *et al* correlate positively in (A) non-diabetic and (B) T2D donors. Increased DNA methylation of CpG sites in the *INS* and *PDX1* genes in T2D versus non-diabetic islets (* *P*<0.05) is shown in panel C and D, respectively. The degree of DNA methylation of CpG sites in previously known imprinted genes in pancreatic islets of non-diabetic donors is shown in panel E.
(TIF)

Table S1 CpG sites with differential DNA methylation (*q*<0.05 and difference in DNA methylation ≥5%) in pancreatic islets from 34 non-diabetic versus 15 T2D human donors.
(XLSX)

Table S2 DMRs with differential DNA methylation (*q*<0.05 and difference in methylation ≥5%) in pancreatic islets from 34 non-diabetic versus 15 T2D human donors.
(DOCX)

Table S3 Non-CpG DNA methylation in human pancreatic islets.
(DOCX)

Table S4 CpG sites with differential DNA methylation (*q*<0.05 and difference in methylation ≥5%) concurrent with a difference in mRNA expression (*P*≤0.05) of the nearest gene in pancreatic islets from 34 non-diabetic versus 15 T2D human donors.
(XLSX)

Table S5 CpG sites that exhibit differential DNA methylation in pancreatic islets from T2D compared with non-diabetic islets in the study by Volkmar *et al* as well as in the present study with *P*<0.05. DNA methylation data from the 34 non-diabetic and 15 T2D human donors analyzed in the present study is presented in this table.
(DOCX)

Table S6 Characteristics of 87 non-diabetic human donors of pancreatic islets used to examine the impact of HbA1c, age and BMI on DNA methylation of the 1,649 CpG that exhibit differential DNA methylation in pancreatic islets from 34 non-diabetic versus 15 T2D human donors.
(DOCX)

Table S7 CpG sites that exhibit differential DNA methylation (*q*<0.05 and difference in methylation ≥5%) in pancreatic islets from 34 non-diabetic versus 15 T2D human donors in parallel with an association between HbA1c levels and differential DNA methylation (*P*<0.05) in pancreatic islets from 87 non-diabetic donors.
(DOCX)

Table S8 CpG sites that exhibit differential DNA methylation (*q*<0.05 and difference in methylation ≥5%) in pancreatic islets from 34 non-diabetic versus 15 T2D human donors in parallel with an association between age and differential DNA methylation (*P*<0.05) in pancreatic islets from 87 non-diabetic donors.
(DOCX)

Table S9 CpG sites that exhibit differential DNA methylation (*q*<0.05 and difference in methylation ≥5%) in pancreatic islets from 34 non-diabetic versus 15 T2D human donors in parallel with an association between BMI and differential DNA methylation (*P*<0.05) in pancreatic islets from 87 non-diabetic donors.
(DOCX)

Table S10 Associations between DNA methylation and gene expression in pancreatic islets from 87 non-diabetic donors of CpG sites also showing differential DNA methylation (*q*<0.05 and difference in methylation ≥5%) concurrent with a difference in mRNA expression (*P*≤0.05) of the nearest gene in pancreatic islets from 34 non-diabetic versus 15 T2D human donors.
(DOCX)

Table S11 DNA methylation in whole islets (n = 4) compared to FACS sorted β-cells (n = 3) of the 1649 CpG sites that showed differential methylation in pancreatic islets from 34 non-diabetic versus 15 T2D human donors.
(XLSX)

Table S12 Sequences of *CDKN1A* and *PDE7B* inserted into the CpG-free firefly luciferase reporter vector (pCpGL-basic) and used for luciferase experiments.
(DOCX)

Table S13 Sequences of *Cdkn1a*, *Pde7b* and *Sept9* inserted into pcDNA3.1 expression vectors and used for the overexpression experiments.
(DOCX)

Table S14 DNA sequences for pyrosequencing forward, reverse and sequencing primers.
(DOCX)
