# Supplementary figures and images for: Genome-Wide DNA Methylation Analysis of Human Pancreatic Islets from Type 2 Diabetic and Non-Diabetic Donors Identifies Candidate Genes That Influence Insulin Secretion
Source: PLoS Genet. 2014 Mar 6;10(3):e1004160. doi: 10.1371/journal.pgen.1004160 (PMC3945174; doi:10.1371/journal.pgen.1004160)

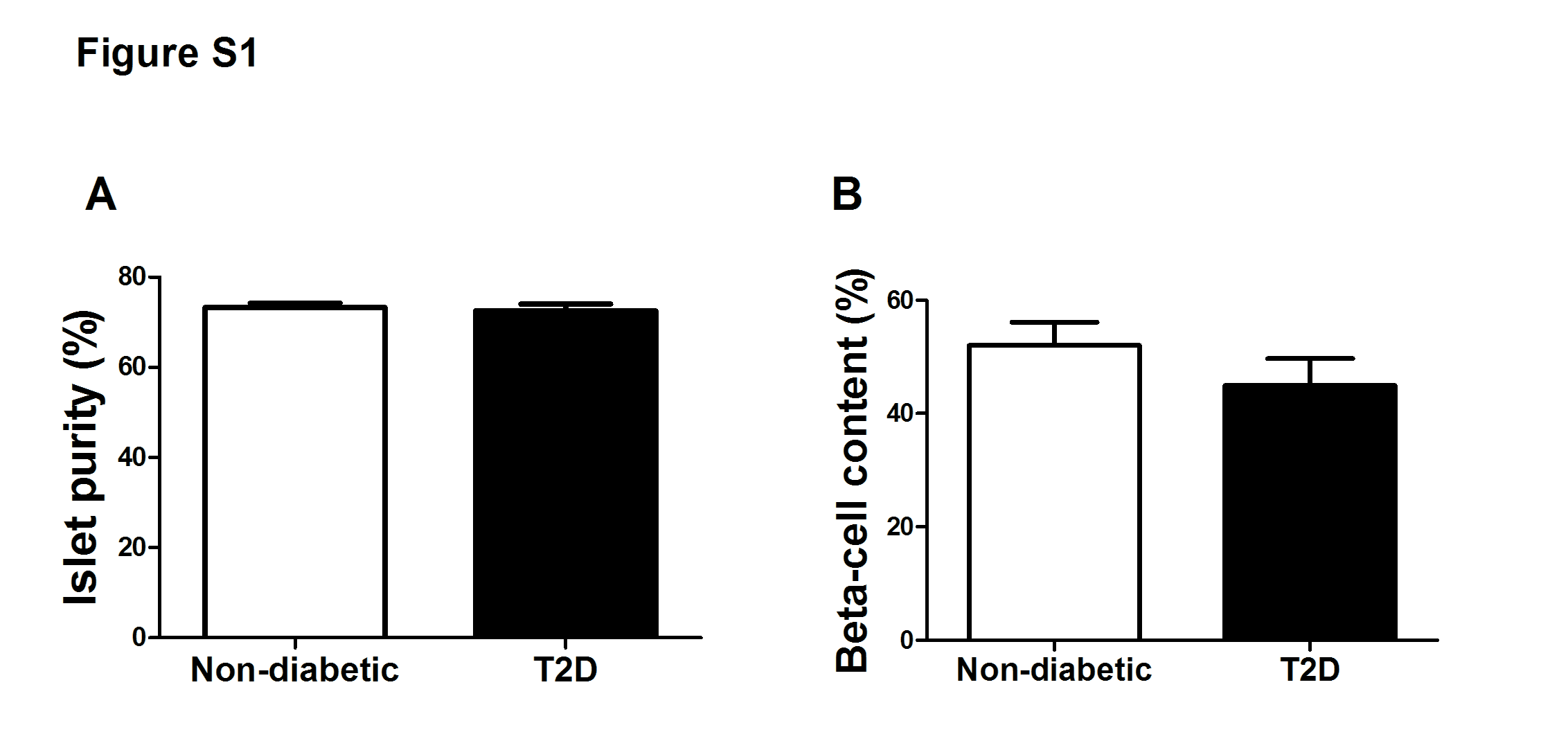

Supplement: Figure S1 — Islet purity and β-cell content in human pancreatic islets. There were no significant differences in (A) purity or (B) β-cell content in pancreatic islets of non-diabetic compared with T2D human donors. The β-cell content was analyzed in 4.11±0.46 islets/donor. Mann-Whitney U test was used for statistical analysis and data are presented as mean ± SEM. (TIF) [file pgen.1004160.s001.tif]

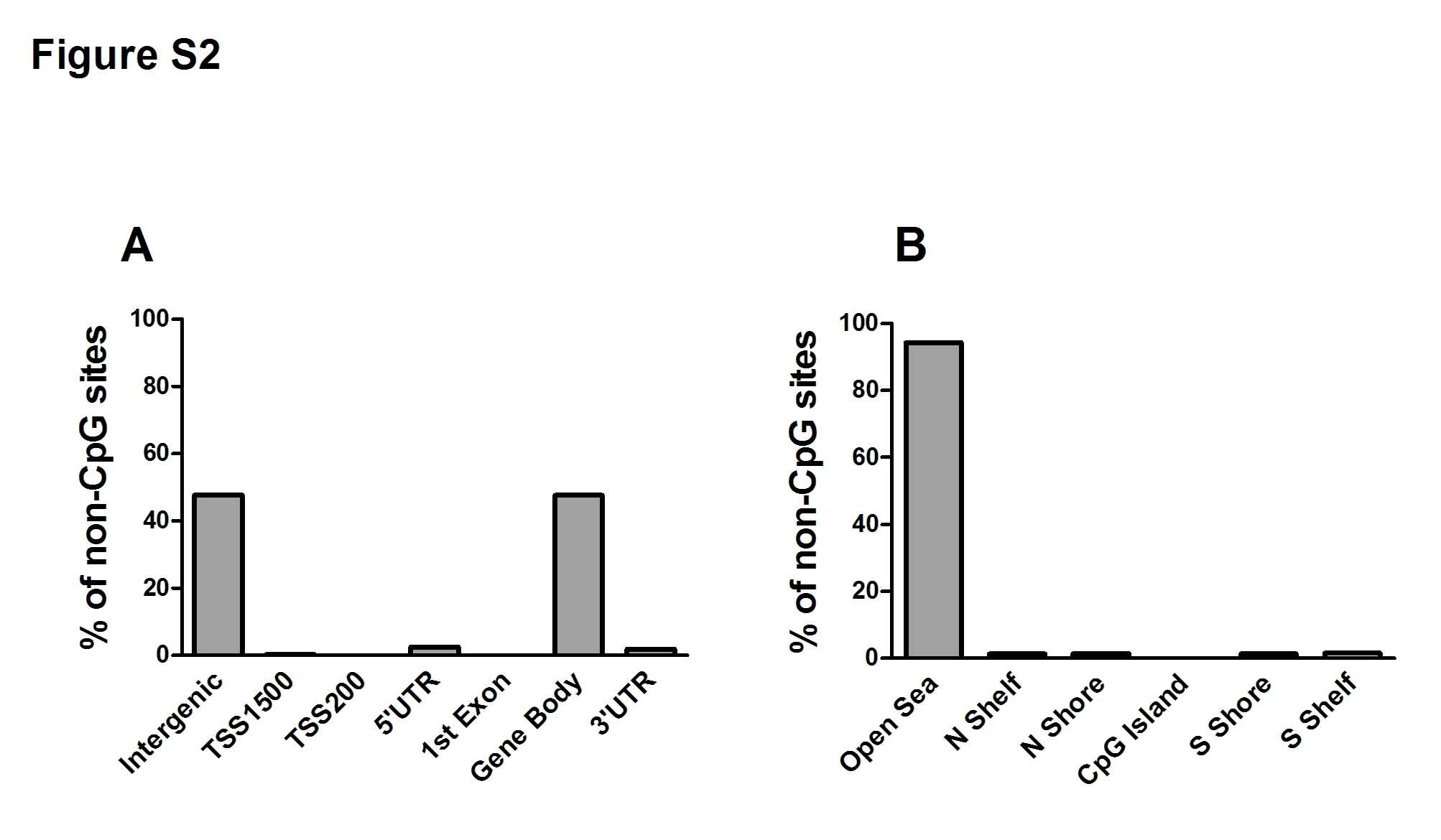

Supplement: Figure S2 — Distribution of non-CpG sites analyzed with the Infinium HumanMethylation450 BeadChip based on their (A) functional genome distribution and (B) relation to CpG island regions. (TIF) [file pgen.1004160.s002.tif]

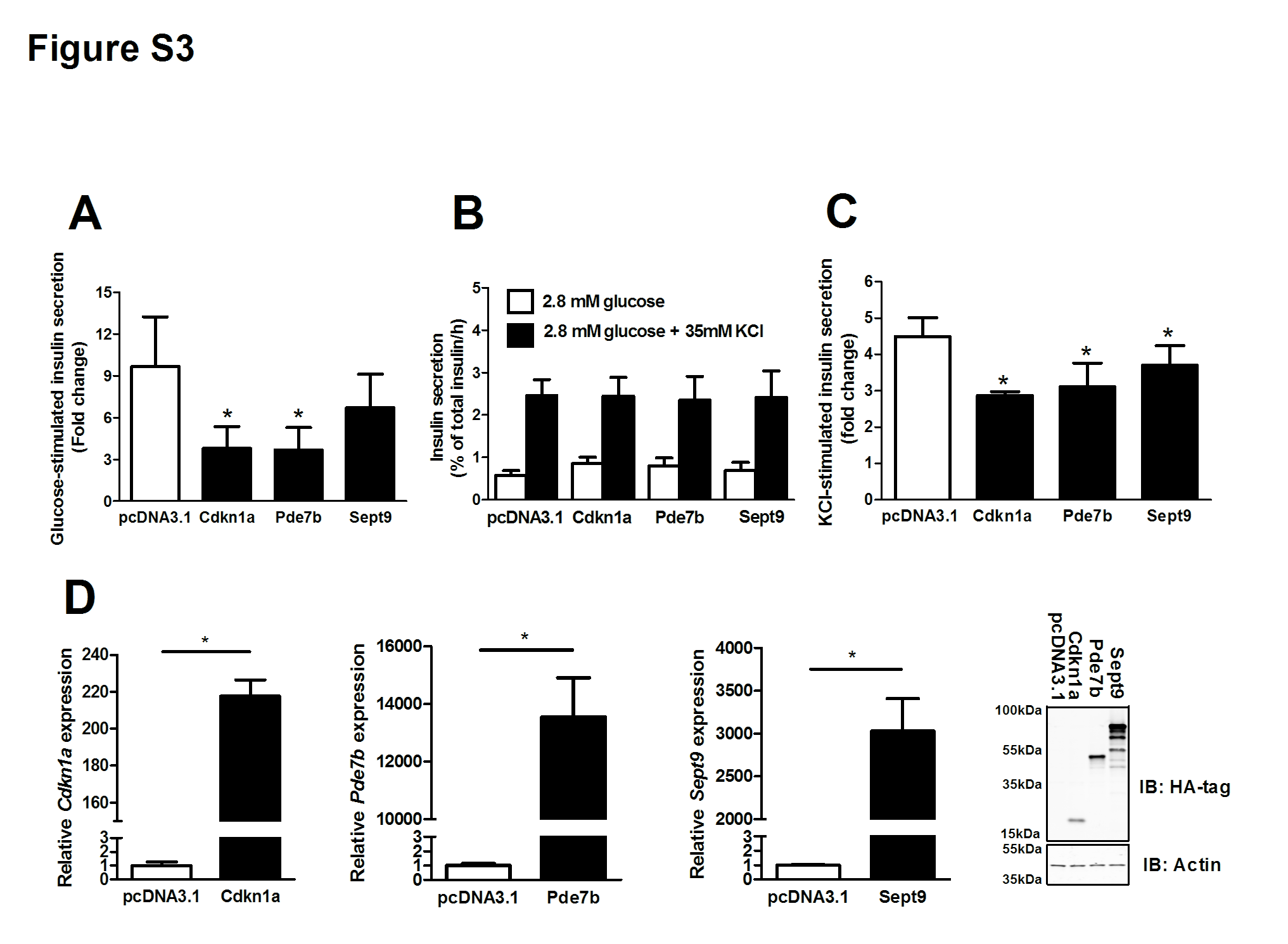

Supplement: Figure S3 — Impact of Cdkn1a, Pde7b and Sept9 on clonal β- and α-cells. Clonal INS-1 832/13 β-cells and αTC1-6 cells were used to study the impact of Cdkn1a, Pde7b and Sept9 on insulin and glucagon secretion, respectively. (A) Glucose-stimulated insulin secretion represented as the ratio of secretion at 16.7 over that at 2.8 mM glucose (fold change) in clonal β-cells overexpressing either Cdkn1a, Pde7b or Sept9 (black bars) compared with cells transfected with an empty pcDNA3.1 vector (white bar) (n = 5). * P≤0.05. (B) Insulin secretion in response to 2.8 mM glucose (white bars) or 2.8 mM glucose+35 mM KCl (black bars) in clonal β-cells overexpressing either Cdkn1a, Pde7b or Sept9 compared with cells transfected with an empty pcDNA3.1 vector (n = 4). (C) Fold-change of insulin secretion at 2.8 mM glucose+35 mM KCl over that at 2.8 mM glucose in clonal β-cells overexpressing either Cdkn1a, Pde7b or Sept9 (black bars) compared with control cells transfected with an empty pcDNA3.1 vector (white bar) (n = 4). * P≤0.05. (D) Overexpression of Cdkn1a, Pde7b and Sept9 with pcDNA3.1 expression vectors in clonal α-cells (αTC1-6) resulted in elevated mRNA levels (black bars) compared with cells transfected with an empty pcDNA3.1 vector (white bars) (n = 4), * P≤0.05. Overexpression at the protein level was determined by western blot with an anti HA-tag antibody. (TIF) [file pgen.1004160.s003.tif]

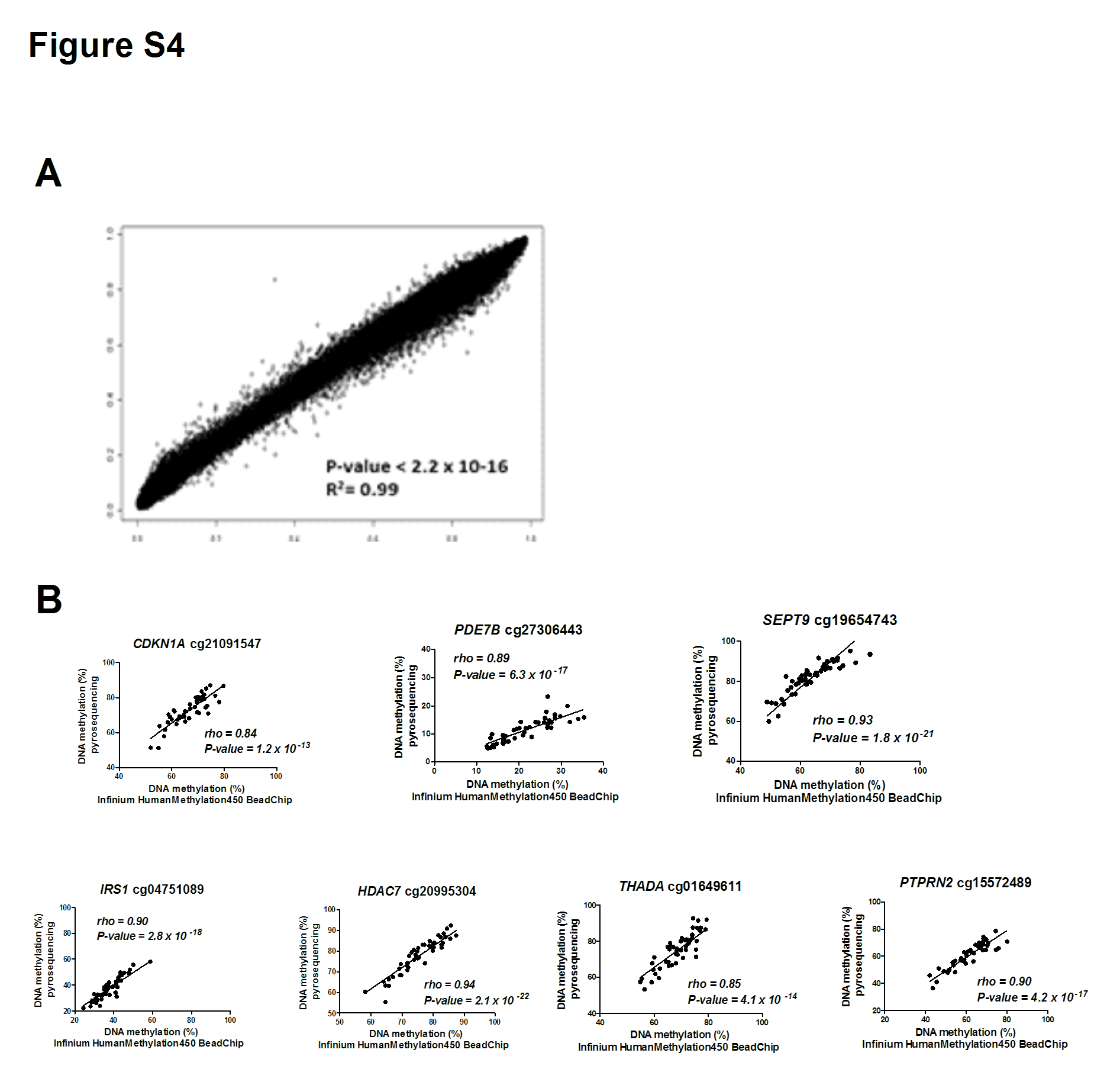

Supplement: Figure S4 — Technical validation of Infinium HumanMethylation450 BeadChip data. A human islet sample was bisulfite converted and analyzed on the Infinium array at two different occasions. The correlation for DNA methylation of 100 000 CpG sites from the two arrays was then calculated and presented in panel A. Seven CpG sites including cg21091547 (CDKN1A), cg27306443 (PDE7B), cg19654743 (SEPT9), cg04751089 (IRS1), cg20995304 (HDAC7), cg01649611 (THADA) and cg15572489 (PTPRN2) were selected for technical validation of the Infinium HumanMethylation450 BeadChip data using pyrosequencing. Correlations between DNA methylation data analyzed with the two different methods were all significant and are shown in panel B. Correlations were calculated using Spearman's test. (TIF) [file pgen.1004160.s004.tif]

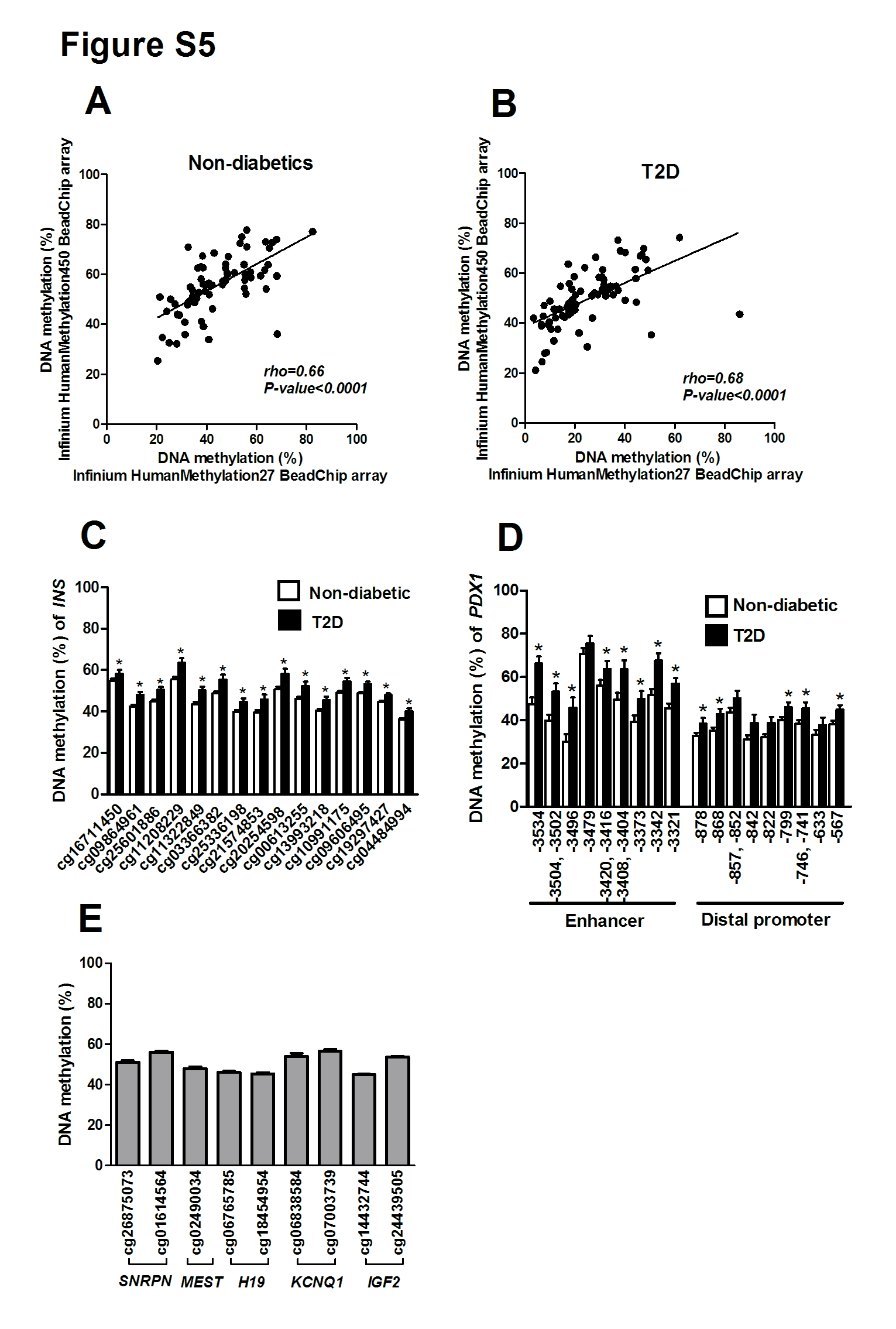

Supplement: Figure S5 — Confirmation of Infinium HumanMethylation450 BeadChip data. DNA methylation data of 264 CpG sites analyzed in human pancreatic islets from both our study and in the study by Volkmar et al correlate positively in (A) non-diabetic and (B) T2D donors. Increased DNA methylation of CpG sites in the INS and PDX1 genes in T2D versus non-diabetic islets (* P<0.05) is shown in panel C and D, respectively. The degree of DNA methylation of CpG sites in previously known imprinted genes in pancreatic islets of non-diabetic donors is shown in panel E. (TIF) [file pgen.1004160.s005.tif]
