# Supplementary material for: Genome-Wide DNA Methylation Analysis of Human Pancreatic Islets from Type 2 Diabetic and Non-Diabetic Donors Identifies Candidate Genes That Influence Insulin Secretion
Source: PLoS Genet. 2014 Mar 6;10(3):e1004160. doi: 10.1371/journal.pgen.1004160 (PMC3945174; doi:10.1371/journal.pgen.1004160)
Supplement: Table S2 — DMRs with differential DNA methylation (q<0.05 and difference in methylation ≥5%) in pancreatic islets from 34 non-diabetic versus 15 T2D human donors. (DOCX) [file pgen.1004160.s007.docx]

| **Table S2.** DMRs with differential DNA methylation (*q* < 0.05 and difference in methylation ≥ 5%) in pancreatic islets from 34 non-diabetic versus 15 T2D human donors. | | | | | | | | | | |
| --- | --- | --- | --- | --- | --- | --- | --- | --- | --- | --- |
| **Gene symbol** | **probe ID** | **DMR** | **Non-diabetic**  **DNA meth (%)**  **(mean ± Sd)** | **T2D DNA**  **meth (%)**  **(mean ± Sd)** | **Delta (%)** | ***P*-value** | ***q*-value** | **Chr.** | **Gene region** | **Relation to CpG island** |
| *AFF1* | cg11468363 | RDMR | 22.52 ± 6.17 | 14.86 ± 3.01 | -7.66 | 2.2 x 10-4 | 0.043 | 4 | 5'UTR | S Shore |
| *AFF1* | cg15148757 | RDMR | 19.33 ± 6.00 | 12.41 ± 2.70 | -6.92 | 1.5 x 10-4 | 0.037 | 4 | 5'UTR | S Shore |
| *ANKRD57* | cg22370326 | RDMR | 61.50 ± 6.25 | 55.71 ± 7.04 | -5.79 | 5.5 x 10-5 | 0.025 | 2 | 1stExon | S Shore |
| *ANO8* | cg24192660 | DMR | 36.04 ± 6.85 | 44.98 ± 7.11 | 8.94 | 3.0 x 10-4 | 0.049 | 19 | Body | Island |
| *ARL4C* | cg05204104 | DMR | 49.38 ± 10.82 | 40.44 ± 9.45 | -8.94 | 3.1 x 10-4 | 0.049 | 2 | 3'UTR;1stExon | N Shore |
| *ATP11A* | cg08464505 | CDMR | 47.02 ± 8.22 | 38.34 ± 6.29 | -8.67 | 5.6 x 10-5 | 0.025 | 13 | Body | S Shore |
| *B3GNT7* | cg06574960 | CDMR | 43.92 ± 7.71 | 32.55 ± 6.08 | -11.38 | 7.5 x 10-6 | 0.012 | 2 | 3'UTR | S Shore |
| *BCOR* | cg09368035 | CDMR | 50.63 ± 8.67 | 44.88 ± 5.99 | -5.75 | 4.8 x 10-5 | 0.023 | X | 5'UTR | Island |
| *BCOR* | cg00473051 | RDMR | 22.67 ± 6.05 | 15.00 ± 2.52 | -7.68 | 1.1 x 10-6 | 0.006 | X | 5'UTR | N Shore |
| *BCOR* | cg23701759 | RDMR | 51.18 ± 12.90 | 39.36 ± 9.66 | -11.82 | 1.2 x 10-4 | 0.034 | X | 5'UTR | Island |
| *BHLHE40* | cg16582517 | RDMR | 56.93 ± 6.47 | 47.37 ± 7.70 | -9.56 | 1.5 x 10-4 | 0.037 | 3 | 3'UTR | S Shore |
| *BICC1* | cg07567376 | RDMR | 75.62 ± 6.56 | 68.84 ± 6.79 | -6.78 | 1.3 x 10-4 | 0.034 | 10 | TSS1500 | N Shore |
| *C1orf106* | cg06854264 | CDMR | 62.11 ± 6.21 | 56.26 ± 6.12 | -5.84 | 2.4 x 10-5 | 0.018 | 1 | Body | S Shore |
| *C20orf3* | cg06937882 | RDMR | 28.87 ± 5.68 | 20.89 ± 3.77 | -7.98 | 7.2 x 10-5 | 0.028 | 20 | TSS1500 | S Shore |
| *C21orf84* | cg23732182 | DMR | 57.39 ± 7.15 | 50.96 ± 6.40 | -6.43 | 2.9 x 10-4 | 0.048 | 21 | Body |  |
| *CACNA2D3* | cg01559726 | RDMR | 18.36 ± 5.12 | 13.34 ± 2.21 | -5.02 | 9.4 x 10-5 | 0.030 | 3 | Body | S Shore |
| *CCDC40* | cg05159799 | DMR | 50.20 ± 6.63 | 41.99 ± 8.68 | -8.21 | 2.7 x 10-4 | 0.046 | 17 | Body | S Shelf |
| *CDKN1A* | cg03714916 | RDMR | 34.08 ± 5.28 | 24.84 ± 4.76 | -9.24 | 1.9 x 10-6 | 0.008 | 6 | TSS1500 | N Shore |
| *CDKN1A* | cg05460965 | RDMR | 55.69 ± 3.82 | 46.79 ± 3.71 | -8.90 | 3.2 x 10-7 | 0.004 | 6 | TSS1500 | N Shore |
| *CDKN1A* | cg15474579 | RDMR | 49.95 ± 7.01 | 39.09 ± 6.53 | -10.86 | 1.5 x 10-4 | 0.037 | 6 | TSS1500 | N Shore |
| *CDKN1A* | cg21091547 | RDMR | 69.21 ± 5.10 | 60.97 ± 5.70 | -8.25 | 3.7 x 10-6 | 0.009 | 6 | TSS1500 | N Shore |
| *CDKN1A* | cg24425727 | RDMR | 56.19 ± 8.03 | 44.47 ± 6.79 | -11.72 | 1.2 x 10-4 | 0.033 | 6 | TSS1500 | N Shore |
| *CELSR1* | cg22768487 | DMR | 60.42 ± 6.59 | 53.75 ± 6.43 | -6.67 | 1.0 x 10-4 | 0.032 | 22 | Body | N Shore |
| *CLMN* | cg02950354 | RDMR | 69.70 ± 6.00 | 63.23 ± 5.43 | -6.48 | 1.1 x 10-5 | 0.013 | 14 | Body | N Shore |
| *CMIP* | cg01425762 | DMR | 70.88 ± 5.84 | 64.92 ± 6.13 | -5.96 | 5.3 x 10-6 | 0.011 | 16 | Body |  |
| *CTNND2* | cg19438860 | DMR | 51.75 ± 5.71 | 43.75 ± 4.90 | -8.00 | 3.6 x 10-5 | 0.021 | 5 | Body | S Shore |
| *CYTSB* | cg20290367 | RDMR | 74.60 ± 4.56 | 69.37 ± 4.34 | -5.23 | 6.7 x 10-5 | 0.027 | 17 | Body | S Shore |
| *DEPDC5* | cg16160637 | RDMR | 31.64 ± 5.33 | 26.05 ± 3.75 | -5.58 | 1.1 x 10-4 | 0.033 | 22 | 1stExon;Body | S Shore |
| *DMTF1* | cg14881187 | RDMR | 50.43 ± 6.15 | 58.99 ± 6.02 | 8.56 | 1.6 x 10-4 | 0.037 | 7 | 5'UTR;Body | S Shelf |
| *EIF4E* | cg21341586 | RDMR | 53.02 ± 8.89 | 43.01 ± 9.99 | -10.01 | 9.6 x 10-6 | 0.013 | 4 | 5'UTR;1stExon; TSS1500 | S Shore |
| *ELFN1* | cg09160231 | DMR | 28.97 ± 4.02 | 23.26 ± 3.62 | -5.70 | 1.1 x 10-6 | 0.006 | 7 | 5'UTR |  |
| *FAM135B* | cg23712462 | RDMR | 52.91 ± 5.89 | 44.12 ± 5.79 | -8.79 | 2.1 x 10-6 | 0.008 | 8 | TSS1500 | S Shore |
| *FAM150B* | cg06905130 | RDMR | 41.56 ± 9.39 | 30.43 ± 6.99 | -11.14 | 2.7 x 10-4 | 0.046 | 2 | Body | N Shore |
| *FAS* | cg26478401 | RDMR | 76.75 ± 4.72 | 70.58 ± 6.59 | -6.18 | 1.0 x 10-4 | 0.031 | 10 | TSS1500;5'UTR | N Shore |
| *FEV* | cg23837867 | DMR | 41.75 ± 7.13 | 32.15 ± 5.69 | -9.60 | 3.0 x 10-4 | 0.049 | 2 | Body | Island |
| *FMOD* | cg03764585 | RDMR | 29.33 ± 5.66 | 22.24 ± 5.71 | -7.09 | 2.2 x 10-4 | 0.043 | 1 | 1stExon;5'UTR |  |
| *FSTL4* | cg23328852 | RDMR | 47.84 ± 5.83 | 41.78 ± 3.33 | -6.06 | 1.4 x 10-4 | 0.035 | 5 | 5'UTR | N Shore |
| *GIPC1* | cg27651355 | RDMR | 45.13 ± 6.92 | 37.75 ± 5.91 | -7.38 | 1.2 x 10-4 | 0.033 | 19 | TSS1500 | S Shore |
| *GPR124* | cg18715243 | RDMR | 55.10 ± 5.12 | 48.05 ± 3.69 | -7.05 | 1.3 x 10-4 | 0.034 | 8 | Body | S Shelf |
| *HBEGF* | cg15636295 | CDMR | 63.62 ± 6.81 | 56.85 ± 6.61 | -6.77 | 1.9 x 10-4 | 0.040 | 5 | Body | N Shore |
| *HHAT* | cg03893872 | CDMR | 57.61 ± 6.65 | 49.56 ± 6.52 | -8.04 | 1.3 x 10-4 | 0.034 | 1 | TSS1500;1stExon;5'UTR | N Shore |
| *HHAT* | cg22332066 | CDMR | 56.33 ± 6.02 | 48.62 ± 7.03 | -7.71 | 1.3 x 10-4 | 0.034 | 1 | TSS1500;1stExon;5'UTR | N Shore |
| *HHEX* | cg26979504 | RDMR | 44.33 ± 4.65 | 38.62 ± 5.44 | -5.71 | 2.6 x 10-4 | 0.046 | 10 | Body | Island |
| *HIC1* | cg13389502 | DMR | 49.81 ± 5.88 | 43.50 ± 5.91 | -6.31 | 1.5 x 10-4 | 0.037 | 17 | Body | Island |
| *IL20RA* | cg04071398 | CDMR | 77.06 ± 4.75 | 71.07 ± 6.50 | -5.99 | 5.4 x 10-8 | 0.003 | 6 | Body | N Shore |
| *IL6ST* | cg24729879 | RDMR | 40.32 ± 8.17 | 29.48 ± 6.67 | -10.83 | 6.3 x 10-5 | 0.026 | 5 | 5'UTR | N Shore |
| *INPP5A* | cg23023970 | DMR | 42.73 ± 6.84 | 34.78 ± 5.91 | -7.95 | 2.4 x 10-4 | 0.044 | 10 | Body |  |
| *IQSEC1* | cg09437283 | RDMR | 62.84 ± 5.76 | 55.86 ± 4.91 | -6.98 | 2.7 x 10-6 | 0.008 | 3 | Body | N Shore |
| *IRF8* | cg04599946 | CDMR | 63.34 ± 5.98 | 57.18 ± 8.36 | -6.16 | 1.3 x 10-4 | 0.034 | 16 | 5'UTR | S Shelf |
| *IRS1* | cg04751089 | RDMR | 40.02 ± 6.28 | 33.09 ± 4.40 | -6.93 | 5.5 x 10-5 | 0.025 | 2 | 3'UTR | N Shelf |
| *KCNJ5* | cg25508319 | DMR | 73.47 ± 4.47 | 68.47 ± 6.39 | -5.00 | 1.8 x 10-4 | 0.039 | 11 | 5'UTR |  |
| *KCNK5* | cg18705155 | CDMR | 67.85 ± 5.34 | 62.03 ± 5.52 | -5.82 | 1.0 x 10-4 | 0.031 | 6 | Body | N Shelf |
| *KCNQ1 KCNQ1QT1* | cg01693193 | RDMR | 72.51 ± 5.35 | 66.36 ± 5.69 | -6.15 | 4.6 x 10-5 | 0.023 | 11 | Body |  |
| *KLF6* | cg11847468 | RDMR | 59.21 ± 6.66 | 51.43 ± 6.93 | -7.79 | 5.0 x 10-5 | 0.024 | 10 | Body | N Shore |
| *KLF6* | cg14174912 | RDMR | 71.78 ± 6.55 | 63.44 ± 7.81 | -8.34 | 1.6 x 10-4 | 0.037 | 10 | Body | S Shore |
| *KLF6* | cg24003508 | RDMR | 54.37 ± 5.72 | 47.66 ± 5.71 | -6.71 | 1.0 x 10-5 | 0.013 | 10 | Body | S Shore |
| *KLF6* | cg24287110 | RDMR | 67.30 ± 7.39 | 57.86 ± 7.93 | -9.45 | 3.1 x 10-6 | 0.009 | 10 | Body | S Shore |
| *LPP* | cg16224163 | RDMR | 72.40 ± 5.50 | 65.34 ± 7.34 | -7.06 | 2.7 x 10-4 | 0.046 | 3 | TSS1500 | N Shore |
| *MAP3K5* | cg07474842 | DMR | 68.83 ± 4.75 | 63.64 ± 5.36 | -5.19 | 2.0 x 10-5 | 0.017 | 6 | Body |  |
| *MAP3K5* | cg27539060 | RDMR | 26.79 ± 5.63 | 17.84 ± 2.54 | -8.95 | 1.7 x 10-7 | 0.003 | 6 | Body | N Shore |
| *MED15* | cg27657537 | RDMR | 44.44 ± 4.97 | 37.82 ± 4.48 | -6.62 | 1.5 x 10-5 | 0.015 | 22 | Body | S Shore |
| *MLXIP* | cg13671582 | RDMR | 31.27 ± 5.32 | 23.78 ± 5.21 | -7.49 | 1.1 x 10-4 | 0.032 | 12 | Body | S Shelf |
| *MUM1* | cg02413187 | RDMR | 48.15 ± 7.79 | 39.14 ± 5.86 | -9.01 | 2.6 x 10-4 | 0.046 | 19 | TSS200;5'UTR | S Shore |
| *NAV2* | cg03220447 | DMR | 44.79 ± 5.42 | 37.23 ± 5.33 | -7.56 | 2.0 x 10-7 | 0.004 | 11 | Body |  |
| *NAV2* | cg04039799 | DMR | 42.96 ± 4.87 | 35.87 ± 5.07 | -7.09 | 6.3 x 10-7 | 0.005 | 11 | Body |  |
| *NAV2* | cg21052766 | DMR | 56.74 ± 5.01 | 48.53 ± 5.06 | -8.22 | 1.7 x 10-6 | 0.007 | 11 | Body |  |
| *NAV2* | cg13728131 | RDMR | 32.53 ± 6.68 | 23.53 ± 3.63 | -9.00 | 3.9 x 10-5 | 0.022 | 11 | Body | S Shore |
| *NCALD* | cg00680551 | RDMR | 61.76 ± 5.99 | 56.14 ± 6.23 | -5.62 | 1.0 x 10-4 | 0.031 | 8 | 5'UTR | N Shore |
| *NR2F2* | cg01213447 | RDMR | 64.57 ± 4.95 | 58.26 ± 5.86 | -6.30 | 9.9 x 10-5 | 0.031 | 15 | Body | N Shore |
| *NR2F2* | cg23734137 | RDMR | 66.19 ± 4.20 | 59.77 ± 4.10 | -6.42 | 1.1 x 10-6 | 0.006 | 15 | TSS1500 | S Shore |
| *P4HA2* | cg14527110 | RDMR | 30.71 ± 3.52 | 24.77 ± 2.82 | -5.95 | 1.1 x 10-4 | 0.032 | 5 | 5'UTR | N Shore |
| *PDGFB* | cg11714334 | DMR | 14.78 ± 4.99 | 8.52 ± 2.52 | -6.26 | 1.3 x 10-4 | 0.034 | 22 | Body;TSS1500 | N Shore |
| *PKDCC* | cg25353287 | RDMR | 69.51 ± 6.77 | 59.00 ± 8.52 | -10.51 | 1.8 x 10-4 | 0.039 | 2 | Body |  |
| *PLEC1* | cg16001422 | RDMR | 60.51 ± 6.63 | 55.03 ± 6.18 | -5.49 | 1.8 x 10-4 | 0.039 | 8 | Body | N Shore |
| *PLXNA1* | cg17552116 | RDMR | 68.36 ± 4.88 | 62.16 ± 6.14 | -6.20 | 6.4 x 10-5 | 0.026 | 3 | Body |  |
| *PRDM16* | cg08739115 | CDMR | 50.38 ± 7.70 | 42.58 ± 7.23 | -7.81 | 1.5 x 10-5 | 0.015 | 1 | Body |  |
| *PTPRN2* | cg17322774 | DMR | 61.78 ± 5.24 | 55.87 ± 5.19 | -5.91 | 1.3 x 10-5 | 0.014 | 7 | Body |  |
| *PTPRN2* | cg14832998 | RDMR | 52.34 ± 4.70 | 46.83 ± 4.62 | -5.51 | 2.7 x 10-4 | 0.047 | 7 | Body |  |
| *RAB11FIP1* | cg13625816 | RDMR | 59.43 ± 5.37 | 53.85 ± 6.25 | -5.58 | 2.0 x 10-4 | 0.041 | 8 | Body | N Shore |
| *RAB11FIP3* | cg03363565 | RDMR | 60.96 ± 7.05 | 52.21 ± 8.46 | -8.75 | 3.1 x 10-4 | 0.049 | 16 | TSS1500 | N Shore |
| *RAB11FIP3* | cg09555736 | RDMR | 73.77 ± 6.50 | 65.41 ± 8.13 | -8.35 | 9.7 x 10-5 | 0.031 | 16 | TSS1500 | N Shore |
| *RBM43* | cg05960677 | RDMR | 41.52 ± 6.36 | 31.95 ± 5.60 | -9.57 | 2.8 x 10-6 | 0.008 | 2 | Body | N Shore |
| *RGS6* | cg18515872 | DMR | 75.94 ± 4.29 | 69.70 ± 4.40 | -6.24 | 1.4 x 10-4 | 0.035 | 14 | Body |  |
| *RHOB* | cg25432336 | CDMR | 64.94 ± 6.06 | 56.40 ± 7.25 | -8.54 | 1.8 x 10-5 | 0.016 | 2 | 1stExon;3'UTR | S Shore |
| *RIMS2* | cg14750367 | DMR | 49.27 ± 5.74 | 43.21 ± 3.20 | -6.06 | 2.1 x 10-7 | 0.004 | 8 | Body |  |
| *RUNX1* | cg04212846 | RDMR | 49.00 ± 4.73 | 40.79 ± 4.75 | -8.21 | 3.7 x 10-7 | 0.004 | 21 | Body | S Shelf |
| *RUNX1* | cg19791221 | RDMR | 59.82 ± 4.02 | 53.84 ± 4.65 | -5.98 | 3.5 x 10-6 | 0.009 | 21 | Body | S Shelf |
| *SEMA5B* | cg19389001 | DMR | 36.46 ± 4.45 | 41.51 ± 5.94 | 5.05 | 1.2 x 10-4 | 0.033 | 3 | Body | Island |
| *SEMA5B* | cg26105015 | RDMR | 32.85 ± 6.30 | 25.70 ± 5.36 | -7.15 | 6.9 x 10-5 | 0.027 | 3 | 5'UTR | N Shore |
| *SFXN3* | cg15428620 | RDMR | 62.13 ± 5.45 | 56.28 ± 5.41 | -5.85 | 1.1 x 10-4 | 0.032 | 10 | Body | S Shore |
| *SGK1* | cg14905466 | RDMR | 43.02 ± 6.01 | 35.68 ± 5.66 | -7.34 | 2.0 x 10-4 | 0.041 | 6 | TSS200;Body | S Shore |
| *SH3GL1* | cg08418670 | RDMR | 87.00 ± 4.25 | 82.01 ± 6.06 | -4.99 | 5.7 x 10-5 | 0.025 | 19 | Body | S Shelf |
| *SIX2* | cg17272224 | CDMR | 45.54 ± 4.35 | 39.83 ± 2.33 | -5.71 | 1.1 x 10-5 | 0.014 | 2 | TSS1500 | S Shore |
| *SIX2* | cg02711647 | DMR | 25.71 ± 6.30 | 17.96 ± 4.68 | -7.74 | 1.1 x 10-4 | 0.032 | 2 | TSS1500 | Island |
| *SIX2* | cg02893453 | RDMR | 55.71 ± 4.83 | 50.68 ± 3.34 | -5.03 | 5.8 x 10-6 | 0.011 | 2 | 3'UTR | S Shore |
| *SLC17A6* | cg20809470 | DMR | 24.60 ± 5.46 | 18.82 ± 3.51 | -5.79 | 3.1 x 10-4 | 0.049 | 11 | Body | S Shore |
| *SLC1A5* | cg03359362 | RDMR | 68.47 ± 6.53 | 59.97 ± 8.56 | -8.50 | 2.2 x 10-4 | 0.043 | 19 | TSS1500;Body;  5'UTR | N Shore |
| *SLC41A1* | cg10717869 | RDMR | 23.12 ± 4.14 | 17.29 ± 3.13 | -5.83 | 1.2 x 10-4 | 0.033 | 1 | 5'UTR | N Shore |
| *SLC7A5* | cg00858400 | RDMR | 76.58 ± 6.41 | 69.40 ± 7.23 | -7.18 | 6.0 x 10-5 | 0.026 | 16 | TSS1500 | S Shore |
| *SLC9A3R2* | cg08074555 | CDMR | 71.55 ± 4.90 | 65.75 ± 5.81 | -5.80 | 2.4 x 10-4 | 0.045 | 16 | Body | N Shore |
| *SPSB1* | cg26352795 | RDMR | 49.64 ± 9.12 | 39.30 ± 6.42 | -10.34 | 1.5 x 10-5 | 0.015 | 1 | 5'UTR |  |
| *SVIL* | cg06197966 | DMR | 64.52 ± 6.18 | 57.38 ± 6.37 | -7.14 | 4.1 x 10-5 | 0.022 | 10 | 5'UTR |  |
| *SVIL* | cg13324103 | DMR | 68.78 ± 5.94 | 62.54 ± 7.24 | -6.24 | 2.6 x 10-5 | 0.018 | 10 | 5'UTR |  |
| *TACC1* | cg27460040 | RDMR | 70.83 ± 4.22 | 64.26 ± 4.48 | -6.57 | 2.9 x 10-4 | 0.048 | 8 | Body;5'UTR | S Shelf |
| *TBC1D16* | cg25278144 | CDMR | 65.77 ± 5.37 | 59.88 ± 4.71 | -5.88 | 6.0 x 10-7 | 0.005 | 17 | Body | N Shelf |
| *TGFBR3* | cg23721586 | DMR | 54.62 ± 8.66 | 43.84 ± 10.81 | -10.78 | 6.9 x 10-5 | 0.027 | 1 | Body |  |
| *TPM4* | cg06590173 | RDMR | 68.64 ± 5.84 | 61.74 ± 6.16 | -6.90 | 5.4 x 10-5 | 0.025 | 19 | Body | S Shore |
| *TPM4* | cg27377863 | RDMR | 31.38 ± 7.08 | 21.35 ± 4.85 | -10.03 | 5.3 x 10-6 | 0.011 | 19 | Body | S Shore |
| *TPPP* | cg04230438 | RDMR | 45.49 ± 4.54 | 39.74 ± 4.37 | -5.75 | 1.5 x 10-4 | 0.036 | 5 | Body | S Shelf |
| *TSPAN18* | cg20968743 | DMR | 66.94 ± 6.02 | 60.75 ± 7.33 | -6.19 | 4.4 x 10-6 | 0.010 | 11 | 5'UTR |  |
| *TTC28* | cg21652122 | RDMR | 75.93 ± 5.14 | 70.74 ± 5.89 | -5.19 | 2.5 x 10-4 | 0.045 | 22 | Body |  |
| *VEGFA* | cg01298514 | RDMR | 80.65 ± 3.69 | 74.93 ± 4.99 | -5.72 | 3.7 x 10-5 | 0.021 | 6 | TSS1500 | N Shore |
| *WIPF1* | cg13651986 | DMR | 49.85 ± 6.00 | 43.97 ± 4.17 | -5.88 | 6.5 x 10-5 | 0.027 | 2 | TSS1500 | S Shore |
| *WNT16* | cg03721528 | CDMR | 61.59 ± 6.40 | 55.41 ± 6.56 | -6.18 | 1.9 x 10-4 | 0.040 | 7 | TSS1500;Body | N Shore |
| *ZC3H12A* | cg03140118 | RDMR | 30.65 ± 5.00 | 23.97 ± 5.51 | -6.67 | 8.6 x 10-5 | 0.029 | 1 | TSS1500 | N Shore |
| *ZNF516* | cg15347788 | RDMR | 68.38 ± 5.58 | 59.43 ± 7.17 | -8.95 | 3.0 x 10-7 | 0.004 | 18 | 5'UTR | S Shore |
| *ZNF703* | cg25487404 | RDMR | 33.95 ± 7.04 | 23.27 ± 6.43 | -10.67 | 1.3 x 10-4 | 0.034 | 8 | TSS1500 | Island |
|  | cg01582438 | CDMR | 23.27 ± 4.30 | 18.09 ± 1.68 | -5.18 | 3.7 x 10-7 | 0.004 | 20 |  | N Shore |
|  | cg12758090 | CDMR | 42.47 ± 5.55 | 33.46 ± 5.42 | -9.01 | 2.9 x 10-4 | 0.048 | 15 |  | N Shore |
|  | cg12950645 | CDMR | 69.92 ± 7.59 | 63.92 ± 7.55 | -6.00 | 1.5 x 10-4 | 0.036 | 15 |  | S Shelf |
|  | cg15808063 | CDMR | 36.15 ± 5.19 | 29.03 ± 4.31 | -7.12 | 9.0 x 10-5 | 0.030 | 2 |  | N Shelf |
|  | cg16131053 | CDMR | 74.40 ± 5.38 | 67.73 ± 7.58 | -6.67 | 9.1 x 10-5 | 0.030 | 18 |  | S Shore |
|  | cg26401166 | CDMR | 75.04 ± 6.95 | 69.76 ± 6.11 | -5.28 | 1.0 x 10-5 | 0.013 | 15 |  | S Shore |
|  | cg02560388 | DMR | 34.21 ± 4.53 | 27.88 ± 4.65 | -6.33 | 6.5 x 10-5 | 0.027 | 2 |  |  |
|  | cg02788637 | DMR | 36.14 ± 7.26 | 27.75 ± 8.30 | -8.38 | 2.9 x 10-4 | 0.048 | 7 |  | Island |
|  | cg04192867 | DMR | 30.65 ± 4.19 | 23.89 ± 2.75 | -6.76 | 1.3 x 10-6 | 0.006 | 10 |  | Island |
|  | cg04499701 | DMR | 75.80 ± 5.78 | 70.53 ± 6.61 | -5.26 | 7.0 x 10-5 | 0.027 | 11 |  |  |
|  | cg05267204 | DMR | 71.50 ± 5.64 | 64.74 ± 6.69 | -6.76 | 3.8 x 10-6 | 0.009 | 2 |  |  |
|  | cg05368966 | DMR | 37.67 ± 4.90 | 32.30 ± 3.37 | -5.37 | 4.8 x 10-5 | 0.023 | 12 |  |  |
|  | cg06213900 | DMR | 58.34 ± 6.47 | 49.35 ± 7.48 | -8.99 | 1.7 x 10-4 | 0.039 | X |  |  |
|  | cg08627825 | DMR | 52.08 ± 7.39 | 44.33 ± 3.51 | -7.75 | 2.6 x 10-6 | 0.008 | 22 |  | Island |
|  | cg08745334 | DMR | 74.93 ± 5.79 | 69.66 ± 6.17 | -5.27 | 2.1 x 10-4 | 0.042 | 4 |  |  |
|  | cg10763234 | DMR | 28.04 ± 8.36 | 34.04 ± 9.46 | 6.00 | 1.2 x 10-4 | 0.033 | 15 |  | Island |
|  | cg12725760 | DMR | 36.97 ± 7.25 | 28.45 ± 4.57 | -8.52 | 5.7 x 10-6 | 0.011 | 1 |  |  |
|  | cg15275625 | DMR | 54.51 ± 4.80 | 47.05 ± 4.20 | -7.46 | 1.8 x 10-8 | 0.003 | 3 |  |  |
|  | cg17207552 | DMR | 58.15 ± 9.54 | 51.46 ± 6.65 | -6.70 | 2.0 x 10-4 | 0.041 | X |  |  |
|  | cg19924948 | DMR | 52.85 ± 7.63 | 42.73 ± 8.64 | -10.11 | 1.6 x 10-4 | 0.037 | 2 |  |  |
|  | cg20780998 | DMR | 53.56 ± 10.25 | 41.67 ± 9.22 | -11.89 | 2.7 x 10-4 | 0.046 | X |  | S Shelf |
|  | cg22246636 | DMR | 45.96 ± 4.12 | 40.90 ± 3.54 | -5.06 | 1.0 x 10-4 | 0.031 | 20 |  | Island |
|  | cg24035164 | DMR | 26.82 ± 4.58 | 21.64 ± 2.39 | -5.18 | 8.5 x 10-5 | 0.029 | 10 |  | N Shore |
|  | cg26140475 | DMR | 27.55 ± 4.89 | 21.11 ± 4.36 | -6.45 | 5.7 x 10-5 | 0.025 | 8 |  |  |
|  | cg26476925 | DMR | 41.13 ± 7.13 | 31.92 ± 7.12 | -9.22 | 1.2 x 10-4 | 0.033 | 19 |  |  |
|  | cg27097542 | DMR | 62.37 ± 7.22 | 55.36 ± 6.35 | -7.01 | 7.4 x 10-5 | 0.028 | 16 |  |  |
|  | cg00510870 | RDMR | 32.71 ± 4.20 | 26.49 ± 3.68 | -6.21 | 3.1 x 10-5 | 0.020 | 8 |  | Island |
|  | cg00732383 | RDMR | 61.70 ± 5.28 | 56.01 ± 5.49 | -5.70 | 1.1 x 10-5 | 0.014 | 18 |  | S Shelf |
|  | cg01601949 | RDMR | 46.54 ± 5.42 | 40.28 ± 5.20 | -6.26 | 1.0 x 10-4 | 0.031 | 2 |  | Island |
|  | cg01635555 | RDMR | 60.40 ± 6.95 | 53.79 ± 8.26 | -6.61 | 2.6 x 10-4 | 0.046 | 16 |  | S Shore |
|  | cg02574734 | RDMR | 61.63 ± 4.29 | 55.86 ± 3.89 | -5.77 | 2.6 x 10-7 | 0.004 | 1 |  | N Shore |
|  | cg02924943 | RDMR | 29.64 ± 4.05 | 22.91 ± 3.26 | -6.74 | 3.6 x 10-7 | 0.004 | 3 |  | N Shore |
|  | cg07392324 | RDMR | 46.88 ± 6.69 | 40.12 ± 4.10 | -6.76 | 5.9 x 10-5 | 0.025 | 7 |  | S Shore |
|  | cg09452082 | RDMR | 78.01 ± 5.80 | 71.09 ± 7.91 | -6.91 | 2.2 x 10-4 | 0.043 | 15 |  | N Shore |
|  | cg12568786 | RDMR | 53.00 ± 6.30 | 47.86 ± 5.21 | -5.13 | 7.6 x 10-5 | 0.028 | 5 |  | N Shelf |
|  | cg15392844 | RDMR | 52.11 ± 6.11 | 44.71 ± 5.75 | -7.40 | 1.2 x 10-4 | 0.033 | 1 |  | N Shore |
|  | cg19239199 | RDMR | 69.36 ± 4.93 | 62.16 ± 6.21 | -7.21 | 9.6 x 10-7 | 0.006 | 7 |  | N Shore |
|  | cg19618897 | RDMR | 60.04 ± 7.21 | 50.05 ± 7.36 | -9.99 | 1.3 x 10-4 | 0.034 | 17 |  | S Shore |
|  | cg20477147 | RDMR | 50.28 ± 5.90 | 42.87 ± 5.72 | -7.41 | 1.9 x 10-4 | 0.040 | 20 |  | N Shore |
|  | cg21481966 | RDMR | 66.08 ± 6.09 | 59.02 ± 5.67 | -7.06 | 2.1 x 10-5 | 0.017 | 2 |  | N Shelf |
|  | cg23233742 | RDMR | 35.30 ± 4.63 | 27.84 ± 3.71 | -7.45 | 2.1 x 10-5 | 0.017 | 5 |  | S Shore |
|  | cg25403283 | RDMR | 76.22 ± 6.75 | 68.31 ± 7.88 | -7.91 | 7.5 x 10-5 | 0.028 | 10 |  |  |
|  | cg26794871 | RDMR | 51.19 ± 6.03 | 44.06 ± 5.37 | -7.13 | 8.2 x 10-5 | 0.029 | 20 |  | N Shore |

Differentially methylated regions (DMR); cancer specific differentially methylated regions (cDMR); reprogramming specific differentially methylated regions (rDMR)
