# Supplementary material for: Genome-Wide DNA Methylation Analysis of Human Pancreatic Islets from Type 2 Diabetic and Non-Diabetic Donors Identifies Candidate Genes That Influence Insulin Secretion
Source: PLoS Genet. 2014 Mar 6;10(3):e1004160. doi: 10.1371/journal.pgen.1004160 (PMC3945174; doi:10.1371/journal.pgen.1004160)
Supplement: Table S3 — Non-CpG DNA methylation in human pancreatic islets. (DOCX) [file pgen.1004160.s008.docx]

**Table S3.** Non-CpG DNA methylation in human pancreatic islets.

| **Sequence** | **Number of sites on chip (%)** | **Average DNA methylation (%)** |
| --- | --- | --- |
| [CA]G | 1,040 (87.5) | 13.5 ± 5.9 |
| [CA]A | 83 (7.0) | 11.6 ± 5.0 |
| [CA]T | 6 (0.5) | 18.0 ± 8.5 |
| [CA]C | 55 (4.6) | 21.4 ± 10.8 |
| [CT]G | 5 (0.4) | 5.2 ± 1.3 |
| **Total** | **1,189** |  |
