# Supplementary material for: Genome-Wide DNA Methylation Analysis of Human Pancreatic Islets from Type 2 Diabetic and Non-Diabetic Donors Identifies Candidate Genes That Influence Insulin Secretion
Source: PLoS Genet. 2014 Mar 6;10(3):e1004160. doi: 10.1371/journal.pgen.1004160 (PMC3945174; doi:10.1371/journal.pgen.1004160)
Supplement: Table S5 — CpG sites that exhibit differential DNA methylation in pancreatic islets from T2D compared with non-diabetic islets in the study by Volkmar et al as well as in the present study with P<0.05. DNA methylation data from the 34 non-diabetic and 15 T2D human donors analyzed in the present study is presented in this table. (DOCX) [file pgen.1004160.s010.docx]

**Table S5.** CpG sites that exhibit differential DNA methylation in pancreatic islets from T2D compared with non-diabetic islets in the study by Volkmar *et al* as well as in the present study with *P* < 0.05. DNA methylation data from the 34 non-diabetic and 15 T2D human donors analyzed in the present study is presented in this table.

| **Gene symbol** | **Probe ID** | **Non-diabetic**  **DNA meth**  **(%)** | **T2D**  **DNA meth**  **(%)** | **Delta**  **DNA meth**  **(%)** | ***P*-value** | **Gene region** | **Relation to**  **CpG island** |
| --- | --- | --- | --- | --- | --- | --- | --- |
| *ACSF2* | cg14672994 | 32.58 ± 5.77 | 27.90 ± 6.73 | -4.68 | 8.6 x 10^-3^ | TSS1500 |  |
| *ALDH3B1* | cg07730301 | 53.84 ± 6.89 | 47.06 ± 7.51 | -6.79 | 1.8 x 10^-2^ | 5'UTR |  |
| *ALDH3B1* | cg15322932 | 50.07 ± 8.32 | 42.80 ± 8.05 | -7.27 | 8.7 x 10^-3^ | TSS2005'UTR |  |
| *ANPEP* | cg05985767 | 54.40 ± 4.85 | 51.37 ± 4.54 | -3.03 | 4.0 x 10^-2^ | Body |  |
| *APOH* | cg19058765 | 77.77 ± 6.90 | 73.26 ± 7.07 | -4.51 | 4.9 x 10^-2^ | TSS1500 |  |
| *ARHGEF19* | cg18669381 | 43.97 ± 5.18 | 39.34 ± 4.62 | -4.64 | 1.5 x 10^-2^ | 5'UTR;1stExon |  |
| *B3GALT5* | cg11479877 | 70.85 ± 9.66 | 63.55 ± 11.04 | -7.30 | 5.0 x 10^-3^ | 1stExon;5'UTR |  |
| *C20orf160* | cg24691461 | 51.16 ± 6.06 | 45.57 ± 7.61 | -5.59 | 3.8 x 10^-2^ | Body |  |
| *C20orf166;C20orf200* | cg11682508 | 57.66 ± 6.91 | 50.89 ± 8.00 | -6.77 | 2.7 x 10^-2^ | 5'UTR;TSS1500 | N Shore |
| *C21orf84* | cg23732182 | 57.39 ± 7.15 | 50.96 ± 6.40 | -6.43 | 2.9 x 10^-4^ | Body |  |
| *C3orf18* | cg23320649 | 47.84 ± 6.04 | 42.37 ± 6.61 | -5.47 | 4.5 x 10^-3^ | 5'UTR | N Shore |
| *C4BPB* | cg05659947 | 67.37 ± 11.12 | 58.62 ± 11.62 | -8.75 | 1.1 x 10^-2^ | TSS1500 |  |
| *CDCA7L* | cg13316191 | 50.88 ± 8.39 | 41.99 ± 10.06 | -8.89 | 1.3 x 10^-2^ | TSS1500 | S Shore |
| *CDCP2* | cg20777437 | 55.75 ± 8.57 | 47.81 ± 9.06 | -7.94 | 2.6 x 10^-3^ | TSS1500 |  |
| *CDK5R1* | cg04106785 | 63.90 ± 6.10 | 58.44 ± 5.98 | -5.47 | 4.3 x 10^-2^ | TSS1500 | N Shore |
| *CHST4* | cg05535113 | 75.04 ± 6.28 | 68.94 ± 8.20 | -6.10 | 6.8 x 10^-3^ | TSS1500 |  |
| *CPA1* | cg15154229 | 57.91 ± 6.31 | 53.30 ± 4.45 | -4.61 | 1.6 x 10^-2^ | TSS1500 | N Shore |
| *CTTNBP2* | cg21880328 | 48.75 ± 7.09 | 44.41 ± 6.22 | -4.34 | 4.8 x 10^-2^ | TSS1500 | S Shore |
| *CXCL17* | cg15937958 | 72.78 ± 7.90 | 66.87 ± 7.91 | -5.91 | 1.9 x 10^-2^ | 1stExon;5'UTR | N Shore |
| *CYP4F12* | cg05722906 | 56.14 ± 9.13 | 47.65 ± 10.85 | -8.49 | 3.1 x 10^-3^ | TSS200 |  |
| *CYSLTR1* | cg26848126 | 67.15 ± 10.50 | 61.40 ± 7.67 | -5.75 | 1.7 x 10^-3^ | 5'UTR;1stExon |  |
| *DDO* | cg20011134 | 62.69 ± 8.15 | 52.81 ± 9.52 | -9.87 | 5.2 x 10^-4^ | Body |  |
| *DDX52* | cg02992647 | 63.04 ± 9.72 | 54.79 ± 10.54 | -8.25 | 1.2 x 10^-2^ | TSS1500 | S Shore |
| *DRD2* | cg21330703 | 43.68 ± 5.56 | 37.57 ± 5.89 | -6.11 | 1.6 x 10^-2^ | TSS1500 | S Shore |
| *FGG* | cg01593385 | 62.51 ± 5.95 | 57.47 ± 6.90 | -5.03 | 2.9 x 10^-3^ | TSS1500 |  |
| *GABRB3* | cg15940569 | 68.54 ± 7.34 | 62.16 ± 8.91 | -6.38 | 2.6 x 10^-2^ | TSS1500 |  |
| *GIT1* | cg05379350 | 60.56 ± 4.70 | 55.32 ± 7.21 | -5.24 | 4.3 x 10^-2^ | TSS1500 | N Shore |
| *GPR152* | cg11504740 | 60.91 ± 7.45 | 53.20 ± 7.35 | -7.70 | 1.6 x 10^-3^ | 1stExon |  |
| *GSTP1* | cg11566244 | 52.67 ± 10.22 | 43.32 ± 12.04 | -9.35 | 6.8 x 10^-3^ | Body | Island |
| *GSTP1* | cg22224704 | 48.73 ± 7.90 | 40.52 ± 8.15 | -8.21 | 3.9 x 10^-3^ | Body | S Shore |
| *GUCA2A* | cg06003187 | 73.94 ± 4.69 | 69.87 ± 5.55 | -4.07 | 3.2 x 10^-2^ | Body |  |
| *GUCA2B* | cg17100200 | 77.17 ± 5.52 | 74.19 ± 6.22 | -2.98 | 7.1 x 10^-3^ | TSS1500 |  |
| *IGF2AS* | cg12322132 | 48.12 ± 6.42 | 39.56 ± 5.74 | -8.55 | 1.7 x 10^-3^ | Body;5'UTR | Island |
| *IGF2AS;INS-IGF2* | cg20792294 | 41.14 ± 6.50 | 32.92 ± 6.83 | -8.22 | 3.7 x 10^-3^ | Body;5'UTR | Island |
| *INMT* | cg04749372 | 54.20 ± 6.37 | 48.30 ± 7.51 | -5.89 | 1.0 x 10^-2^ | 1stExon |  |
| *ITIH4* | cg17890764 | 62.68 ± 8.80 | 55.79 ± 11.22 | -6.89 | 2.5 x 10^-2^ | TSS200 |  |
| *ITIH4* | cg10929387 | 58.72 ± 5.21 | 54.27 ± 5.72 | -4.46 | 4.4 x 10^-3^ | 5'UTR;1stExon |  |
| *LGALS2* | cg11081833 | 62.60 ± 10.13 | 53.61 ± 9.77 | -8.99 | 1.2 x 10^-2^ | 1stExon;5'UTR |  |
| *LYL1* | cg15013019 | 33.92 ± 3.43 | 30.51 ± 3.72 | -3.40 | 1.3 x 10^-2^ | 5'UTR;1stExon | Island |
| *MED13L* | cg12113819 | 39.10 ± 4.39 | 36.09 ± 3.88 | -3.01 | 3.0 x 10^-2^ | TSS1500 |  |
| *MUC1* | cg24512973 | 72.47 ± 6.28 | 66.36 ± 7.79 | -6.11 | 3.3 x 10^-3^ | Body | N Shore |
| *NCRNA00175;COL18A1* | cg05275605 | 63.88 ± 4.27 | 61.11 ± 5.19 | -2.76 | 2.4 x 10^-2^ | Body |  |
| *NFAM1* | cg17568996 | 55.86 ± 8.92 | 47.40 ± 11.58 | -8.46 | 1.4 x 10^-2^ | Body |  |
| *OSTalpha* | cg05473677 | 40.80 ± 4.24 | 37.62 ± 4.52 | -3.18 | 3.0 x 10^-2^ | Body |  |
| *P2RY2* | cg10287137 | 60.00 ± 5.09 | 54.75 ± 5.31 | -5.26 | 1.6 x 10^-2^ | TSS1500 | Island |
| *PAQR7* | cg15662251 | 57.23 ± 7.04 | 51.38 ± 7.83 | -5.85 | 4.5 x 10^-2^ | TSS200 | N Shelf |
| *PCK1* | cg16101800 | 70.57 ± 5.79 | 65.46 ± 7.45 | -5.11 | 2.8 x 10^-2^ | Body |  |
| *PLSCR4* | cg24315815 | 58.15 ± 10.34 | 48.79 ± 11.09 | -9.36 | 1.1 x 10^-2^ | TSS1500 | S Shore |
| *PWWP2B* | cg00259755 | 25.37 ± 6.38 | 21.22 ± 6.05 | -4.15 | 3.9 x 10^-2^ | TSS1500 | Island |
| *PYY;NAGS* | cg09467501 | 32.19 ± 5.11 | 28.30 ± 4.17 | -3.89 | 3.3 x 10^-2^ | TSS1500;Body | Island |
| *REG1A* | cg05828624 | 72.99 ± 5.87 | 67.36 ± 8.17 | -5.63 | 4.9 x 10^-2^ | 5'UTR |  |
| *RHOD* | cg12473775 | 56.40 ± 8.54 | 51.28 ± 8.31 | -5.12 | 4.3 x 10^-2^ | Body | S Shore |
| *RILP* | cg04049033 | 60.68 ± 6.15 | 54.80 ± 7.14 | -5.88 | 1.9 x 10^-2^ | TSS1500 | S Shore |
| *RNU5E;RNU5D;CKMT2* | cg10978355 | 34.72 ± 7.74 | 24.52 ± 6.17 | -10.20 | 7.4 x 10^-4^ | Body;5'UTR |  |
| *SAMD11* | cg14324200 | 45.24 ± 5.60 | 38.84 ± 4.81 | -6.40 | 4.7 x 10^-3^ | 5'UTR | Island |
| *SCNN1D* | cg13587552 | 52.19 ± 3.78 | 49.14 ± 4.36 | -3.05 | 2.1 x 10^-2^ | TSS1500 |  |
| *SDCBP2* | cg16173067 | 64.08 ± 7.27 | 58.37 ± 7.17 | -5.71 | 1.4 x 10^-3^ | 5'UTR | S Shelf |
| *SDPR* | cg06352750 | 54.94 ± 8.99 | 45.58 ± 10.14 | -9.36 | 5.2 x 10^-3^ | 5'UTR;1stExon |  |
| *SHISA4* | cg26203861 | 46.20 ± 3.85 | 42.10 ± 3.49 | -4.10 | 9.6 x 10^-4^ | Body |  |
| *SLC17A4* | cg15916061 | 51.89 ± 5.43 | 46.39 ± 6.16 | -5.50 | 9.0 x 10^-4^ | TSS1500 |  |
| *SLC39A12* | cg19856444 | 70.99 ± 3.57 | 68.28 ± 5.85 | -2.72 | 4.3 x 10^-2^ | 5'UTR |  |
| *SLC39A5* | cg00668685 | 59.45 ± 7.32 | 53.17 ± 7.20 | -6.28 | 2.5 x 10^-3^ | 1stExon;5'UTR |  |
| *SNX33* | cg27635271 | 55.61 ± 8.53 | 49.20 ± 8.72 | -6.41 | 3.7 x 10^-2^ | 1stExon | S Shore |
| *SULT1A2* | cg00931491 | 49.18 ± 6.95 | 42.15 ± 7.21 | -7.03 | 8.1 x 10^-3^ | 5'UTR;TSS1500 | N Shelf |
| *SYNC* | cg05342835 | 53.18 ± 7.32 | 46.67 ± 9.89 | -6.52 | 9.3 x 10^-3^ | Body |  |
| *TBC1D9B* | cg10737521 | 55.41 ± 8.60 | 44.19 ± 10.94 | -11.22 | 3.4 x 10^-4^ | TSS1500 | S Shore |
| *TFF2* | cg12456510 | 61.69 ± 5.52 | 57.85 ± 5.41 | -3.84 | 3.1 x 10^-2^ | TSS200 |  |
| *TMEM8A;MRPL28* | cg10805676 | 36.06 ± 8.08 | 43.53 ± 11.35 | 7.47 | 3.0 x 10^-2^ | Body;TSS1500 | N Shore |
| *TRPV6* | cg16752583 | 50.49 ± 6.06 | 45.31 ± 6.38 | -5.18 | 4.5 x 10^-3^ | TSS1500 |  |
| *UNKL* | cg26728422 | 60.11 ± 9.23 | 52.05 ± 9.91 | -8.05 | 5.7 x 10^-3^ | 5'UTR | N Shore |
| *VTN* | cg21846903 | 49.26 ± 6.13 | 42.97 ± 5.98 | -6.29 | 7.5 x 10^-4^ | 1stExon;5'UTR | N Shore |
