# Supplementary material for: Genome-Wide DNA Methylation Analysis of Human Pancreatic Islets from Type 2 Diabetic and Non-Diabetic Donors Identifies Candidate Genes That Influence Insulin Secretion
Source: PLoS Genet. 2014 Mar 6;10(3):e1004160. doi: 10.1371/journal.pgen.1004160 (PMC3945174; doi:10.1371/journal.pgen.1004160)
Supplement: Table S6 — Characteristics of 87 non-diabetic human donors of pancreatic islets used to examine the impact of HbA1c, age and BMI on DNA methylation of the 1,649 CpG that exhibit differential DNA methylation in pancreatic islets from 34 non-diabetic versus 15 T2D human donors. (DOCX) [file pgen.1004160.s011.docx]

| **Table S6.** Characteristics of 87 non-diabetic human donors of pancreatic islets used to examine the impact of HbA1c, age and BMI on DNA methylation of the 1,649 CpG that exhibit differential DNA methylation in pancreatic islets from 34 non-diabetic versus 15 T2D human donors. | | | |
| --- | --- | --- | --- |
|  | **Mean ± SD** | **Range (min - max)** | |
| Gender (Males/Females) | (53/34) |  | |
| HbA1c (%) | 5.6 ± 0.4 | 4.3 - 6.4 | |
| Age (years) | 56.7 ± 10.5 | 26 - 74 | |
| BMI (kg/m^2^) | 25.8 ± 3.4 | 17.6 - 40.1 | |
