# Supplementary material for: Genome-Wide DNA Methylation Analysis of Human Pancreatic Islets from Type 2 Diabetic and Non-Diabetic Donors Identifies Candidate Genes That Influence Insulin Secretion
Source: PLoS Genet. 2014 Mar 6;10(3):e1004160. doi: 10.1371/journal.pgen.1004160 (PMC3945174; doi:10.1371/journal.pgen.1004160)
Supplement: Table S7 — CpG sites that exhibit differential DNA methylation (q<0.05 and difference in methylation ≥5%) in pancreatic islets from 34 non-diabetic versus 15 T2D human donors in parallel with an association between HbA1c levels and differential DNA methylation (P<0.05) in pancreatic islets from 87 non-diabetic donors. (DOCX) [file pgen.1004160.s012.docx]

**Table S7.** CpG sites that exhibit differential DNA methylation (*q* < 0.05 and difference in methylation ≥ 5%) in pancreatic islets from 34 non-diabetic versus 15 T2D human donors in parallel with an association between HbA1c levels and differential DNA methylation (*P* < 0.05) in pancreatic islets from 87 non-diabetic donors.

| **Gene Symbol** | **Probe ID** | **Non-diabetic**  **DNA meth (%)**  **(mean±Sd)** | **T2D**  **DNA meth (%)**  **(mean±Sd)** | **Delta**  **DNA meth**  **(%)** | ***P*-value** | **q-value** | **Association between HbA1c and DNA meth** | | | **Chr.** | **Gene region** | **Relation to CpG island** |
| --- | --- | --- | --- | --- | --- | --- | --- | --- | --- | --- | --- | --- |
|  |  |  |  |  |  |  | **beta-coef.** | **Sem** | ***P*-value** |  |  |  |
| *ADAMTS12* | cg09747891 | 36.72 ± 7.72 | 27.48 ± 6.71 | -9.2 | 2.7 x 10-4 | 0.047 | -0.040 | 0.020 | 0.049 | 5 | TSS1500 |  |
| *AFF1* | cg11468363 | 22.52 ± 6.17 | 14.86 ± 3.01 | -7.7 | 2.2 x 10-4 | 0.043 | -0.052 | 0.023 | 0.024 | 4 | 5'UTR |  |
| *ANKRD57* | cg22370326 | 61.50 ± 6.25 | 55.71 ± 7.04 | -5.8 | 5.5 x 10-5 | 0.025 | -0.035 | 0.017 | 0.037 | 2 | 1stExon |  |
| *ANXA11* | cg02291020 | 29.90 ± 7.41 | 21.98 ± 4.72 | -7.9 | 4.2 x 10-5 | 0.022 | -0.056 | 0.025 | 0.024 | 10 | 5'UTR |  |
| *ATXN7L1* | cg10575219 | 44.16 ± 8.08 | 34.51 ± 4.70 | -9.7 | 1.2 x 10-4 | 0.034 | -0.059 | 0.021 | 0.005 | 7 | Body;5'UTR |  |
| *AXIN2* | cg23475474 | 36.54 ± 4.84 | 29.30 ± 3.76 | -7.2 | 1.1 x 10-5 | 0.014 | -0.029 | 0.014 | 0.031 | 17 | Body |  |
| *BCL9L* | cg02493211 | 35.26 ± 6.19 | 24.67 ± 5.67 | -10.6 | 7.2 x 10-5 | 0.028 | -0.037 | 0.019 | 0.049 | 11 | TSS200 |  |
| *BTBD17* | cg00898111 | 24.47 ± 4.97 | 18.79 ± 3.15 | -5.7 | 1.0 x 10-4 | 0.031 | -0.038 | 0.015 | 0.013 | 17 | TSS1500 |  |
| *C11orf91* | cg01276497 | 52.62 ± 4.51 | 46.62 ± 5.51 | -6.0 | 2.9 x 10-4 | 0.048 | -0.026 | 0.012 | 0.043 | 11 | TSS1500 |  |
| *C1QTNF8* | cg09695996 | 33.44 ± 7.12 | 23.14 ± 5.03 | -10.3 | 1.6 x 10-5 | 0.015 | -0.047 | 0.020 | 0.020 | 16 | 3'UTR |  |
| *C1QTNF8* | cg09146900 | 23.43 ± 5.73 | 17.43 ± 2.99 | -6.0 | 2.5 x 10-5 | 0.018 | -0.053 | 0.019 | 0.006 | 16 | 3'UTR |  |
| *C8orf86* | cg23843812 | 27.19 ± 4.25 | 19.56 ± 4.32 | -7.6 | 6.5 x 10-6 | 0.011 | -0.032 | 0.015 | 0.033 | 8 | 1stExon |  |
| *CELSR1* | cg16845136 | 46.47 ± 6.40 | 39.30 ± 6.12 | -7.2 | 9.7 x 10-5 | 0.031 | -0.039 | 0.016 | 0.014 | 22 | Body |  |
| *CHAT* | cg13949086 | 39.61 ± 4.95 | 33.17 ± 4.51 | -6.4 | 2.0 x 10-4 | 0.041 | -0.023 | 0.011 | 0.048 | 10 | Body |  |
| *CHD3* | cg21068610 | 31.44 ± 4.83 | 23.03 ± 4.43 | -8.4 | 1.2 x 10-5 | 0.014 | -0.032 | 0.014 | 0.026 | 17 | Body |  |
| *CPA5* | cg20917077 | 64.47 ± 4.78 | 59.10 ± 4.36 | -5.4 | 5.0 x 10-5 | 0.024 | -0.033 | 0.016 | 0.038 | 7 | Body |  |
| *CPNE2* | cg02670686 | 30.13 ± 6.02 | 21.01 ± 5.61 | -9.1 | 8.4 x 10-5 | 0.029 | -0.041 | 0.018 | 0.019 | 16 | TSS1500 |  |
| *CYTSB* | cg20290367 | 74.60 ± 4.56 | 69.37 ± 4.34 | -5.2 | 6.7 x 10-5 | 0.027 | -0.034 | 0.017 | 0.050 | 17 | Body |  |
| *DENND3* | cg09946870 | 48.22 ± 6.60 | 40.49 ± 5.88 | -7.7 | 2.4 x 10-5 | 0.018 | -0.036 | 0.016 | 0.023 | 8 | Body |  |
| *DHDPSL* | cg27428414 | 40.72 ± 5.76 | 34.71 ± 4.56 | -6.0 | 2.3 x 10-4 | 0.044 | -0.037 | 0.015 | 0.015 | 10 | TSS200 |  |
| *DMD* | cg16116006 | 58.15 ± 11.72 | 48.32 ± 11.70 | -9.8 | 4.4 x 10-6 | 0.01 | -0.038 | 0.019 | 0.049 | X | Body |  |
| *DTNA* | cg22009464 | 46.94 ± 9.10 | 36.18 ± 8.20 | -10.8 | 1.2 x 10-4 | 0.033 | -0.053 | 0.025 | 0.030 | 18 | TSS200;5'UTR |  |
| *DTNA* | cg26530706 | 29.21 ± 5.52 | 22.37 ± 4.78 | -6.8 | 1.6 x 10-4 | 0.037 | -0.044 | 0.018 | 0.015 | 18 | TSS200;5'UTR |  |
| *DTX1* | cg05689110 | 51.58 ± 9.61 | 42.11 ± 8.05 | -9.5 | 2.8 x 10-4 | 0.047 | -0.051 | 0.026 | 0.047 | 12 | Body |  |
| *ENG* | cg13458609 | 47.20 ± 4.79 | 42.10 ± 4.89 | -5.1 | 1.6 x 10-4 | 0.037 | -0.033 | 0.014 | 0.018 | 9 | Body |  |
| *EPHA4* | cg08787401 | 32.21 ± 7.07 | 23.33 ± 4.74 | -8.9 | 5.6 x 10-5 | 0.025 | -0.047 | 0.022 | 0.031 | 2 | Body |  |
| *EPHA8* | cg11847808 | 29.79 ± 5.02 | 22.01 ± 3.58 | -7.8 | 3.8 x 10-5 | 0.021 | -0.033 | 0.015 | 0.028 | 1 | TSS1500 | Island |
| *EPS8* | cg08739221 | 43.02 ± 6.78 | 35.77 ± 5.28 | -7.3 | 2.9 x 10-4 | 0.048 | -0.036 | 0.017 | 0.037 | 12 | 5'UTR |  |
| *FAM134B* | cg15036894 | 45.22 ± 6.53 | 35.26 ± 7.56 | -10.0 | 3.0 x 10-6 | 0.009 | -0.035 | 0.017 | 0.039 | 5 | Body |  |
| *FLJ41941* | cg11961401 | 58.27 ± 8.39 | 51.39 ± 7.74 | -6.9 | 1.1 x 10-4 | 0.033 | -0.041 | 0.021 | 0.044 | 22 | TSS1500 | S Shelf |
| *FMO2* | cg18375642 | 68.35 ± 4.49 | 62.43 ± 5.34 | -5.9 | 1.5 x 10-4 | 0.036 | -0.027 | 0.013 | 0.040 | 1 | 5'UTR;1stExon |  |
| *GFI1B* | cg14457284 | 52.83 ± 5.86 | 46.95 ± 4.42 | -5.9 | 3.4 x 10-5 | 0.02 | -0.040 | 0.016 | 0.011 | 9 | TSS1500 |  |
| *GGT1* | cg25979829 | 68.01 ± 6.33 | 62.22 ± 6.64 | -5.8 | 2.0 x 10-4 | 0.041 | -0.042 | 0.019 | 0.028 | 22 | TSS1500;5'UTR |  |
| *GLS* | cg03661409 | 40.39 ± 6.93 | 32.07 ± 4.93 | -8.3 | 4.3 x 10-5 | 0.022 | -0.037 | 0.018 | 0.040 | 2 | Body |  |
| *HMGCS2* | cg10212621 | 30.33 ± 4.93 | 24.12 ± 4.14 | -6.2 | 2.9 x 10-5 | 0.019 | -0.030 | 0.013 | 0.027 | 1 | TSS200 |  |
| *HNF1B* | cg21250756 | 28.57 ± 5.12 | 20.71 ± 3.83 | -7.9 | 5.2 x 10-6 | 0.011 | -0.039 | 0.017 | 0.024 | 17 | Body |  |
| *HRNBP3* | cg08759569 | 41.09 ± 5.61 | 33.64 ± 4.78 | -7.5 | 8.5 x 10-6 | 0.012 | -0.038 | 0.014 | 0.007 | 17 | 5'UTR |  |
| *IGF2AS;INS-IGF2;IGF2* | cg11005826 | 40.17 ± 8.11 | 31.39 ± 4.82 | -8.8 | 1.7 x 10-4 | 0.038 | -0.039 | 0.020 | 0.047 | 11 | Body;5'UTR | S Shore |
| *IL1R2* | cg21674927 | 51.33 ± 4.01 | 45.76 ± 4.60 | -5.6 | 2.2 x 10-5 | 0.017 | -0.028 | 0.013 | 0.034 | 2 | Body |  |
| *INPP4B* | cg04084236 | 43.30 ± 5.48 | 36.15 ± 4.91 | -7.2 | 3.6 x 10-6 | 0.009 | -0.033 | 0.014 | 0.022 | 4 | 5'UTR | N Shore |
| *IQSEC1* | cg09437283 | 62.84 ± 5.76 | 55.86 ± 4.91 | -7.0 | 2.7 x 10-6 | 0.008 | -0.036 | 0.017 | 0.034 | 3 | Body | N Shore |
| *ITGA3* | cg13213536 | 58.55 ± 7.65 | 50.58 ± 7.65 | -8.0 | 2.0 x 10-4 | 0.041 | -0.046 | 0.021 | 0.025 | 17 | Body | S Shelf |
| *KRT222* | cg24222083 | 66.04 ± 6.16 | 59.20 ± 5.98 | -6.8 | 2.1 x 10-5 | 0.017 | -0.036 | 0.017 | 0.041 | 17 | TSS1500 |  |
| *LIMD1* | cg08062273 | 31.73 ± 4.63 | 24.66 ± 3.90 | -7.1 | 6.6 x 10-5 | 0.027 | -0.030 | 0.014 | 0.033 | 3 | Body |  |
| *LOC100270710* | cg12409149 | 43.36 ± 5.93 | 37.63 ± 4.59 | -5.7 | 1.5 x 10-4 | 0.036 | -0.040 | 0.020 | 0.038 | 10 | TSS1500 | S Shelf |
| *LOC145837* | cg12449813 | 33.56 ± 5.44 | 26.02 ± 4.27 | -7.5 | 2.0 x 10-5 | 0.017 | -0.035 | 0.017 | 0.039 | 15 | TSS1500 |  |
| *LOC285419* | cg01723706 | 41.02 ± 8.80 | 30.65 ± 8.09 | -10.4 | 1.0 x 10-4 | 0.031 | -0.047 | 0.023 | 0.043 | 4 | Body |  |
| *LOC728606* | cg02137495 | 46.11 ± 5.04 | 40.50 ± 5.73 | -5.6 | 1.8 x 10-4 | 0.039 | -0.027 | 0.013 | 0.034 | 18 | Body |  |
| *LPPR1* | cg14253327 | 35.18 ± 6.09 | 28.36 ± 5.83 | -6.8 | 2.8 x 10-4 | 0.048 | -0.035 | 0.018 | 0.048 | 9 | TSS1500 | N Shore |
| *LRRC20* | cg14373727 | 42.16 ± 6.28 | 34.39 ± 3.87 | -7.8 | 4.1 x 10-6 | 0.01 | -0.041 | 0.020 | 0.036 | 10 | Body |  |
| *MPRIP* | cg01838287 | 20.44 ± 3.32 | 15.13 ± 2.32 | -5.3 | 5.8 x 10-5 | 0.025 | -0.028 | 0.013 | 0.039 | 17 | Body | S Shelf |
| *MYO10* | cg15333932 | 48.90 ± 5.15 | 41.39 ± 5.15 | -7.5 | 3.8 x 10-5 | 0.021 | -0.030 | 0.014 | 0.040 | 5 | Body |  |
| *MYO1B* | cg03134230 | 52.87 ± 6.34 | 43.50 ± 5.31 | -9.4 | 3.2 x 10-4 | 0.05 | -0.035 | 0.016 | 0.027 | 2 | Body |  |
| *NAT8* | cg21376120 | 56.09 ± 3.92 | 50.81 ± 4.25 | -5.3 | 8.6 x 10-7 | 0.006 | -0.025 | 0.012 | 0.039 | 2 | 5'UTR;1stExon |  |
| *NECAB3;C20orf134* | cg12710480 | 38.54 ± 5.86 | 31.55 ± 7.29 | -7.0 | 3.2 x 10-4 | 0.05 | -0.042 | 0.017 | 0.014 | 20 | Body;TSS200 | N Shore |
| *NFIA* | cg00568550 | 47.38 ± 13.94 | 37.42 ± 11.72 | -10.0 | 8.2 x 10-5 | 0.029 | -0.091 | 0.031 | 0.004 | 1 | Body |  |
| *NFIA* | cg08037935 | 24.56 ± 6.06 | 17.43 ± 3.92 | -7.1 | 2.3 x 10-4 | 0.044 | -0.040 | 0.020 | 0.040 | 1 | Body;TSS1500;TSS200 | N Shore |
| *OPA3* | cg22706147 | 47.89 ± 7.40 | 39.94 ± 6.74 | -8.0 | 2.1 x 10-5 | 0.017 | -0.041 | 0.016 | 0.012 | 19 | 3'UTR | N Shore |
| *P2RX1* | cg07005444 | 34.57 ± 5.17 | 27.87 ± 4.50 | -6.7 | 1.6 x 10-5 | 0.015 | -0.028 | 0.012 | 0.025 | 17 | TSS1500 |  |
| *PBX1* | cg11155784 | 27.42 ± 6.16 | 19.62 ± 5.51 | -7.8 | 1.5 x 10-4 | 0.037 | -0.039 | 0.019 | 0.042 | 1 | Body |  |
| *PEBP4* | cg15854394 | 58.39 ± 5.46 | 52.22 ± 5.64 | -6.2 | 2.4 x 10-4 | 0.044 | -0.039 | 0.019 | 0.039 | 8 | Body |  |
| *PIGS* | cg14750778 | 52.85 ± 7.14 | 42.55 ± 6.17 | -10.3 | 4.7 x 10-5 | 0.023 | -0.035 | 0.018 | 0.047 | 17 | TSS1500 | S Shore |
| *POLR1A* | cg08814020 | 72.85 ± 8.40 | 63.97 ± 9.92 | -8.9 | 2.6 x 10-4 | 0.046 | -0.062 | 0.026 | 0.014 | 2 | Body |  |
| *PRDM16* | cg01261194 | 56.40 ± 6.96 | 50.10 ± 6.09 | -6.3 | 6.5 x 10-5 | 0.027 | -0.040 | 0.018 | 0.030 | 1 | Body | N Shore |
| *PTTG1IP* | cg22434923 | 53.34 ± 8.02 | 47.19 ± 5.09 | -6.2 | 1.0 x 10-4 | 0.031 | -0.044 | 0.020 | 0.025 | 21 | Body | N Shelf |
| *RARG* | cg13940444 | 63.99 ± 4.75 | 58.56 ± 5.45 | -5.4 | 8.3 x 10-5 | 0.029 | -0.032 | 0.015 | 0.031 | 12 | Body | S Shelf |
| *RNU5E;CKMT2* | cg23425533 | 41.60 ± 7.46 | 30.92 ± 5.99 | -10.7 | 1.1 x 10-4 | 0.032 | -0.044 | 0.018 | 0.017 | 5 | Body;5'UTR;1stExon |  |
| *SHQ1* | cg18720486 | 43.83 ± 7.11 | 34.42 ± 4.37 | -9.4 | 1.3 x 10-4 | 0.035 | -0.049 | 0.019 | 0.009 | 3 | Body |  |
| *SKI* | cg07114422 | 26.29 ± 5.05 | 19.36 ± 2.64 | -6.9 | 3.3 x 10-6 | 0.009 | -0.036 | 0.017 | 0.032 | 1 | Body |  |
| *SKI* | cg17165158 | 91.18 ± 5.32 | 85.93 ± 7.30 | -5.2 | 9.5 x 10-5 | 0.03 | -0.111 | 0.044 | 0.011 | 1 | Body |  |
| *SLC41A1* | cg10717869 | 23.12 ± 4.14 | 17.29 ± 3.13 | -5.8 | 1.2 x 10-4 | 0.033 | -0.059 | 0.017 | 0.001 | 1 | 5'UTR | N Shore |
| *SLC44A4* | cg18856043 | 38.16 ± 4.67 | 32.25 ± 2.97 | -5.9 | 3.6 x 10-5 | 0.021 | -0.023 | 0.011 | 0.049 | 6 | Body |  |
| *SLC5A10;FAM83G* | cg25708364 | 73.77 ± 5.63 | 68.61 ± 4.04 | -5.2 | 6.6 x 10-5 | 0.027 | -0.042 | 0.017 | 0.016 | 17 | Body | N Shore |
| *SPINK5L3* | cg01391299 | 46.26 ± 6.49 | 38.85 ± 6.49 | -7.4 | 2.9 x 10-4 | 0.048 | -0.031 | 0.016 | 0.049 | 5 | TSS200 |  |
| *SPP1* | cg15460348 | 63.29 ± 7.14 | 53.16 ± 7.13 | -10.1 | 3.2 x 10-5 | 0.02 | -0.044 | 0.022 | 0.043 | 4 | 1stExon;5'UTR |  |
| *SPP1* | cg20261167 | 68.19 ± 4.82 | 59.79 ± 6.95 | -8.4 | 2.9 x 10-5 | 0.019 | -0.033 | 0.017 | 0.046 | 4 | 1stExon;5'UTR |  |
| *STARD3* | cg20669414 | 44.97 ± 6.05 | 38.27 ± 5.34 | -6.7 | 2.4 x 10-4 | 0.045 | -0.040 | 0.015 | 0.007 | 17 | 5'UTR |  |
| *SYNPO* | cg13590277 | 40.29 ± 7.59 | 30.27 ± 7.25 | -10.0 | 1.2 x 10-4 | 0.033 | -0.038 | 0.018 | 0.032 | 5 | TSS1500;Body |  |
| *SYTL3* | cg19884556 | 21.37 ± 4.24 | 13.93 ± 3.30 | -7.5 | 3.6 x 10-6 | 0.009 | -0.042 | 0.020 | 0.032 | 6 | Body |  |
| *TEAD2* | cg01468567 | 33.85 ± 6.65 | 25.04 ± 4.83 | -8.8 | 4.6 x 10-5 | 0.023 | -0.043 | 0.018 | 0.018 | 19 | 3'UTR | S Shore |
| *TM4SF1* | cg16810293 | 33.63 ± 7.11 | 24.11 ± 5.06 | -9.5 | 2.5 x 10-5 | 0.018 | -0.048 | 0.020 | 0.016 | 3 | TSS200 |  |
| *TMEM51* | cg07180646 | 32.39 ± 5.50 | 23.46 ± 4.23 | -8.9 | 8.1 x 10-5 | 0.029 | -0.050 | 0.018 | 0.005 | 1 | 5'UTR | S Shore |
| *TNS1* | cg03323067 | 43.71 ± 8.86 | 33.55 ± 8.98 | -10.2 | 3.1 x 10-4 | 0.049 | -0.057 | 0.024 | 0.016 | 2 | 5'UTR |  |
| *TRIP6* | cg22851200 | 55.84 ± 4.99 | 50.44 ± 4.28 | -5.4 | 2.2 x 10-5 | 0.017 | -0.028 | 0.011 | 0.014 | 7 | Body | S Shore |
| *TRPM1* | cg21394729 | 56.22 ± 5.42 | 51.25 ± 6.65 | -5.0 | 2.1 x 10-5 | 0.017 | -0.031 | 0.015 | 0.040 | 15 | Body | N Shore |
| *VASH1* | cg23436960 | 40.28 ± 6.12 | 32.11 ± 5.30 | -8.2 | 2.5 x 10-4 | 0.045 | -0.035 | 0.015 | 0.017 | 14 | Body | N Shelf |
|  | cg00349404 | 40.60 ± 8.03 | 29.08 ± 7.98 | -11.5 | 2.6 x 10-4 | 0.046 | -0.051 | 0.021 | 0.015 | 10 |  | S Shore |
|  | cg01860706 | 53.71 ± 7.92 | 42.36 ± 8.28 | -11.4 | 1.5 x 10-4 | 0.037 | -0.051 | 0.022 | 0.022 | 15 |  |  |
|  | cg25862975 | 35.80 ± 7.21 | 24.45 ± 5.74 | -11.4 | 2.2 x 10-5 | 0.017 | -0.036 | 0.018 | 0.046 | 6 |  | N Shore |
|  | cg09703323 | 46.59 ± 8.41 | 35.68 ± 7.45 | -10.9 | 6.6 x 10-5 | 0.027 | -0.049 | 0.021 | 0.019 | 11 |  |  |
|  | cg09390594 | 41.51 ± 6.72 | 31.41 ± 6.99 | -10.1 | 1.4 x 10-4 | 0.035 | -0.034 | 0.017 | 0.039 | 14 |  |  |
|  | cg22670572 | 52.16 ± 8.49 | 42.46 ± 8.19 | -9.7 | 2.4 x 10-4 | 0.044 | -0.043 | 0.021 | 0.042 | 1 |  |  |
|  | cg09457766 | 34.18 ± 6.94 | 24.67 ± 5.72 | -9.5 | 4.2 x 10-5 | 0.022 | -0.046 | 0.020 | 0.022 | 22 |  | S Shelf |
|  | cg27139424 | 37.43 ± 7.38 | 27.94 ± 6.82 | -9.5 | 1.1 x 10-6 | 0.006 | -0.042 | 0.021 | 0.041 | 11 |  |  |
|  | cg14747813 | 41.94 ± 7.29 | 32.58 ± 6.81 | -9.4 | 2.2 x 10-4 | 0.043 | -0.040 | 0.019 | 0.036 | 8 |  |  |
|  | cg24741430 | 52.21 ± 7.53 | 42.87 ± 8.30 | -9.3 | 2.6 x 10-4 | 0.046 | -0.036 | 0.018 | 0.038 | 15 |  |  |
|  | cg26476925 | 41.13 ± 7.13 | 31.92 ± 7.12 | -9.2 | 1.2 x 10-4 | 0.033 | -0.036 | 0.016 | 0.024 | 19 |  |  |
|  | cg11688410 | 32.44 ± 5.92 | 23.46 ± 3.96 | -9.0 | 3.6 x 10-5 | 0.021 | -0.040 | 0.019 | 0.032 | 10 |  |  |
|  | cg11119313 | 37.42 ± 6.78 | 28.54 ± 5.37 | -8.9 | 3.1 x 10-4 | 0.049 | -0.045 | 0.021 | 0.027 | 17 |  | N Shelf |
|  | cg27305460 | 59.14 ± 6.17 | 50.27 ± 5.30 | -8.9 | 1.8 x 10-6 | 0.007 | -0.045 | 0.018 | 0.014 | 15 |  | S Shore |
|  | cg25587431 | 38.91 ± 6.71 | 30.17 ± 6.27 | -8.7 | 7.0 x 10-6 | 0.012 | -0.040 | 0.018 | 0.029 | 3 |  |  |
|  | cg13488013 | 38.26 ± 6.94 | 29.80 ± 6.65 | -8.5 | 1.6 x 10-5 | 0.015 | -0.039 | 0.018 | 0.031 | 17 |  | S Shelf |
|  | cg12254430 | 30.75 ± 6.01 | 22.52 ± 4.31 | -8.2 | 2.2 x 10-5 | 0.017 | -0.039 | 0.019 | 0.045 | 3 |  |  |
|  | cg10580269 | 26.45 ± 5.19 | 18.72 ± 4.79 | -7.7 | 3.4 x 10-5 | 0.02 | -0.040 | 0.017 | 0.018 | 2 |  |  |
|  | cg10809507 | 40.75 ± 6.08 | 33.11 ± 4.02 | -7.6 | 2.3 x 10-4 | 0.044 | -0.031 | 0.015 | 0.046 | 12 |  |  |
|  | cg17092519 | 29.84 ± 6.14 | 22.21 ± 4.96 | -7.6 | 3.1 x 10-4 | 0.05 | -0.034 | 0.017 | 0.042 | 11 |  | S Shore |
|  | cg15733007 | 69.83 ± 7.36 | 62.32 ± 7.43 | -7.5 | 3.2 x 10-4 | 0.05 | -0.046 | 0.020 | 0.025 | 6 |  |  |
|  | cg25004840 | 36.09 ± 6.48 | 28.69 ± 6.54 | -7.4 | 7.9 x 10-5 | 0.029 | -0.041 | 0.018 | 0.027 | 2 |  |  |
|  | cg20815992 | 38.68 ± 5.86 | 31.30 ± 4.40 | -7.4 | 3.6 x 10-5 | 0.021 | -0.033 | 0.016 | 0.048 | 11 |  | N Shelf |
|  | cg23080355 | 46.07 ± 6.79 | 38.69 ± 4.95 | -7.4 | 6.4 x 10-5 | 0.026 | -0.051 | 0.021 | 0.016 | 1 |  |  |
|  | cg27606671 | 48.27 ± 5.62 | 40.99 ± 3.07 | -7.3 | 6.0 x 10-5 | 0.026 | -0.031 | 0.013 | 0.019 | 11 |  |  |
|  | cg01501476 | 51.81 ± 6.95 | 44.55 ± 4.86 | -7.3 | 3.0 x 10-4 | 0.049 | -0.029 | 0.015 | 0.050 | 6 |  | Island |
|  | cg15808063 | 36.15 ± 5.19 | 29.03 ± 4.31 | -7.1 | 9.0 x 10-5 | 0.03 | -0.036 | 0.016 | 0.022 | 2 |  | N Shelf |
|  | cg25962657 | 42.12 ± 6.04 | 35.00 ± 4.65 | -7.1 | 7.9 x 10-6 | 0.012 | -0.031 | 0.015 | 0.046 | 7 |  | S Shelf |
|  | cg00078861 | 49.14 ± 6.02 | 42.14 ± 6.13 | -7.0 | 2.7 x 10-4 | 0.047 | -0.034 | 0.017 | 0.048 | 5 |  |  |
|  | cg05405914 | 59.44 ± 6.66 | 52.56 ± 6.67 | -6.9 | 2.1 x 10-4 | 0.042 | -0.034 | 0.017 | 0.046 | 16 |  | N Shore |
|  | cg16693313 | 32.02 ± 5.91 | 25.26 ± 6.23 | -6.8 | 1.2 x 10-4 | 0.034 | -0.040 | 0.017 | 0.023 | 10 |  |  |
|  | cg23322114 | 52.49 ± 5.67 | 45.76 ± 3.88 | -6.7 | 4.0 x 10-6 | 0.01 | -0.032 | 0.012 | 0.008 | 7 |  | S Shelf |
|  | cg21791252 | 35.16 ± 4.19 | 28.54 ± 4.33 | -6.6 | 6.0 x 10-5 | 0.026 | -0.026 | 0.012 | 0.044 | 6 |  |  |
|  | cg04976685 | 20.94 ± 5.93 | 14.34 ± 3.66 | -6.6 | 8.5 x 10-5 | 0.029 | -0.064 | 0.021 | 0.003 | 17 |  | Island |
|  | cg07820189 | 23.57 ± 4.14 | 16.98 ± 2.10 | -6.6 | 3.2 x 10-8 | 0.003 | -0.037 | 0.016 | 0.023 | 1 |  |  |
|  | cg11783364 | 31.38 ± 4.28 | 24.83 ± 4.29 | -6.6 | 3.9 x 10-5 | 0.022 | -0.025 | 0.012 | 0.041 | 12 |  |  |
|  | cg24547885 | 18.47 ± 4.94 | 12.03 ± 2.65 | -6.4 | 2.5 x 10-4 | 0.045 | -0.050 | 0.022 | 0.022 | 2 |  |  |
|  | cg13860928 | 67.27 ± 6.17 | 60.84 ± 6.70 | -6.4 | 1.4 x 10-4 | 0.035 | -0.036 | 0.018 | 0.044 | 1 |  |  |
|  | cg08530064 | 41.35 ± 5.57 | 34.93 ± 2.77 | -6.4 | 1.9 x 10-5 | 0.016 | -0.046 | 0.015 | 0.003 | 7 |  |  |
|  | cg07201717 | 44.39 ± 5.18 | 38.09 ± 3.83 | -6.3 | 3.9 x 10-5 | 0.021 | -0.030 | 0.015 | 0.043 | 1 |  |  |
|  | cg19750282 | 43.40 ± 5.37 | 37.10 ± 4.54 | -6.3 | 8.8 x 10-5 | 0.029 | -0.032 | 0.013 | 0.015 | 6 |  |  |
|  | cg26915889 | 57.05 ± 5.20 | 50.85 ± 5.63 | -6.2 | 6.9 x 10-6 | 0.012 | -0.034 | 0.015 | 0.025 | 1 |  |  |
|  | cg07380540 | 39.39 ± 5.22 | 33.35 ± 4.44 | -6.0 | 4.8 x 10-5 | 0.023 | -0.037 | 0.014 | 0.010 | 1 |  | S Shelf |
|  | cg20559943 | 39.29 ± 5.40 | 33.26 ± 4.08 | -6.0 | 2.4 x 10-4 | 0.045 | -0.028 | 0.013 | 0.031 | 4 |  |  |
|  | cg23705098 | 45.45 ± 5.57 | 39.43 ± 4.45 | -6.0 | 3.2 x 10-4 | 0.05 | -0.031 | 0.014 | 0.031 | 7 |  | S Shelf |
|  | cg00484122 | 27.59 ± 4.44 | 21.68 ± 3.16 | -5.9 | 5.8 x 10-5 | 0.025 | -0.035 | 0.016 | 0.028 | 2 |  |  |
|  | cg02398663 | 20.35 ± 4.25 | 14.68 ± 3.14 | -5.7 | 3.2 x 10-4 | 0.05 | -0.034 | 0.017 | 0.048 | 18 |  | Island |
|  | cg10024501 | 71.81 ± 5.45 | 66.16 ± 5.93 | -5.7 | 2.0 x 10-4 | 0.041 | -0.045 | 0.019 | 0.019 | 6 |  |  |
|  | cg16632159 | 52.12 ± 6.36 | 46.57 ± 5.57 | -5.6 | 3.1 x 10-4 | 0.049 | -0.034 | 0.014 | 0.021 | 10 |  |  |
|  | cg25766247 | 28.05 ± 3.89 | 22.51 ± 2.42 | -5.6 | 2.1 x 10-6 | 0.008 | -0.033 | 0.015 | 0.031 | 8 |  |  |
|  | cg11027717 | 18.42 ± 3.94 | 12.88 ± 2.43 | -5.5 | 1.2 x 10-5 | 0.014 | -0.036 | 0.018 | 0.047 | 11 |  |  |
|  | cg11245053 | 48.49 ± 5.33 | 42.99 ± 3.39 | -5.5 | 1.1 x 10-4 | 0.032 | -0.029 | 0.014 | 0.042 | 12 |  |  |
|  | cg25506501 | 73.47 ± 7.83 | 68.01 ± 6.37 | -5.5 | 3.8 x 10-5 | 0.021 | -0.041 | 0.020 | 0.043 | 3 |  |  |
|  | cg14844953 | 47.35 ± 5.44 | 42.06 ± 4.42 | -5.3 | 2.1 x 10-4 | 0.042 | -0.029 | 0.013 | 0.028 | 1 |  |  |
|  | cg22246636 | 45.96 ± 4.12 | 40.90 ± 3.54 | -5.1 | 1.0 x 10-4 | 0.031 | -0.021 | 0.009 | 0.035 | 20 |  | Island |
|  | cg09172850 | 61.42 ± 5.67 | 56.45 ± 7.90 | -5.0 | 1.8 x 10-4 | 0.039 | -0.037 | 0.016 | 0.017 | 1 |  | S Shelf |
