# Supplementary material for: Genome-Wide DNA Methylation Analysis of Human Pancreatic Islets from Type 2 Diabetic and Non-Diabetic Donors Identifies Candidate Genes That Influence Insulin Secretion
Source: PLoS Genet. 2014 Mar 6;10(3):e1004160. doi: 10.1371/journal.pgen.1004160 (PMC3945174; doi:10.1371/journal.pgen.1004160)
Supplement: Table S8 — CpG sites that exhibit differential DNA methylation (q<0.05 and difference in methylation ≥5%) in pancreatic islets from 34 non-diabetic versus 15 T2D human donors in parallel with an association between age and differential DNA methylation (P<0.05) in pancreatic islets from 87 non-diabetic donors. (DOCX) [file pgen.1004160.s013.docx]

**Table S8.** CpG sites that exhibit differential DNA methylation (*q* < 0.05 and difference in methylation ≥ 5%) in pancreatic islets from 34 non-diabetic versus 15 T2D human donors in parallel with an association between age and differential DNA methylation (*P* < 0.05) in pancreatic islets from 87 non-diabetic donors.

| **Gene symbol** | **Probe ID** | **Non-diabetic**  **DNA meth (%)**  **(mean±Sd)** | **T2D**  **DNA meth (%)**  **(mean±Sd)** | **Delta**  **DNA meth**  **(%)** | ***P*-value** | ***q*-value** | **Association between age**  **and DNA meth** | | | **Chr.** | **Gene region** | **Relation to CpG island** |
| --- | --- | --- | --- | --- | --- | --- | --- | --- | --- | --- | --- | --- |
|  |  |  |  |  |  |  | **beta-coef.** | **SEM** | ***P*-value** |  |  |  |
| *AGRN* | cg26222311 | 16.45 ± 5.51 | 21.48 ± 6.01 | 5.03 | 1.8 x 10-4 | 0.039 | 0.0170 | 0.0064 | 0.008 | 1 | Body | Island |
| *ANKRD56* | cg01751800 | 62.60 ± 3.82 | 56.21 ± 4.57 | -6.39 | 5.9 x 10-5 | 0.026 | -0.0060 | 0.0026 | 0.024 | 4 | TSS1500 | S Shore |
| *ANO8* | cg24192660 | 36.04 ± 6.85 | 44.98 ± 7.11 | 8.94 | 3.0 x 10-4 | 0.049 | 0.0122 | 0.0045 | 0.007 | 19 | Body | Island |
| *BANP* | cg07560587 | 76.18 ± 5.59 | 70.02 ± 6.02 | -6.16 | 1.8 x 10-4 | 0.039 | -0.0144 | 0.0051 | 0.005 | 16 | Body | N Shelf |
| *C20orf54* | cg11959399 | 23.36 ± 6.62 | 31.65 ± 6.79 | 8.29 | 7.1 x 10-5 | 0.027 | 0.0115 | 0.0057 | 0.038 | 20 | Body | Island |
| *C22orf45;UPB1* | cg16592832 | 55.23 ± 4.73 | 49.80 ± 6.58 | -5.42 | 1.7 x 10-4 | 0.038 | 0.0066 | 0.0031 | 0.036 | 22 | TSS1500;TSS200 | Island |
| *CDKN1A* | cg05460965 | 55.69 ± 3.82 | 46.79 ± 3.71 | -8.9 | 3.2 x 10-7 | 0.004 | -0.0075 | 0.0027 | 0.006 | 6 | TSS1500 | N Shore |
| *CHI3L2* | cg26366091 | 63.31 ± 4.62 | 58.01 ± 5.73 | -5.3 | 7.5 x 10-6 | 0.012 | -0.0061 | 0.0029 | 0.038 | 1 | TSS200 |  |
| *CMAH* | cg12992112 | 50.90 ± 7.53 | 39.57 ± 9.18 | -11.33 | 2.4 x 10-4 | 0.044 | -0.0080 | 0.0039 | 0.038 | 6 | TSS200;TSS1500 | N Shore |
| *EHF* | cg15994604 | 53.95 ± 4.42 | 47.73 ± 5.14 | -6.23 | 2.3 x 10-4 | 0.044 | -0.0082 | 0.0033 | 0.013 | 11 | Body |  |
| *EPHA8* | cg01628425 | 33.89 ± 4.41 | 28.93 ± 4.07 | -4.96 | 2.5 x 10-6 | 0.008 | -0.0076 | 0.0035 | 0.030 | 1 | Body | N Shore |
| *EPS8L1* | cg21121496 | 14.72 ± 5.02 | 21.98 ± 6.95 | 7.27 | 8.6 x 10-5 | 0.029 | 0.0139 | 0.0061 | 0.022 | 19 | Body | Island |
| *EXOC3L2* | cg26787220 | 34.75 ± 5.86 | 40.20 ± 6.81 | 5.44 | 3.1 x 10-4 | 0.049 | 0.0086 | 0.0039 | 0.026 | 19 | Body | Island |
| *IL1R2* | cg21674927 | 51.33 ± 4.01 | 45.76 ± 4.60 | -5.57 | 2.2 x 10-5 | 0.017 | -0.0079 | 0.0025 | 0.002 | 2 | Body |  |
| *ITGB4* | cg16916914 | 24.96 ± 6.45 | 30.29 ± 8.02 | 5.33 | 2.6 x 10-4 | 0.046 | 0.0114 | 0.0056 | 0.038 | 17 | Body | Island |
| *KCNIP2* | cg20564892 | 19.72 ± 5.77 | 28.85 ± 7.38 | 9.14 | 3.2 x 10-5 | 0.02 | 0.0179 | 0.0060 | 0.003 | 10 | 1stExon;5'UTR | Island |
| *LOC100128239* | cg06769296 | 34.64 ± 5.97 | 27.08 ± 5.95 | -7.56 | 1.1 x 10-4 | 0.032 | 0.0065 | 0.0031 | 0.039 | 11 | Body | Island |
| *LOC389333* | cg09156097 | 30.72 ± 6.34 | 36.64 ± 7.85 | 5.91 | 1.3 x 10-4 | 0.034 | 0.0121 | 0.0044 | 0.006 | 5 | 1stExon | Island |
| *MAGIX* | cg06663923 | 59.91 ± 7.14 | 53.53 ± 5.03 | -6.38 | 1.6 x 10-4 | 0.037 | -0.0045 | 0.0022 | 0.046 | X | Body;1stExon;5'UTR | N Shore |
| *MICAL2* | cg16946439 | 27.59 ± 3.84 | 22.29 ± 3.04 | -5.29 | 1.9 x 10-5 | 0.016 | 0.0062 | 0.0023 | 0.008 | 11 | TSS1500 | Island |
| *NBEAL2* | cg13451127 | 33.25 ± 6.65 | 39.07 ± 5.33 | 5.82 | 2.9 x 10-4 | 0.048 | 0.0087 | 0.0042 | 0.039 | 3 | Body | Island |
| *PDGFB* | cg11714334 | 14.78 ± 4.99 | 8.52 ± 2.52 | -6.26 | 1.3 x 10-4 | 0.034 | 0.0097 | 0.0045 | 0.028 | 22 | Body;TSS1500 | N Shore |
| *PLA2G6* | cg17652424 | 64.68 ± 6.10 | 58.15 ± 6.91 | -6.53 | 1.2 x 10-5 | 0.014 | -0.0090 | 0.0038 | 0.017 | 22 | 5'UTR | N Shelf |
| *PRICKLE2* | cg22083047 | 71.82 ± 3.79 | 65.21 ± 3.27 | -6.61 | 2.2 x 10-7 | 0.004 | -0.0068 | 0.0027 | 0.013 | 3 | TSS1500 |  |
| *ROR1* | cg13300480 | 56.38 ± 6.09 | 46.40 ± 5.76 | -9.98 | 3.1 x 10-7 | 0.004 | -0.0070 | 0.0032 | 0.027 | 1 | Body |  |
| *SLC41A1* | cg10717869 | 23.12 ± 4.14 | 17.29 ± 3.13 | -5.83 | 1.2 x 10-4 | 0.033 | -0.0093 | 0.0033 | 0.005 | 1 | 5'UTR | N Shore |
| *SPATA18* | cg24395452 | 31.54 ± 5.50 | 38.61 ± 5.14 | 7.06 | 1.8 x 10-4 | 0.039 | 0.0135 | 0.0042 | 0.001 | 4 | Body | Island |
| *SPP1* | cg15460348 | 63.29 ± 7.14 | 53.16 ± 7.13 | -10.13 | 3.2 x 10-5 | 0.02 | -0.0087 | 0.0042 | 0.039 | 4 | 1stExon;5'UTR |  |
| *TULP4* | cg07159114 | 69.57 ± 7.18 | 62.00 ± 5.88 | -7.57 | 1.0 x 10-4 | 0.031 | 0.0116 | 0.0046 | 0.012 | 6 | Body |  |
|  | cg16113254 | 33.75 ± 5.04 | 25.07 ± 3.00 | -8.68 | 1.8 x 10-5 | 0.016 | -0.0058 | 0.0029 | 0.044 | 14 |  |  |
|  | cg24766327 | 55.09 ± 5.20 | 48.76 ± 3.88 | -6.34 | 1.9 x 10-5 | 0.016 | -0.0071 | 0.0030 | 0.019 | 10 |  |  |
|  | cg02560388 | 34.21 ± 4.53 | 27.88 ± 4.65 | -6.33 | 6.5 x 10-5 | 0.027 | -0.0054 | 0.0027 | 0.049 | 2 |  |  |
|  | cg00164941 | 37.56 ± 4.13 | 31.95 ± 4.77 | -5.61 | 2.1 x 10-4 | 0.042 | -0.0058 | 0.0029 | 0.044 | 2 |  | S Shelf |
|  | cg27085488 | 59.95 ± 5.07 | 54.41 ± 6.51 | -5.55 | 1.5 x 10-4 | 0.036 | 0.0068 | 0.0027 | 0.013 | 5 |  |  |
|  | cg11239720 | 35.78 ± 9.57 | 30.50 ± 5.40 | -5.28 | 7.1 x 10-6 | 0.012 | 0.0083 | 0.0039 | 0.033 | 4 |  |  |
