# Supplementary material for: Genome-Wide DNA Methylation Analysis of Human Pancreatic Islets from Type 2 Diabetic and Non-Diabetic Donors Identifies Candidate Genes That Influence Insulin Secretion
Source: PLoS Genet. 2014 Mar 6;10(3):e1004160. doi: 10.1371/journal.pgen.1004160 (PMC3945174; doi:10.1371/journal.pgen.1004160)
Supplement: Table S9 — CpG sites that exhibit differential DNA methylation (q<0.05 and difference in methylation ≥5%) in pancreatic islets from 34 non-diabetic versus 15 T2D human donors in parallel with an association between BMI and differential DNA methylation (P<0.05) in pancreatic islets from 87 non-diabetic donors. (DOCX) [file pgen.1004160.s014.docx]

**Table S9.** CpG sites that exhibit differential DNA methylation (*q* < 0.05 and difference in methylation ≥ 5%) in pancreatic islets from 34 non-diabetic versus 15 T2D human donors in parallel with an association between BMI and differential DNA methylation (*P* < 0.05) in pancreatic islets from 87 non-diabetic donors.

| **Gene symbol** | **Probe ID** | **Non-diabetic**  **DNA meth (%)**  **(mean ± Sd)** | **T2D**  **DNA meth (%)**  **(mean ± Sd)** | **Delta**  **DNA meth (%)** | ***P*-value** | ***q*-value** | **Association between BMI**  **and DNA meth** | | | **Chr.** | **Gene region** | **Relation to**  **CpG island** |
| --- | --- | --- | --- | --- | --- | --- | --- | --- | --- | --- | --- | --- |
|  |  |  |  |  |  |  | **Beta Coef.** | **SEM** | ***P*-value** |  |  |  |
| *ACMSD* | cg02499308 | 76.29 ± 5.35 | 69.94 ± 5.99 | -6.4 | 1.7 x 10-5 | 0.016 | -0.042 | 0.012 | 0.001 | 2 | TSS1500 |  |
| *ATP11A* | cg04229372 | 65.82 ± 7.59 | 78.57 ± 5.36 | 12.8 | 2.3 x 10-5 | 0.017 | 0.033 | 0.015 | 0.022 | 13 | Body | S Shelf |
| *BMP4* | cg16389901 | 81.87 ± 5.09 | 75.83 ± 6.98 | -6.0 | 7.6 x 10-5 | 0.028 | -0.034 | 0.016 | 0.032 | 14 | TSS1500 | S Shore |
| *C6orf208;C6orf122* | cg15855251 | 52.10 ± 5.27 | 45.95 ± 4.39 | -6.2 | 6.5 x 10-6 | 0.011 | 0.019 | 0.009 | 0.036 | 6 | Body;TSS1500 |  |
| *C9orf3* | cg14276379 | 51.75 ± 6.03 | 44.78 ± 5.79 | -7.0 | 8.0 x 10-5 | 0.029 | -0.023 | 0.011 | 0.026 | 9 | Body |  |
| *CORO1C* | cg09182455 | 74.64 ± 4.39 | 69.69 ± 4.76 | -5.0 | 2.8 x 10-5 | 0.019 | -0.020 | 0.009 | 0.025 | 12 | 5'UTR |  |
| *CYR61* | cg22123915 | 78.86 ± 4.25 | 73.59 ± 6.45 | -5.3 | 2.6 x 10-7 | 0.004 | -0.023 | 0.011 | 0.032 | 1 | Body | S Shore |
| *GNG8* | cg19242688 | 44.28 ± 6.87 | 39.09 ± 6.80 | -5.2 | 9.4 x 10-5 | 0.03 | -0.025 | 0.012 | 0.035 | 19 | TSS1500 | Island |
| *LOC100130872-SPON2* | cg12407867 | 30.85 ± 7.61 | 38.57 ± 7.14 | 7.7 | 2.4 x 10-4 | 0.044 | 0.027 | 0.014 | 0.049 | 4 | Body;TSS1500 | S Shore |
| *PCSK6* | cg07983363 | 58.41 ± 7.07 | 49.46 ± 6.55 | -9.0 | 2.4 x 10-5 | 0.018 | -0.026 | 0.013 | 0.038 | 15 | Body |  |
| *SKI* | cg21775570 | 25.04 ± 4.60 | 19.36 ± 2.66 | -5.7 | 5.4 x 10-5 | 0.025 | 0.023 | 0.011 | 0.030 | 1 | Body |  |
| *SLC17A2* | cg20373747 | 56.60 ± 4.90 | 51.35 ± 4.43 | -5.3 | 2.2 x 10-5 | 0.017 | 0.020 | 0.008 | 0.015 | 6 | TSS200 |  |
| *SLC9A3R2* | cg08074555 | 71.55 ± 4.90 | 65.75 ± 5.81 | -5.8 | 2.4 x 10-4 | 0.045 | -0.025 | 0.011 | 0.030 | 16 | Body | N Shore |
| *STARD3* | cg20669414 | 44.97 ± 6.05 | 38.27 ± 5.34 | -6.7 | 2.4 x 10-4 | 0.045 | 0.018 | 0.009 | 0.039 | 17 | 5'UTR |  |
| *STK32B* | cg25612057 | 38.69 ± 4.14 | 33.35 ± 4.53 | -5.3 | 1.3 x 10-4 | 0.034 | 0.021 | 0.009 | 0.028 | 4 | Body |  |
| *TMEM8B* | cg14148156 | 72.38 ± 3.88 | 67.20 ± 4.33 | -5.2 | 5.8 x 10-6 | 0.011 | 0.019 | 0.008 | 0.019 | 9 | Body | N Shore |
|  | cg02788637 | 36.14 ± 7.26 | 27.75 ± 8.30 | -8.4 | 2.9 x 10-4 | 0.048 | -0.025 | 0.012 | 0.042 | 7 |  | Island |
|  | cg23552820 | 65.09 ± 8.52 | 58.04 ± 13.58 | -7.0 | 1.7 x 10-4 | 0.038 | -0.045 | 0.017 | 0.008 | 17 |  |  |
|  | cg24024528 | 67.15 ± 4.49 | 60.46 ± 5.08 | -6.7 | 7.3 x 10-5 | 0.028 | -0.020 | 0.009 | 0.038 | 12 |  |  |
|  | cg16932827 | 72.03 ± 6.24 | 66.00 ± 6.64 | -6.0 | 4.4 x 10-5 | 0.023 | -0.026 | 0.012 | 0.029 | 3 |  | S Shore |
|  | cg23683800 | 45.76 ± 4.12 | 39.77 ± 5.06 | -6.0 | 3.1 x 10-5 | 0.02 | -0.017 | 0.009 | 0.048 | 10 |  |  |
|  | cg22977481 | 51.70 ± 4.15 | 46.06 ± 3.93 | -5.6 | 2.3 x 10-7 | 0.004 | 0.015 | 0.007 | 0.035 | 2 |  |  |
