# Supplementary material for: Genome-Wide DNA Methylation Analysis of Human Pancreatic Islets from Type 2 Diabetic and Non-Diabetic Donors Identifies Candidate Genes That Influence Insulin Secretion
Source: PLoS Genet. 2014 Mar 6;10(3):e1004160. doi: 10.1371/journal.pgen.1004160 (PMC3945174; doi:10.1371/journal.pgen.1004160)
Supplement: Table S10 — Associations between DNA methylation and gene expression in pancreatic islets from 87 non-diabetic donors of CpG sites also showing differential DNA methylation (q<0.05 and difference in methylation ≥5%) concurrent with a difference in mRNA expression (P≤0.05) of the nearest gene in pancreatic islets from 34 non-diabetic versus 15 T2D human donors. (DOCX) [file pgen.1004160.s015.docx]

**Table S10.** Associations between DNA methylation and gene expression in pancreatic islets from 87 non-diabetic donors of CpG sites also showing differential DNA methylation (*q* < 0.05 and difference in methylation ≥5%) concurrent with a difference in mRNA expression (*P* ≤ 0.05) of the nearest gene in pancreatic islets from 34 non-diabetic versus 15 T2D human donors.

| **Probe ID** | **Gene symbol** | **Regression coeff** | **Regression coeff** | ***P*-value** |
| --- | --- | --- | --- | --- |
| **DNA meth** |  |  | **sem** | **Assoc meth - expr** |
| **cg21667069** | ***ALDH3B1*** | **-0.11** | **0.052** | **4.4 x 10-5** |
| **cg18254586** | ***ARHGAP26*** | **-0.17** | **0.078** | **4.1 x 10-6** |
| **cg19795338** | ***ATP13A4*** | **-0.21** | **0.099** | **3.4 x 10-8** |
| **cg23169762** | ***C6orf145*** | **-0.20** | **0.097** | **2.6 x 10-6** |
| **cg24425727** | ***CDKN1A*** | **-0.18** | **0.089** | **0.039** |
| **cg15474579** | ***CDKN1A*** | **-0.17** | **0.075** | **0.020** |
| **cg03714916** | ***CDKN1A*** | **-0.39** | **0.123** | **0.022** |
| **cg05460965** | ***CDKN1A*** | **-0.16** | **0.056** | **0.0036** |
| **cg21091547** | ***CDKN1A*** | **-0.37** | **0.173** | **0.018** |
| **cg11215644** | ***EDN1*** | **-0.22** | **0.087** | **2.0 x 10-4** |
| **cg15994604** | ***EHF*** | **-0.17** | **0.075** | **2.6 x 10-4** |
| **cg08739221** | ***EPS8*** | **-0.05** | **0.019** | **0.0057** |
| **cg26807095** | ***GMDS*** | **-0.24** | **0.119** | **4.9 x 10-5** |
| **cg10583414** | ***HDAC7*** | **-0.37** | **0.148** | **0.0019** |
| **cg08817540** | ***HHLA2*** | **-0.61** | **0.234** | **8.1 x 10-7** |
| **cg21674927** | ***IL1R2*** | **-0.30** | **0.142** | **0.049** |
| **cg22797169** | ***IL1RL2*** | **-0.06** | **0.031** | **0.014** |
| **cg04751089** | ***IRS1*** | **-0.26** | **0.087** | **8.5 x 10-4** |
| **cg13213536** | ***ITGA3*** | **-0.14** | **0.058** | **1.0 x 10-5** |
| **cg11383291** | ***ITGB5*** | **-0.18** | **0.071** | **5.1 x 10-4** |
| **cg10369242** | ***KRT7*** | **-0.28** | **0.110** | **2.1 x 10-7** |
| **cg07925587** | ***KRT80*** | **-0.25** | **0.093** | **8.3 x 10-4** |
| **cg21157065** | ***LCN2*** | **-0.35** | **0.163** | **2.6 x 10-4** |
| **cg24718756** | ***MARCH3*** | **-0.55** | **0.267** | **0.011** |
| **cg11839681** | ***MST1R*** | **-0.24** | **0.099** | **0.0027** |
| **cg13772742** | ***NR2F2*** | **-0.04** | **0.018** | **1.3 x 10-4** |
| **cg23716141** | ***SEPT9*** | **-0.17** | **0.084** | **0.0010** |
| **cg19654743** | ***SEPT9*** | **-0.16** | **0.075** | **0.0013** |
| **cg20772590** | ***SEPT9*** | **-0.19** | **0.090** | **0.0012** |
| **cg21204860** | ***SEPT9*** | **-0.26** | **0.121** | **0.0088** |
| **cg01642901** | ***SH3RF1*** | **-0.56** | **0.149** | **3.2 x 10-5** |
| **cg16793173** | ***SH3RF1*** | **-0.38** | **0.169** | **0.045** |
| **cg03359362** | ***SLC1A5*** | **-0.54** | **0.168** | **9.7 x 10-4** |
| **cg20627046** | ***SPATS2L*** | **-0.26** | **0.122** | **1.5 x 10-7** |
| **cg14361895** | ***SPATS2L*** | **-0.21** | **0.090** | **1.3 x 10-4** |
| **cg27121538** | ***SV2B*** | **-0.16** | **0.082** | **0.015** |
| **cg19247032** | ***TFF1*** | **-0.16** | **0.074** | **2.1 x 10-4** |
| **cg21167963** | ***TJP2*** | **-0.40** | **0.185** | **5.3 x 10-5** |
| **cg23246821** | ***TM4SF1*** | **-0.27** | **0.129** | **5.4 x 10-4** |
| **cg01214847** | ***TMPRSS3*** | **-0.44** | **0.110** | **1.7 x 10-9** |
| **cg07374224** | ***TMPRSS3*** | **-0.69** | **0.254** | **0.033** |
| **cg21239001** | ***TNFAIP8*** | **-0.70** | **0.328** | **4.3 x 10-5** |
| **cg06407111** | ***TRIM47*** | **-0.52** | **0.185** | **7.1 x 10-5** |
| cg19772011 | *GLP1R* | 0.25 | 0.116 | 0.0081 |
| cg07843390 | *GNG7* | 0.27 | 0.102 | 7.2 x 10-4 |
| cg09913813 | *KIRREL3* | 0.25 | 0.098 | 0.0025 |
| cg10485752 | *MAN1C1* | 0.17 | 0.069 | 0.0035 |
| cg21376120 | *NAT8* | 0.17 | 0.081 | 0.0082 |
| cg17081914 | *NCKAP5* | 0.12 | 0.051 | 0.0090 |
| cg01577114 | *PRKCH* | 0.41 | 0.110 | 6.6 x 10-4 |
| cg19859698 | *PRKCH* | 0.23 | 0.107 | 2.1 x 10-5 |
| cg02711647 | *SIX2* | 0.43 | 0.128 | 0.033 |
| cg23721586 | *TGFBR3* | 0.23 | 0.113 | 3.7 x 10-6 |
| cg21643178 | *TRIM15* | 0.29 | 0.137 | 4.4 x 10-6 |
| cg13567542 | *TRIM15* | 0.40 | 0.170 | 4.0 x 10-6 |

Probes shown in bold are CpG sites with negative association between DNA methylation and gene expression .
