# Supplementary material for: Genome-Wide DNA Methylation Analysis of Human Pancreatic Islets from Type 2 Diabetic and Non-Diabetic Donors Identifies Candidate Genes That Influence Insulin Secretion
Source: PLoS Genet. 2014 Mar 6;10(3):e1004160. doi: 10.1371/journal.pgen.1004160 (PMC3945174; doi:10.1371/journal.pgen.1004160)
Supplement: Table S12 — Sequences of CDKN1A and PDE7B inserted into the CpG-free firefly luciferase reporter vector (pCpGL-basic) and used for luciferase experiments. (DOCX) [file pgen.1004160.s017.docx]

**Table S12.** Sequences of *CDKN1A* and *PDE7B* inserted into the CpG-free firefly luciferase reporter vector (pCpGL-basic) and used for luciferase experiments.

| **Gene** | **Sequence** |
| --- | --- |
| *CDKN1A* | 5’-TGCTACTGTGTCCTCCCACCCCTACCTGGGCTCCCATCCCCACAGCAGAGGAGAAAGAAG |
| 1500bp | CCTGTCCTCCCCGAGGTCAGCTGCGTTAGAGGAAGAAGACTGGGCATGTCTGGGCAGAGA |
|  | TTTCCAGACTCTGAGCAGCCTGAGATGTCAGTAATTGTAGCTGCTCCAAGCCTGGGTTCT |
|  | GTTTTTTAGTGGGATTTCTGTTCAGATGAACAATCCATCCTCTGCAATTTTTTAAAAGCA |
|  | AAACTGCAAATGTTTCAGGCACAGAAAGGAGGCAAAGGTGAAGTCCAGGGGAGGTCAGGG |
|  | GTGTGAGGTAGATGGGAGCGGATAGACACATCACTCATTTCTGTGTCTGTCAGAAGAACC |
|  | AGTAGACACTTCCAGAATTGTCCTTTATTTATGTCATCTCCATAAACCATCTGCAAATGA |
|  | GGGTTATTTGGCATTTTTGTCATTTTGGAGCCACAGAAATAAAGGATGACAAGCAGAGAG |
|  | CCCCGGGCAGGAGGCAAAAGTCCTGTGTTCCAACTATAGTCATTTCTTTGCTGCATGATC |
|  | TGAGTTAGGTCACCAGACTTCTCTGAGCCCCAGTTTCCCCAGCAGTGTATACGGGCTATG |
|  | TGGGGAGTATTCAGGAGACAGACAACTCACTCGTCAAATCCTCCCCTTCCTGGCCAACAA |
|  | AGCTGCTGCAACCACAGGGATTTCTTCTGTTCAGGTGAGTGTAGGGTGTAGGGAGATTGG |
|  | TTCAATGTCCAATTCTTCTGTTTCCCTGGAGATCAGGTTGCCCTTTTTTGGTAGTCTCTC |
|  | CAATTCCCTCCTTCCCGGAAGCATGTGACAATCAACAACTTTGTATACTTAAGTTCAGTG |
|  | GACCTCAATTTCCTCATCTGTGAAATAAACGGGACTGAAAAATCATTCTGGCCTCAAGAT |
|  | GCTTTGTTGGGGTGTCTAGGTGCTCCAGGTGCTTCTGGGAGAGGTGACCTAGTGAGGGAT |
|  | CAGTGGGAATAGAGGTGATATTGTGGGGCTTTTCTGGAAATTGCAGAGAGGTGCATCGTT |
|  | TTTATAATTTATGAATTTTTATGTATTAATGTCATCCTCCTGATCTTTTCAGCTGCATTG |
|  | GGTAAATCCTTGCCTGCCAGAGTGGGTCAGCGGTGAGCCAGAAAGGGGGCTCATTCTAAC |
|  | AGTGCTGTGTCCTCCTGGAGAGTGCCAACTCATTCTCCAAGTAAAAAAAGCCAGATTTGT |
|  | GGCTCACTTCGTGGGGAAATGTGTCCAGCGCACCAACGCAGGCGAGGGACTGGGGGAGGA |
|  | GGGAAGTGCCCTCCTGCAGCACGCGAGGTTCCGGGACCGGCTGGCCTGCTGGAACTCGGC |
|  | CAGGCTCAGCTGGCTCGGCGCTGGGCAGCCAGGAGCCTGGGCCCCGGGGAGGGCGGTCCC |
|  | GGGCGGCGCGGTGGGCCGAGCGCGGGTCCCGCCTCCTTGAGGCGGGCCCGGGCGGGGCGG |
|  | TTGTATATCAGGGCCGCGCTGAGCTGCGCCAGCTGAGGTGTGAGCAGCTGCCGAAGTCAG-3’ |
| *PDE7B* | 5’-AGATAAACCCCAACAACTACAGGGATGCTTAATCCTAGCGCCTAGGCTCTTAGGGATACA |
| 1500bp | TAAATACTTTCTTTCTCAAAGAAACAATATTAGTATTTGAAAAAGGAATGTGGACATTCT |
|  | TTAGTGTTGCGCCCACCCCTAAAACATAGTCTGCCTCGGAGCAATGGAGAAATCTGATTG |
|  | GCCCCTGTGGGTCCCATGCCCCCTTGTGGCTCTGTCGTGGGAATGGGGCTGGGGGCAAAG |
|  | TCCATGCAATCCATAGAAGATAGAGGAAGGAAAGTTCCGCCCACCGCCCACCCAAAAAAG |
|  | GTCTGTTAGCAGAATTTAGGATGGGGATGGGGAAAAGGAGGAGGATGTGGGGGAGGCAGA |
|  | AACAACAGATGGCAACTATGGGGATTTTTCCAGAGTGGGAAGTGTAACCCAGGGATAAGC |
|  | TGGGGTCCCTGGTAAAGGAAGTATCCCTAAATAACTGGACCTTCATACTCTTTGGGAAAG |
|  | AACAAGCTGAGGCCCCAGGCACACCTGCATGGCATCACCATAGCCTTGCTTCTTGAATGA |
|  | CTCCTGCTGGCATCAGGAGGCATCTCCCTGGCAGGAACAAGCCTGAGGCTTCACCACGGA |
|  | AGCTGGAGGAAACGCAGGGACCCATGGCACAAACTGACACAGGAGTTCTATGTTTCATTG |
|  | CACATCATTTTGTTCACCAGTGCATTTGTACGATTCTGCCTCTATGGCTATCAAGTTTAA |
|  | TTAACAACTCATTTTTAGGTAAGAGTAAAAATGAATTAGGTAAAAAAAAATAAGCAACAG |
|  | CTGCCAAGTGAGCATGGAGCACATACACTCACAGATGGAAAAAATCTACATCTTGCTGCT |
|  | ATGATTTAGCTCAGGGTTTTATATTTAATTTTTGTTTTGTTTTGCTTTTGTTTTTATACA |
|  | CCTCACCACAAGGTATTTTAAATTCTTTAAATGTATTCCCGAATATAGAAAATGTTTCTT |
|  | GGGCTCTCCTGAGAGTGGGGTGCCCAGGCTGACCACAGGCACACACACATTCTTCCCATA |
|  | GTACAGTACTTTAAAATTAATAGCACATGCAGAGGAAAAACTAAACCCTTTGACGTCACT |
|  | CTTGAAATGAGGAAACATAAACAGAAAAGGCTCCACTCCAGTAAGCTGCCAGAGTTGAAG |
|  | CCGATTGGTCTCTGCTTTTCCTTGCCTGTGGCAACTGACAGCACCTCCCAGCCTAGCAGG |
|  | CCGGCGGCTGCTCACCCAGCCAGTCAGTTGGTCTGGGCACTGCAGCAGGCTCGGCTCTGT |
|  | CCCAGCACTTGTCTGGGAGAAAAGTGGTGTTACTCACCCAGGGAGAGTCTCTCTTTCTAC |
|  | CTTCCTTCTTTCTCGATCTCCTTGTGTGCTTTTGTGTTTCTTTATTTCTTTTCCTTTTTT |
|  | TTCTTTTTTTTTTTTTGTTACTTAATTATATTCCTAATCCTGGATGAAGTTGCTGGATTC |
|  | TGCAGCACAAGTCTTCATGAACAAGCAGCACCGCTCAGAGATTTCACGGCATTCAAAGGT-3’ |
