# Supplementary material for: Genome-Wide DNA Methylation Analysis of Human Pancreatic Islets from Type 2 Diabetic and Non-Diabetic Donors Identifies Candidate Genes That Influence Insulin Secretion
Source: PLoS Genet. 2014 Mar 6;10(3):e1004160. doi: 10.1371/journal.pgen.1004160 (PMC3945174; doi:10.1371/journal.pgen.1004160)
Supplement: Table S13 — Sequences of Cdkn1a, Pde7b and Sept9 inserted into pcDNA3.1 expression vectors and used for the overexpression experiments. (DOCX) [file pgen.1004160.s018.docx]

**Table S13.** Sequences of *Cdkn1a*, *Pde7b* and *Sept9* inserted into pcDNA3.1 expression vectors and used for the overexpression experiments.

| Gene | DNA sequence |
| --- | --- |
| *Cdkn1a* (507 bp) | 5’-GCTAGCATGTCCGATCCTGGTGATGTCCGACCTGTTCCACACAGGAGCAAAGTATGCCGTCGTCTGTTCGGTCCCGTGGACAGTGAGCAGTTGAGCCGCGATTGCGATGCGCTCATGGCGAGCTGTCTCCAGGAGGCCCGAGAACGGTGGAACTTTGACTTCGCCACTGAGACGCCACTGGAGGGCAACTACGTCTGGGAGCGTGTTCGGAGCCCAGGGCTGCCCAAGATCTACCTGAGCCCTGGGTCCCGCCGCCGTGATGACCTGGGAGGGGACAAGAGGCCCAGTACCTCCTCGGCCCTGCTGCAGGGGCCAGGGCCAGCTCCGGAGGACCACGTGGCCTTGTCGCTGTCTTGCACTCTGGTGTCTCACGCCCCTGAGAGGCCTGAAGACTCCCCGGGCGGGACCGGGACATCTCAGGGCCGAAAACGGAGGCAGACCAGCCTAACAGATTTCTATCACTCCAAGCGCCGATTGGTCTTCTGCAAGA GAAAGCCCTGACTCGAG-3’ |
| *Pde7b* (1353 bp) | 5’-GCTAGCATGTCTTGTTTAATGGTTGAGAGGTGTGGCGAAGTCTTGTTTGAGAATCCTGAGCAGAATGTCAAATGTGTTTGCATGCTAGGAGATGTACGACTAAGGGGTCAGACGGGGGTTCCTGCCGAACGCCGCGGCTCCTACCCGTTCATTGACTTCCGTCTACTTAACAATACAACACACTCAGGGGAAATTGGCAGCAAGAAAAAGGTGAGGAGACTGTTAAGTTTCCAAAGGCACTTCCATGAATCTAGGCTGCTCCGGGGGATGACACCGCAGGCCCCCCTCCACCTGCTGGACGAAGACTACCTTGGACAAGCAAGGCATATGCTCTCCAAAGTTGGAATGTGGGACTTTGACATTTTCTTGTTTGATCGCTTGACAAATGGGAACAGTCTGGTAACTCTGTTGTGTCACCTCTTCAACTCCCATGGACTCATCCACCATTTCAAGCTTGACATGGTGACCTTACACAGGTTTTTGGTTATGGTTCAGGAAGATTACCACGGCCACAACCCGTACCACAATGCTGTTCATGCAGCTGACGTCACCCAGGCCATGCACTGTTACTTGAAGGAGCCAAAGTTGGCGAGCTTCCTCACACCTCTGGACATCATGCTTGGACTATTGGCTGCAGCAGCTCATGACGTGGACCACCCAGGGGTGAACCAGCCATTTTTGATCAAAACTAACCACCACCTTGCCAACTTGTATCAGAACATGTCCGTACTGGAGAATCACCACTGGCGGTCTACAATCGGCATGCTTCGGGAATCAAGGCTCCTTGCTCATTTGCCAAAGGAAATGACACAGGATATCGAACAGCAGCTGGGCTCCCTGATCTTGGCCACGGACATCAACAGACAGAATGAGTTCCTGACCCGCTTAAAAGCTCACCTCCACAATAAGGATTTGAGACTGGAAAATATACAGGACAGACACTTTATGCTTCAGATCGCCTTGAAGTGCGCTGACATTTGCAATCCTTGTCGAATCTGGGAGATGAGCAAGCAGTGGAGTGAAAGAGTCTGCGAAGAATTCTACAGGCAAGGTGACCTTGAACAGAAGTTTGAACTGGAAATCAGTCCTCTTTGTAATCAACAGAAAGATTCAATCCCTAGCATACAAATTGGTTTCATGACTTACATCGTGGAGCCACTGTTCCGGGAGTGGGCCCGGTTCACTGGGAACAGCACCCTGTCGGAGAGCATGCTAAACCATCTCGCGCACAACAAAGCCCAGTGGAAGAGCCTGCTGTCCAATCAGCACAGACGCAGGGGCAGCGGCCAGGACCCAGCGGGCACGGCGCCTGAGACCCTGGAGCAGACAGAAGGCGCCACGCCCTAACTCGAG-3’ |
| *Sept9* (1710 bp) | 5’-GCTAGCATGGAGAGAGATCGCATCACAGCCTTAAAGAGATCGTTTGAAGTCGAGGAGATTGAGCCGCCGAACTCCACACCACCCCGGAGGGTCCAGACCCCTCTGCTCCGAGCCACGGTGGCCAGCTCCAGCCAGAAATTCCAGGACCTGGGGGTGAAGAACTCAGAGCCTGCTGCTCGCCTTGTAGACACCCTGAGCCAGCGCTCCCCCAAGCCTTCCCTGCGGAGGGTGGACCTGGCAGGGGCCAAGGCGCCTGAGCCCATGTCTCGCCGCACCGAGCTCTCCATTGATATCTCCTCCAAGCAGGTGGAGAGCACAGCCTCAACCCCCGGGCCCTCACGGTTCGGGCTTAAGAGGGCCGAAGTCCTGGGCCATAAGACACCAGAGCCTGTCCCCCGGAGGACGGAAATCACCATTGTCAAGCCTCAAGAGTCAGGGCTCCGCAGGGTAGAGACCCCTGCCTCCAAGGCTCCCGAGGGCTCTGCCATGCCTGTCACCGATGCAGCCCCCAAGAGGGTAGAGATCCAGGTGCCCAAGCCAGCGGAGGCACCCAACTGCCCGCTCCCACCCCAGACCCTGGAGAACTCCGAGGCCCCGATGTCTCAGCTGCAGAGCAGGCTGGAGCCCAGGCCCCCTGTGACTGAGGTCCCATATCGGAACCAGGAAGACTCCGAGGTGGCTCCCAGCTGTGTTGGCGACATGGCTGACAACCCTAGAGATGCCATGCTCAAGCAAGCGCCCGTGTCGAGGAATGAGAAGGCCCCCGTGGACTTTGGCTATGTGGGGATCGACTCCATCCTGGAGCAGATGCGCAGGAAGGCTATGAAACAGGGCTTCGAGTTCAACATCATGGTGGTTGGGCAGAGTGGCCTCGGGAAGTCCACCTTAATCAACACCCTCTTCAAGTCCAAAATCAGCCGGAAGTCGGTGCAACCCATCTCGGAGGAGCGTATCCCCAAGACGATTGAAATCAAGTCCATCACTCACGATATTGAAGAGAAGGGGGTCCGAATGAAGTTGACAGTGATTGACACTCCAGGCTTCGGGGACCACATCAACAATGAGAACTGCTGGCAGCCTATCATGAAGTTTATCAATGACCAGTATGAGAAGTACCTCCAGGAGGAAGTCAATATCAACCGGAAGAAACGCATCCCTGACACCCGCGTCCACTGCTGCCTCTACTTCATCCCAGCCACCGGCCACTCACTCAGGCCCCTGGACATTGAGTTCATGAAGCGCCTAAGCAAGGTGGTGAACATAGTCCCCGTCATTGCCAAGGCTGACACGCTGACCCTGGAGGAGAGGGTCTACTTCAAACAGCGGATCACCTCAGACCTGCTGTCCAACGGTATTGACGTGTACCCCCAGAAGGAGTTTGATGAGGACGCAGAGGACCGACTGGTGAACGAGAAGTTTCGGGAGATGATCCCATTTGCCGTGGTGGGCAGCGACCATGAGTATCAAGTCAATGGCAAGAGGATTCTGGGAAGGAAGACCAAGTGGGGCACCATTGAAGTTGAGAATACCACTCACTGTGAATTTGCCTACCTGCGGGATCTCCTTATCAGGACGCACATGCAGAACATCAAAGACATCACCAGCAACATCCACTTCGAAGCCTACCGTGTGAAACGCCTCAACGAGGGCAACAGTGCCATGGCCAACGGGATCGAGAAGGAGCCGGAAACCCAGGAGATGTAGCTCGAG-3’ |
