# Supplementary material for: Genome-Wide DNA Methylation Analysis of Human Pancreatic Islets from Type 2 Diabetic and Non-Diabetic Donors Identifies Candidate Genes That Influence Insulin Secretion
Source: PLoS Genet. 2014 Mar 6;10(3):e1004160. doi: 10.1371/journal.pgen.1004160 (PMC3945174; doi:10.1371/journal.pgen.1004160)
Supplement: Table S14 — DNA sequences for pyrosequencing forward, reverse and sequencing primers. (DOCX) [file pgen.1004160.s019.docx]

| **Table S14.** DNA sequences for pyrosequencing forward, reverse and sequencing primers. | | | | |
| --- | --- | --- | --- | --- |
| **Gene** | **Probe ID** | **Forward primer** | **Reverse primer (5' biotinylated)** | **Sequencing primer** |
| ***CDKN1A*** | cg21091547 | 5'-GGAGTTATAGAAATAAAGGATGATAAGTAG-3' | 5'-TCCCTATAATTACAACAACTTTATTAACCA-3' | 5'-GGATGATAAGTAGAGAGT-3' |
| ***PDE7B*** | cg27306443 | 5'-TGTTTTTTTTTGTTTGTGGTAATTGATAG-3' | 5'-TCCCTAAATAAATAACACCACTTTTCTC-3' | 5'-GTAATTGATAGTATTTTTTAGTTTA-3' |
| ***SEPT9*** | cg19654743 | 5'-TTTTTTAGTGTGGGAGTGGT-3' | 5'-ACCAAACCCATCTCAATACC-3' | 5'-GTGTGGGAGTGGTTA-3' |
| ***IRS1*** | cg04751089 | 5'-GGAAAGAATAGGAAGGGGTAG-3' | 5'-ATTTAAACCCCTATACCAACATCAATTTCC-3' | 5'-GAATAGGAAGGGGTAGA-3' |
| ***HDAC7*** | cg20995304 | 5'-GAGTAGGAAGGAAAGAAGGTTAAGTTAG-3' | 5'-ACTAAAAACCCTTTTCACCAACAA-3' | 5'-TTTTTTGTTATTTTAAGTTTGAGTA-3' |
| ***THADA*** | cg01649611 | 5'-TTGGATTATAAATTTTTTGTATGGT-3' | 5'-AAAAAAATAACTCACCACCATCAATA-3' | 5'-GATTATAAATTTTTTGTATGGTTTG-3' |
| ***PTPRN2*** | cg15572489 | 5'-AGATATTAGGGAAGATTTATGATATTGATG-3' | 5'-ACCTCTAAATCCCTCTCTCC-3' | 5'-ATTAATTTTAATTAGTAGATTGTT-3' |
